# Beware to ignore the rare: how imputing zero-values can improve the quality of 16S rRNA gene studies results

**DOI:** 10.1186/s12859-022-04587-0

**Published:** 2022-02-07

**Authors:** Giacomo Baruzzo, Ilaria Patuzzi, Barbara Di Camillo

**Affiliations:** 1 Department of Information Engineering, University of Padova, Padua, Italy; 2 Microbial Ecology Unit, Istituto Zooprofilattico Sperimentale Delle Venezie, Padua, Italy; 3 CRIBI Biotechnology Centre, University of Padova, Padua, Italy; 4 Department of Comparative Biomedicine and Food Science, University of Padova, Padua, Italy; 5 Present Address: Research & Development Division, EuBiome S.R.L., Padua, Italy

**Keywords:** Zero-imputation, Sparsity, Normalization, Count preprocessing, 16S rDNA-Seq, Count data, Count simulation, Benchmarking

## Abstract

**Background:**

16S rRNA-gene sequencing is a valuable approach to characterize the taxonomic content of the whole bacterial population inhabiting a metabolic and spatial niche, providing an important opportunity to study bacteria and their role in many health and environmental mechanisms. The analysis of data produced by amplicon sequencing, however, brings very specific methodological issues that need to be properly addressed to obtain reliable biological conclusions. Among these, 16S count data tend to be very sparse, with many null values reflecting species that are present but got unobserved due to the multiplexing constraints. However, current data workflows do not consider a step in which the information about unobserved species is recovered.

**Results:**

In this work, we evaluate for the first time the effects of introducing in the 16S data workflow a new preprocessing step, zero-imputation, to recover this lost information. Due to the lack of published zero-imputation methods specifically designed for 16S count data, we considered a set of zero-imputation strategies available for other frameworks, and benchmarked them using in silico 16S count data reflecting different experimental designs. Additionally, we assessed the effect of combining zero-imputation and normalization, i.e. the only preprocessing step in current 16S workflow. Overall, we benchmarked 35 16S preprocessing pipelines assessing their ability to handle data sparsity, identify species presence/absence, recovery sample proportional abundance distributions, and improve typical downstream analyses such as computation of alpha and beta diversity indices and differential abundance analysis.

**Conclusions:**

The results clearly show that 16S data analysis greatly benefits from a properly-performed zero-imputation step, despite the choice of the right zero-imputation method having a pivotal role. In addition, we identify a set of best-performing pipelines that could be a valuable indication for data analysts.

**Supplementary Information:**

The online version contains supplementary material available at 10.1186/s12859-022-04587-0.

## Background

The study of microbial communities has deeply changed since it was first introduced in the seventeenth century [1]. When the pivotal role of microbes in regulating and causing human diseases became evident, researchers began to develop a variety of techniques to isolate and grow microbes in the laboratory, with the aim of characterizing and classifying them. Today, microbial community profiling is almost uniquely performed by sequencing the DNA content of the community by means of Next-Generation Sequencing (NGS) technologies. When the focus of the study is on the bacterial community, the two main approaches in this framework are shotgun metagenomics, based on the sequencing of the whole genomic content of the community [2], and 16S rRNA gene sequencing (16S rDNA-Seq), a targeted sequencing of the gene that codes for the 16S subunit of prokaryotic ribosome (16S rRNA gene) [3]. This gene plays an essential role in prokaryotic life; it is ubiquitous to all bacteria, and its DNA sequence is characterized by both highly conserved and variable regions, which allows distinguishing among species. For this reason, it is used as a sort of molecular fingerprint for assigning to each community member a taxonomic characterization. The constant improvement of NGS platforms, able to produce a higher and higher amount of data reducing the related time and costs, allowed researchers to progressively raise the sampling size in their experiments. However, shotgun metagenomics still remains far more cost- and resource-demanding than the targeted amplicon alternative. This ensured 16S rDNA-Seq an increasing growth in election rate as preferred methodology to perform microbiome studies. After sequencing, 16S microbial community data are typically summarized into large matrices where the columns represent samples and the rows contain operational taxonomic unit (OTU) [4] or amplicon sequence variant (ASV) [5] count values, that represent (broadly speaking) bacteria types. Throughout the manuscript, we will use the terms “OTU”, “ASV” and “feature” interchangeably to identify the rows of count matrices.

As pointed out in Weiss et al. work [6], several characteristics of OTU tables can cause incorrect results in downstream analyses, if ignored. First, the total microbial community in each biological sample may be represented by very different amount of sequences (i.e., library sizes), sometimes differing by several orders of magnitude, reflecting differential efficiency of the sequencing process rather than real biological variation. Second, 16S rDNA-Seq count matrices are typically highly sparse (70–95% of null values). Third, they are compositional [7–10], a characteristic that is not usually taken into account in the current approach to data analysis. As extensively explained in Gloor et al. work [10], data obtained from high-throughput sequencing (HTS) of 16S rRNA gene amplicons are compositional because each sample counts have an arbitrary total, the sequencing depth, imposed by the sequencing instrument; consequently, the read counts observed in a HTS run are random samples of the relative abundance of the molecules in the original sample.

All these elements affect the measured composition of the bacteria population, resulting in altered abundances and undetected species. To mitigate the effect of these three aspects and to avoid misleading results, data should be treated prior to performing downstream analysis. This inherently poses the problem of choosing the most appropriate tools to correctly perform the preprocessing step.

For the above mentioned reasons, the usual analysis workflow starts with a preprocessing step called normalization. Normalization is the process of eliminating artefactual systematic biases between samples, making possible a direct comparison of species abundance between them or between groups of them. Raw data can indeed contain peculiar biases due to sample collection, library preparation and sequencing process that can imply uneven sampling depth and sparsity. Many downstream analyses, such as ordination analysis and statistical testing performed to look for specific bacteria that are differentially abundant between two ecosystems, may suffer from these experimental biases in such a heavy way that incorrect conclusions may be drawn if normalization is not previously applied [11].

It is noteworthy, however, that normalization cannot solve or even diminish data biases linked to the compositionality and high sparsity of sequencing count data. The null values affecting these data may rise from a multiplicity of factors, but they may be attributed to two main sources: a biological and a technical one. Biological zeros are those null values present into a sample count distribution representing features that are actually not present in the sample. These zeros are constitutive of each sample population profiling and represent a true information of absence of some species within the sample. In contrast, technical zeros are those null values that characterize unseen features within the sample, i.e. features that were present in the sampled population and whose information got lost during sequencing procedure due, for example, to their low abundance with respect to other sample components. Microbial populations indeed typically show a strongly skewed internal distribution, with a high number of rare species and a limited number of highly present species [12]. This fact, jointly with the finite number of reads obtainable from sequencing instruments, causes rare species loss at a rate that is heavily dependent on both the internal microbiome distribution and the number of samples sequenced in the same sequence run (the so-called multiplexing level). This loss of information may occur in different steps of a sequencing study, such as amplification, library preparation and, of course, during the proper sequencing step. A recent research work by Zhang et al. [13] on single-cell RNA sequencing (scRNA-Seq) data focused on evaluating the performance of a set of methods for missing value imputation proposed in different contexts, when applied to the scRNA-Seq field. In particular, the authors aimed at verifying if some of the tested tools were appropriate for imputing zero values and restoring the original structure of data as precisely as possible. This preprocessing step is known as “zero-imputation” and has now become a fundamental issue in scRNA sequencing research [13, 14]. It is noteworthy that, even if 16S count data suffer from analogous issues as scRNA-Seq data do, a huge scientific effort is now being made to find an appropriate zero-imputation strategy in scRNA sequencing framework [13, 14], while current 16S rDNA-Seq count data workflow does not consider a step in which the problem of species that became unobserved during data generation is treated.

It should also be considered that until some years ago the vast majority of microbiota analyses followed the general advice introduced by Bokulich et al. work [15] according to which a conservative threshold on proportional abundance of 0.005% should be used for OTU filtering for data sets where a mock community was not included for calibration. This conclusion was, however, drawn by the authors based on OTU tables produced by the first version of the 'quantitative insights into microbial ecology' (QIIME) [16] pipeline. Recent advances in the field made it possible to control errors at the point that ASVs can be determined with higher precision, down to the level of single-nucleotide differences, with benefits related to both finer resolution in taxa discrimination and reproducibility of the results [17]. In 2017 it was proposed [17] and widely accepted by the community a gradual switch from OTUs to ASVs. Two years later, a new version of the QIIME pipeline [5] was published and it is currently the most used tool for count table creation via DADA2 [18] or Deblur [19] approach. A direct consequence of this change is that low abundant features stored in present-day ASV tables are inherently more reliable than the ones included in old OTU tables. Therefore, the application of imputation to this more reliable data could be of great relevance, especially in those studies that focus their attention on rare species or may find in low abundance features new, unexplored reading keys.

The present work has the main objective to test and measure the effects of preserving low abundance ASVs information and also to use this part of data to perform a loss-information recovery step (zero-imputation). To the best of our knowledge only benchmarks considering the normalization step are available in the literature [6], whereas no effort was done so far to test the potential benefits of introducing the zero-imputation step and to compare the applicability of dedicated tools available for information loss recovery.

Secondly, we aim at identifying optimal pipelines that fill the above gaps in order to assure solid and reliable conclusions from 16S rDNA-Seq (“metataxonomic”) data analyses, and to give researchers some indications in the identification of the most appropriate preprocessing approaches to conduct metataxonomic data analyses.

In the present work, a collection of normalization and zero-imputation approaches is tested for 16S rRNA-gene sequencing data preprocessing. This permits to (i) compare an updated list of normalization tools considering also the most recent publications [20] and (ii) evaluate the effect of introducing zero-imputation step in the 16S rDNA-Seq preprocessing workflow highlighting the importance of choosing the correct approach to perform it.

## Methods

In this section we provide a description of the datasets used for methods assessment (section “Datasets”) and the complete list of zero-imputation and normalization tools included in this study (sections “Imputation methods” and “Normalization methods”, respectively). Last, we describe how tools were combined into 35 different 16S preprocessing pipelines (section “Pipelines”) and how the different pipelines were evaluated (section “Evaluation criteria”).

### Datasets

The central aims of the present study were to test for the possible advantages of introducing the zero-imputation step in metataxonomic data analysis and to evaluate a set of tools for count data preprocessing. To do that, we used synthetic data generated using the recently released metaSPARSim simulator [21]. Starting from an intensity vector describing each specific experimental condition average effect, this 16S rDNA-Seq count data simulator produces the count data following a two-steps approach. First, species abundances varying between biological replicates are modelled using a gamma distribution; second, the technical variability originated by the sequencing process is modelled using a Multivariate Hypergeometric model. Data produced after the first step are here considered as the ground truth for real (unobserved) relative abundances in the samples, whereas data produced after the second step are the simulated raw count matrix, i.e. the final output that reflects the information acquired with the (simulated) sequencing experiment. This matrix will consequently carry the information about the analysed samples plus the bias introduced by the sequencing step that, as also described in [21], acts on the original community composition (first step data) as a sampling process.

Since metaSPARSim parameters can be estimated from real datasets, we simulated three different benchmarking datasets to assess the considered preprocessing pipelines in different reality-inspired scenarios (Table 1). The benchmarking datasets were simulated to contain only biological replicates, since in practice technical replicates are limited to very specific applications. The simulated datasets used within this benchmarking procedure are released together with this work (see *Data Availability* section). In the following, the details about the three synthetic datasets are reported.

- **Dataset 1:** this dataset was composed by 14 experimental conditions (groups) and mimics experimental data from animal gut 16S rDNA-Seq. This dataset was simulated starting from the preset named “R1” present in metaSPARSim internal archive and was produced to consider a “mean difficulty” scenario characterised by limited sparsity level and low variability among replicates. In particular, the simulated dataset is characterized by a ground truth sparsity of 63.03% and raw count matrix sparsity of 72.56%.
- **Dataset 2:** the second dataset was included to assess tools performance in a low sparsity but high biological variability scenario. Indeed, these data are simulated based on the Human Microbiome Project (HMP) [22, 23] original dataset, that was characterized by a very high biological variability due to the fact that replicates within the conditions were constituted by samples collected from different individuals. metaSPARSim preset “R3” was selected for the present study considering this characteristic as bearer of data features that could be deeply challenging for preprocessing tools. The obtained synthetic ground truth dataset presents a sparsity of 56.61%, while raw data sparsity level is 67.91%.
- **Dataset 3:** the last dataset considered for the present study was chosen to include an example mimicking higher budget experiments in which low multiplexing levels can be chosen and, consequently, almost all the information can be caught (little sequencing loss of information). This permits to assess tools performance in a situation in which unnecessary imputation could introduce some bias. This dataset was obtained from a metaSPARSim preset (called “R2”) that was derived from data describing raw milk cheese microbiota during ripening and is characterized by a raw total sparsity of 94.34%, with the ground truth sparsity level being 91.26%.

**Table 1:** Simulated datasets characteristics

	Dataset 1	Dataset 2	Dataset 3
Groups	14	8	12
Samples	140	80	120
Replicates	10	10	10
Features	3326	758	1140
Sequencing depth (range)	16,347–995,050	2763–97,612	30,165–293,285
Sparsity level	72.56%	67.91%	94.34%

With “Groups” we indicate experimental conditions, while with “Replicates” we identify the samples belonging to the same experimental condition

### Imputation methods

The selected tools were all developed for unseen information recovery, but in very different frameworks, such as scRNA-Seq, microarray and compositional data analysis. Indeed, to the best of our knowledge no published tools are available that are specific for 16S data zero-imputation. Five different zero-imputation approaches will be presented in the following. Methods were tested as released from the developers and following user guide indications.

**DrImpute** [24] is a zero-imputation tool for scRNA-Seq data that recovers information on null values imputing them by borrowing information from similar samples, starting from normalized and log-transformed count data. DrImpute first identifies similar samples based on clustering, and imputation is then performed by averaging the values from similar samples. To achieve robust estimations, the imputation is performed multiple times using different sample clustering results followed by averaging multiple estimations for definitive imputation. First, the sample-sample similarity matrix is computed using Spearman and Pearson correlations, followed by the sample-wise clustering based upon the distance matrix over a range of expected number of clusters $k$. For each combination of distance metric (Spearman or Pearson) and $k$, the recovered values in the input matrix are estimated. The averaged estimation over all combinations then gives the final imputed values.

**scImpute** [25] is another recently published tool for scRNA sequencing preprocessing that automatically identifies zeros that may likely correspond to information loss, and only performs imputation on these values without modifying the remaining data. To achieve this goal, scImpute first learns each feature’s dropout probability in each sample based on a mixture Gamma-Normal model, and then uses the obtained statistical model to systematically determine whether a zero value comes from a dropout event or not. Next, it imputes the (highly probable) dropout values in a sample by borrowing information of the same feature in other similar samples, which are selected based on the features unlikely affected by dropout events and then labelled as reliable source of information.

Contrarily to DrImpute and scImpute, **LLSimpute** [26] is a zero-imputation tool for microarray data that tries to estimate missing values using sample information stored in co-abundant or similar features. It starts by a linear regression model, in which (for each feature *i*) samples are divided into a group in need of imputation (*C*_*i*_) and a group collecting the non-null values (*D*_*i*_). If we suppose there are *q* missing values for feature *i*, it finds the *K*-nearest neighbour feature vectors for feature *i* based on values in *D*_*i*_, which may be represented as $G_{{K_{i} D_{i} }} \in R^{{Kx \left( {n - q} \right)}}$, where *n* is the total number of samples. The imputation is then performed by considering the minimization:

1 $$\min_{x} \left\| {G_{{K_{i} D_{i} }}^{T} x - G_{{i, D_{i} }} } \right\|_{2}$$

where $G_{{i, D_{i} }}$ denotes feature abundance of feature $i$ across samples belonging to $D_{i}$ set, and recovering values by calculating $G_{{K_{i} C_{i} }}^{T} x$, where $G_{{K_{i} C_{i} }}$ represents abundance levels of features $K_{i}$ across samples $C_{i}$.

**zCompositions** [27] is a tool specifically addressing compositional data sets [28, 29], i.e. those datasets composed by discrete vectors representing the numbers of outcomes falling into any of several mutually exclusive categories. Martin-Fernandez and collaborators propose [30] a Bayesian imputation method for zero counts based on multiplicative replacement, starting from a typical multinomial modelling of count data and a Dirichlet distribution as its conjugate prior. Using this methodology one can retrieve lost values preserving the ratios between non-zero components in the samples. This strategy consists of replacing the zero values with their posterior expectation, while non-zero proportions are multiplied by a factor according to the number of zero counts. In the tool, several multiplicative Bayesian imputations are implemented that differ both for the prior distribution used to model the random vector of multinomial probabilities and for the replacement of zero values. Among these, the most promising seem to be the Geometric Bayesian Multiplicative (GBM) approach, Square root (SQ) Bayesian Replacement and Rounded zero multiplicative replacement (CZM). The details about the three approaches can be found in the original work. The SQ and CZM approaches were included in this study (in the following named “zCompositions_SQ” and “zCompositions_CZM”), while we excluded the GMB approach. Despite GMB approach showed very good performance in the original paper [27], we had to exclude it from the benchmarking since this method does not work when features appear only in a unique sample throughout the entire count data matrix, a situation that is not infrequent in 16S rDNA-Sequencing data analysis.

### Normalization methods

A plethora of tools became available in the last years to perform sequencing count data normalization. In this work, a subset of them has been selected for performance testing on 16S data, these tools being the most widely used or the most recent and promising now available. This collection is formed by approaches that nowadays we can call "historical", such as Total Sum Scaling, by more recent techniques, such as Cumulative Sum Scaling and the ones implemented in *edgeR* and *DESeq2 R* packages, and by a more recently developed tool, such as *GMPR*, that directly address data heavily affected by sparsity. In the following, the five normalization methods are described in detail.

**Total sum scaling (TSS)** or global scaling [31] is the simplest and oldest way of normalizing sequencing data. It simply divides raw counts by the total number of reads found in the sample (i.e. the *sequencing depth*), i.e. it transforms count vectors in the corresponding vectors of proportions within the sample by computing $p_{ij} = c_{ij} /N_{j}$, where $c_{ij}$ are the counts of *i*th feature in the *j*th sample and $N_{j} = \mathop \sum \limits_{i} c_{ij}$ is *j*th sample sequencing depth. This notation will be used within all the following method descriptions.

Because of its simplicity, for the present study this method was not borrowed from any implemented *R* package, but it was directly calculated on raw data with basic *R* functions.

**Cumulative sum scaling (CSS)** performs a quantile normalization by looking for a data-specific quantile to use in order to normalize data in a coherent way. This method has been introduced by Paulson et al. [32] and then included in *metagenomeSeq* [33] *R* package. The developers propose this normalization technique to correct for sequencing bias, that is thought to come from features that are preferentially amplified in a sample-specific way. If we denote by $$q_{j}^{l}$$ the *l*th quantile of sample *j* and by $$s_{j}^{l}$$ the sum of counts ($$c_{ij} )$$ for sample *j* up to the *l*th quantile, the normalization method selects a value $$\hat{l} \le m$$, where $$m$$ is the total number of features, to define a normalization scaling factor for each sample to produce normalized counts:

2 $$\widetilde{{c_{ij} }} = K\frac{{c_{ij} }}{{s_{j}^{l} }}$$

where $K$ is an appropriately chosen constant applied to all samples so that normalized counts have interpretable units. The authors suggest this $K$ to be chosen as the median of scaling factors $s_{j}^{{\hat{l}}}$ across samples. To determine the most appropriate value for $\hat{l}$, an adaptive, data-driven method is used. It finds a value $\hat{l}$ for which sample-specific count distributions deviate from an appropriately defined reference distribution. In particular, if we consider $\overline{q}^{l} = med_{j} \left( {q_{j}^{l} } \right)$ the median *l*th quantile across samples as the *l*th quantile of the reference distribution and denote with $d_{l} = med_{j} |q_{j}^{l} - \overline{q}^{l}$| the median absolute deviation of sample-specific quantiles around the reference, $\hat{l}$ can be identified as the smallest value for which high instability is detected in high quantiles of $d_{l}$, i.e. the smallest $l$ that satisfies $d_{l + 1} - d_{l} \ge 0.1 \cdot d_{l}$.

**edgeR**’s normalization procedure [34], namely "trimmed mean of M-values normalization method" (TMM), is one of the most famous and widely used normalization techniques in sequencing data preprocessing. Its native framework was bulk RNA-sequencing, but it has been used in a huge variety of other situations involving sequencing count data, such as metagenomic and single cell RNA-sequencing studies [35, 36]. Robinson and his collaborators proposed [37] an empirical strategy that equates the overall expression levels of features between samples under the assumption that the majority of them are not differentially abundant. For sequencing data, they define the feature-wise log-fold-changes as:

3 $$M_{i} = \log_{2} \frac{{\frac{{c_{ij} }}{{N_{j} }}}}{{ \frac{{c_{ik} }}{{N_{k} }}}}$$

and absolute presence levels as:

4 $$A_{i} = \frac{1}{2}\log_{2} \left( {\frac{{\frac{{c_{ij} }}{{N_{j} }} \cdot c_{ik} }}{{N_{k} }}} \right) , \quad {\text{for}}\quad c_{i.} \ne 0$$

Normalization factors are calculated starting from the trimmed mean (i.e. the average after removing an upper and lower fixed percentage of the data) of M- and A-values, then calculating the weighted mean of $M_{i}$, with weights as the inverse of the approximate asymptotic variances (calculated using the delta method [38]). The cases where $c_{ij} = 0$ or $c_{ik} = 0$ are preventively trimmed since log-fold-changes cannot be calculated.

Another very well-known tool for RNA-sequencing analysis is **DESeq2** [39], which performs count data normalization and differential analysis. Also in this case, normalization is done through data scaling for sample-specific size factors. To estimate these size factors, DESeq2 package implements the median-of-ratios method already used in its first version, DESeq [40]. Following this method, size factors $s_{j}$ for each sample are estimated as:

5 $$\widehat{{s_{j} }} = median_{i} \frac{{c_{ij} }}{{ \left( {\mathop \prod \nolimits_{v = 1}^{n} c_{iv} } \right)}}$$

where *n* is the total number of samples. Also in this approach, $c_{ij} = 0$ are excluded from the computation. The denominator of this expression can be interpreted as a pseudo-reference sample obtained by taking the geometric mean across samples. Thus, each size factor estimate is computed as the median of the ratios of the *j*th sample counts to those of the pseudo-reference.

**GMPR** is a recently published tool [20] that proposes a novel inter-sample normalization method, named geometric mean of pairwise ratios (GMPR), developed specifically for zero-inflated sequencing data such as 16S rRNA-gene sequencing data. In detail, in a first step GMPR procedure calculates the median count ratio of nonzero counts between samples *j* and *k*,

6 $$r_{jk} = median y \in \left\{ {1, \ldots ,m} \right\}|c_{yj} \cdot c_{yk} \ne 0 \left\{ {\frac{{c_{yj} }}{{c_{yk} }}} \right\}$$

where *m* is the total number of features. Then, in a second step the size factor *s*_*j*_ for a given sample *j* is calculated as

7 $$s_{j} = \left( {\mathop \prod \limits_{k = 1}^{m} r_{jk} } \right)^{\frac{1}{m}} , j = 1, \ldots , n$$

where *n* is the total number of samples. Based on this strategy, the tool uses far more information than both TMM and DESeq strategies, which are usually restricted to a small subset of OTUs due to the a priori exclusion of null values.

### Pipelines

The tools previously introduced were combined to form 35 different preprocessing pipelines. The first group of pipelines represents the most frequently adopted approach to analyse 16S data that consists solely in the normalization step. The five approaches and tools that compose this first group are the ones previously presented (subsection “Normalization methods”), and will be referred to as TSS, CSS, edgeR, DESeq2 and GMPR in the following. The second group of pipelines represents the five imputation methods considered in this work (subsection “Imputation methods”), and will be referred to as DrImpute, scImpute, LLSimpute, zCompositions_SQ and zCompositions_CZM. Finally, the last group of pipelines is composed by all the combinations of the previous normalization and zero-imputation approaches. Even if some imputation tools explicitly asked for raw or normalized data, the performance of each tool was considered when used both singularly and in combination with a normalization step, to investigate if some normalization technique could affect positively even tools initially designed to deal with raw data.

Both the simulated and those preprocessed by applying the above mentioned pipelines are made available as RData files within an ad-hoc R package for all the simulated datasets (see *Data Availability* section).

### Evaluation criteria

**Fig. 1:**
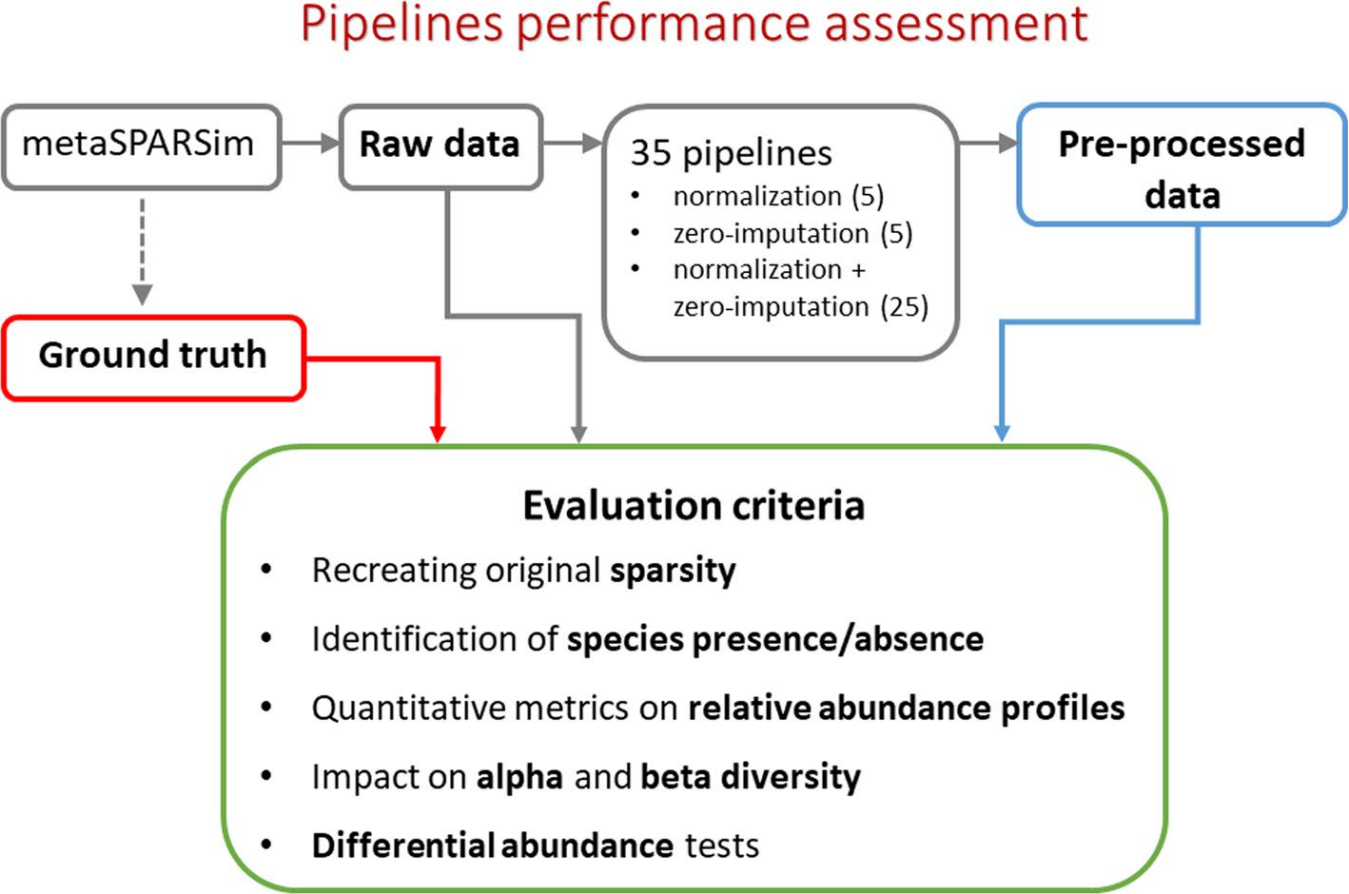
Benchmarking framework. Graphical representation of the main data and evaluation criteria used in this study

The adopted benchmarking framework, represented in Fig. 1, involved ground truth data jointly with raw and preprocessed data. Starting from a preset (see *Datasets* section), ground truth and raw count tables were produced by the use of metaSPARSim tool. Then, raw matrices were preprocessed with all the 35 pipelines, that include 5 normalization-only and 5 zero-imputation-only pipelines, and 25 pipelines combining all the normalization and zero-imputation methods. Finally, ground truth, raw and preprocessed data were evaluated accordingly to a set of criteria that are explained in the following.

#### Total sparsity

As a first metric, the pipelines were evaluated for their ability to recreate the original data sparsity. Each pipeline results were compared to the ground truth in terms of percentage of zero counts, i.e. the ratio between the number of zero counts and the total number of count matrix entries.

#### Species presence/absence

The zero values in the raw count matrix can be biological zeros (species that were not present in the sample) or technical zeros (species that were present in the sample but got undetected). After the imputation step, a zero entry in the OTU table can remain zero (i.e. the zero-imputation tool identifies it as a biological zero) or can become a value greater than zero (i.e. the zero-imputation tool identifies it as a technical zero and imputes it). To assess the ability of the pipelines in recovering the lost information about undetected species, we defined.

- **TP (True Positive)** as the true technical zeros that were correctly identified as technical zeros (and so imputed)
- **TN (True negative)** as the true biological zeros that were correctly identified as biological zeros (and so not imputed)
- **FP (False Positive)** as the true biological zeros that were incorrectly identified as technical zeros (and so imputed)
- **FN (False Negative)** as the true technical zeros that were incorrectly identified as biological zeros (and so not imputed)

The confusion matrix shown in Table 2 summarises these definitions.

**Table 2:** Confusion matrix

	Actual zero meaning	
	Technical zero	Biological zero
Imputed zero meaning		
Technical zero (i.e. > 0 after imputation)	TP	FP
Biological zero (i.e. 0 after imputation)	FN	TN

TP, True positive; FP, False Positive; FN, False Negative, TN, True Negative

The performance of the tools was assessed using two metrics commonly used in classification problems: sensitivity (i.e. TP/(TP + FN)) and specificity (i.e. TN/(TN + FP)). **Sensitivity** measures the percentage of technical zeros that were correctly detected, and it is useful to measure the amount of lost information (undetected species) that was correctly recovered by zero-imputation. **Specificity** measures the percentage of biological zeros that were correctly detected (i.e. not imputed), and it is useful to monitor that zero-imputation is not recovering species that are actually missing in the sample.

#### Relative abundance profile

The assessment scores presented in the previous paragraphs do not allow measuring the performance of the tools in identifying the correct entry to impute (or to not impute) since they do not give any indication about the correctness of the imputed values. To assess the ability of the methods to reconstruct "true", i.e. ground truth based, proportional abundances, two different metrics were considered: Symmetric Mean Absolute Percentage Error (SMAPE) and Aitchison distance [41]. The first one is a quantitative metric based on percentage (or relative) errors. More precisely, it quantifies the error between two vectors *x* and *y* of length *m* as:

8 $$SMAPE\left( {x,y} \right) = \frac{100}{m}\mathop \sum \limits_{i = 1}^{m} \frac{{\left| {x_{i} - y_{i} } \right|}}{{\left| {x_{i} } \right| + \left| {y_{i} } \right|}}$$

The i-th element in the SMAPE was set equal to 0 when both the ground truth and the imputed value were null. For each sample, the ground truth, the raw and the preprocessed data were compared using SMAPE after Count Per Million (CPM) normalization [34]. SMAPE was chosen as an alternative to the classical relative error because of heavy data sparsity. In fact, when the reference value in the relative error formula is zero this measure becomes undefined.

Aitchison distance, on the other hand, accounts for the compositional nature of sequencing data [10]. This distance was used because compositional data are constrained by the simplex and are not in the Euclidean space; therefore, Euclidean distance is not applicable because a dependency structure between OTUs (also called "parts" in compositional analysis framework) is present. The formula to calculate the Aitchison distance between two generic vectors *x* and *y* of length *m* is:

9 $$d_{A} \left( {x,y} \right) = \sqrt {\frac{1}{m}\mathop \sum \limits_{i = 1}^{m - 1} \mathop \sum \limits_{j = i + 1}^{m} \left( {\ln \frac{{x_{i} }}{{x_{j} }} - \ln \frac{{y_{i} }}{{y_{j} }}} \right)^{2} }$$

As for SMAPE, we computed Aitchison distance on CPM transformed data.

For each dataset and for each pipeline, distributions of SMAPE and Aitchison distances between ground truth and preprocessed data were compared. The goal was identifying pipelines that achieved SMAPE and Aitchison distances significantly lower in preprocessed data with respect to raw data or normalized-only data. Please note that raw data and normalized-only data achieve the same SMAPE and Aitchison distances, since normalization methods do not alter feature relatives abundance. Therefore, this analysis aims at revealing if pipelines involving a zero-imputation step are able to reconstruct sample relative abundances closer to real ones compared with raw data or normalized-only data.

This was performed by means of a one side Mann–Whitney paired U test with Benjamini–Hochberg FDR correction [42] and significance threshold 0.05.

In addition, as suggested by Sullivan and Feinn [43], an effect size calculation was coupled with the above statistical test to measure the magnitude of possible significant differences between distributions. Effect size results were then compared using cut-offs for magnitudes interpretation, as initially suggested by Cohen [44] and subsequently expanded by Sawilowsky [45] (Table 3).

**Table 3:** Cohen's d cut-offs

Effect Size	Cohen’s *d*	References
Negligible	< 0.01	Sawilowsky [45]
Very small	0.01	Sawilowsky [45]
Small	0.20	Cohen [44]
Medium	0.50	Cohen [44]
Large	0.80	Cohen [44]
Very large	1.20	Sawilowsky [45]
Huge	2.0	Sawilowsky [45]

#### Impact on bacterial diversity

One of the most immediate aspects to look at when performing a microbiome analysis is the population diversity. Diversity indices, created for this purpose, are quantitative measures used to investigate the population structure or to detect differences in the composition of the ecological niches between different conditions. However, as well described by Finotello et al. [46], diversity is not a determined physical quantity for which a consensus definition and a unique unit of measure have been established, and several diversity indices are currently available. These indices are generally divided into two conceptually different categories: the species diversity in sites belonging to a niche (alpha diversity), and the differentiation among those sites (beta diversity). Despite some works having attempted to sum alpha and beta diversity into a measurement of total diversity [47, 48], the standard practice is to use alpha and beta diversity indices independently, to estimate intra- and inter-samples diversity, respectively. In particular, the first is used to describe the compositional complexity of a single sample, whereas the second is commonly intended as a measure of differences between samples. Also within the same category of alpha or beta diversity, different indices have different purposes and mathematical formulations, and this implies that the use of more than one index is recommended to look at different population characteristics when performing a bacterial population analysis.

In this work, two alpha and two beta diversity measures were considered to assess the effect of each preprocessing pipeline on microbial community composition, with the aim of identifying the ones most preserve the real structure of the ground truth data.

##### Alpha diversity

Alpha diversity indices are classifiable into three main categories: richness indices, which estimate the number of different species in a sample; evenness indices, which consider the species relative abundances, without considering their total number; and diversity indices, which account for both the species relative abundances and their total number.

For the first category, an index called ‘**observed richness**’ (here denoted as ${S}_{obs}$) was considered, which provides a direct measure of population complexity by simply counting the number of different species present in a sample and is, consequently, inherently linked with data sparsity. Different richness indices are available in the literature that correct observed richness for the number of hypothetically not-observed species (e.g. Chao1 [49] or first- and second-order Jackknife indices [50]). However, we did not consider them because this kind of richness estimators are usually valid only if applied on (integer) count data, while normalized and imputed data considered within this study are transformed data which may no longer have the ‘count’ nature.

As regard evenness indices, the so-called ‘**Pielou index**’ was chosen as a measure to evaluate samples evenness. This index considers the logarithm of the Hill number [51] of the first order and divides it by the logarithm of total observed species. Consequently, it varies between 0 and 1, reaching its maximum value when all the species are equally abundant within the studied community.

Finally, many popular diversity indices that differ in their theoretical foundation and interpretation were considered for inclusion in this study, e.g. **Shannon entropy (H)** [52], inverse **Simpson index (IS)** [53] or the more recent **Tail statistic (T)** [54]. However, we decided to discard these and all other possible diversity indices from our evaluation because of their “mixed” nature that combines both richness and evenness information. Indeed, diversity indices are composed measures that are very used in microbiota analyses to have a unique value describing both population size and equitability at the same time, but for this same reason they inherently mix the effects of the two properties. This makes this family of indices optimal for real analysis contexts, but not suitable to characterize precisely the effects of data preprocessing.

To measure the ability of each preprocessing pipeline to achieve alpha diversity indices closer to the true ones we computed the relative error between alpha indices in preprocessed data vs ground truth data. Then, we compared this relative error with the one obtained considering alpha indices in raw vs. ground truth data. The comparison was performed using a one-sided non-parametric paired Mann–Whitney U test (p value < 0.05 after Benjamini–Hochberg FDR correction for multiple testing [42]) on the relative errors described above. Since normalization does not affect alpha indices computation, pipelines involving only a normalization step will achieve the same relative errors observed in raw data. Therefore, pipelines performing better than raw data will also perform better than normalization-only strategies, and this latter comparison is not explicitly assessed.

In the literature, statistical tests comparing alpha values of samples in different groups are routinely used to assess differences in composition between groups. Therefore, we decided to evaluate the impact of different pipelines also in terms of conclusions derived from this analysis. In particular, we performed a one-sided nonparametric Mann–Whitney U test, to detect both the statistical difference and the direction of change of alpha indexes between pairs of groups in a dataset. The above statistical procedure was performed on alpha diversity indices computed on ground truth data, on raw data and on each of the 35 preprocessed data, and results were then compared.

##### Beta diversity

As regards beta diversity, two metrics were considered: **Whittaker beta diversity** [55] that uses presence-absence data measuring beta diversity as the ratio between the number of different species in a group and the number of different species in a sample, and **Bray–Curtis dissimilarity** [56] that exploits the quantitative information of internal microbial community composition, and is expressed as 1 minus twice the ratio between the sum of the lesser values for only those species in common between both sites and the total number of specimens counted at both sites.

Typically, beta diversity indices values are used to analyse differences across samples in a visual way, such as heatmaps or scatterplots. Therefore, we decided to evaluate the different pipelines looking at beta diversity indices visualizations and comparing them with the ones obtained on ground truth data. More in details, Whittaker dissimilarity values were graphically represented using heatmaps, whereas Bray–Curtis dissimilarity values were used to build distance matrices on which Non-metric Multidimensional Scaling (NMDS) dimensionality reduction was performed and the first two dimensions were plotted.

#### Differential abundance analysis

Differential abundance analysis is a fundamental step in microbiome studies. It consists in identifying features that differ in their abundance between two sample categories, e.g. the body site from which a sample was collected or the geographical area from which a soil sample was taken. Differentially abundant features are usually identified using (generic or specific) statistical tests that check for possible significant differences between groups of samples. This step is well-implemented in some available R packages for metagenomic data analysis; however, in this work we chose to perform this analysis using a non-parametric Mann–Whitney U test. This decision was based on the fact that each differential analysis tool has its own assumptions and underlying model; a neutral evaluation method that was independent on the choice (and goodness) of data modelling was then preferred in this evaluation framework in order not to add further potential biases to the results.

In particular, for each feature in the ground truth, the raw and the preprocessed datasets, a Mann–Whitney U test was performed between data coming from different conditions (groups). Differentially abundant features were identified for each couple of conditions by selecting the resulting p-values that fell under the significance level of 0.05 after Benjamini–Hochberg FDR correction for multiple testing. Then, as a summary measure of concordance between ground truth and all other datasets results for each comparison, Jaccard index (also known as Intersection-Over-Union) [57] was used:

10 $$I_{Jaccard}^{ab} = \frac{{GT_{ab} \cap D_{ab} }}{{GT_{ab} \cup D_{ab} }}$$

where $GT_{ab}$ is the set of differentially abundant features for conditions *a* and *b* of ground truth data and $D_{ab}$ is the correspondent set of features identified as differentially abundant in the generic raw or preprocessed dataset $D$.

A set of Jaccard values for concordance with ground truth was obtained from the pairwise group comparisons for each dataset. Jaccard indices obtained on raw and preprocessed data were compared using a paired Mann–Whitney u test, coupled with an effect size calculation.

## Results

Results obtained from the comparison of the different analysis pipelines are shown in the following. Results are aggregated based on the imputation method included in the pipelines. For each imputation method, the results are reported in terms of average value across the 6 pipelines making use of the specific zero-imputation tool, i.e. the 6 pipelines composed by the 5 normalization methods or no-imputation, followed by the specific imputation tool. For example, the term “scImpute pipelines” will indicate the 6 pipelines: scImpute, CSS + scImpute, TSS + scImpute, edgeR + scImpute, DESeq2 + scImpute and GMPR + scImpute. This choice of aggregating results based on the imputation method is done because the choice of the imputation seems to affect results more than the choice of the normalization tool, which, on the other hand, has a limited effect on the results. Complete results are reported in Supplementary Materials, where the reader can find specific results obtained for each single pipeline.

Here and in the following, the simulated raw count matrix will be referred to as "raw", whereas the ground truth data obtained from metaSPARSim for comparison are referred to as "true" values. We recall that these data represent pre-sequencing abundance values, i.e. "true" abundances in samples prior to sequencing. Pipelines results will be discussed in terms of improvement compared with no preprocessing (i.e. raw data) or normalization-only strategies, as well as pipelines relative performance across datasets.

### Total sparsity

**Fig. 2:**
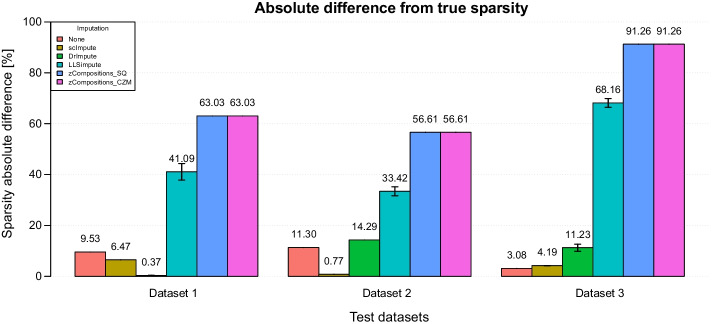
Absolute difference between sparsity in the ground truth and in the different pipelines for the three simulated datasets. (The lower the better). Results are aggregated according to the imputation method used in the pipelines and are reported as mean values calculated across the six pipelines using the given zero-imputation tool; error bars represent the corresponding standard deviations. “None” identifies pipelines where no zero-imputation step was performed, i.e. normalization-only pipelines

Table 4 reports the mean and standard deviation of the sparsity levels reconstructed by different pipelines, while Fig. 2 shows the differences between sparsity in the ground truth and in the imputed datasets. Results for each preprocessing pipeline are shown in Additional file 1: Tables S1-S3, where we report the percentage of null values of each preprocessed, raw and ground truth matrix.

**Table 4:** Count matrix sparsity for the three simulated datasets

Imputation	Dataset 1 True sparsity: 63.03% Raw data sparsity: 72.56%		Dataset 2 True sparsity: 56.61% Raw data sparsity: 67.91%		Dataset 3 True sparsity: 91.26% Raw data sparsity: 94.34%	
	Mean (%)	SD (%)	Mean (%)	SD (%)	Mean (%)	SD (%)
None	72.56	0.00	67.91	0.00	94.34	0.00
scImpute	69.50	0.01	55.84	0.00	87.07	0.01
DrImpute	62.67	0.04	42.32	0.00	80.03	1.42
LLSimpute	21.94	3.25	23.19	1.75	23.10	1.71
zCompositions_SQ	0.00	0.00	0.00	0.00	0.00	0.00
zCompositions_CZM	0.00	0.00	0.00	0.00	0.00	0.00

Preprocessed datasets sparsity were aggregated according to the zero-imputation method included in the pipeline, reporting the mean and standard deviation calculated across different normalization approaches. Ground truth and raw data sparsity for each dataset are reported in the table header row. “None” identifies pipelines where no zero-imputation step was performed, i.e. normalization-only pipelines

Pipelines using scImpute and DrImpute recreated true sparsity, only slightly overestimating or underestimating the true zero counts, depending on the tested dataset. In dataset 1 DrImpute pipelines were overall the best-performing preprocessing approaches, with an estimated sparsity range of 62.65–62.74% across different normalization approaches (Additional file 1: Table S1), followed by scImpute pipelines that slightly overestimated the total sparsity (69.50–69.51%). On the opposite, in simulated datasets 2 and 3 (Additional file 1: Tables S2-S3) scImpute overperformed DrImpute pipelines.

Pipelines using LLSimpute and zCompositions, with both SQ and CZM priors, heavily underestimated data sparsity in all the simulated datasets, recovering the majority (LLSimpute) or also the totality (zCompositions) of zero counts. In simulated dataset 1, for example, the true sparsity level was 63.03% but LLSimpute preprocessed datasets showed a percentage of zeros between 19.56 and 27%, depending on the normalization method used before zero-imputation (Additional file 1: Table S1), while zCompositions-treated dataset contained 0% of zero values. zCompositions over-imputation behaviour was expected to give poor performance on metrics that consider the number of imputed entries, but we included it for sake of completeness.

Looking only at this goodness measure, i.e. sparsity recovery ability, normalization seemed to have little or no effect on the efficiency of the pipeline, since the results of each pipeline using zero-imputation step were varying very weakly when choosing any of the normalization methods, or using no normalization at all (Additional file 1: Tables S1-S3). Obviously, normalization-only pipelines inherently did not act on sparsity, thus returning sparsity equal to the raw matrix level.

### Species presence/absence

**Fig. 3:**
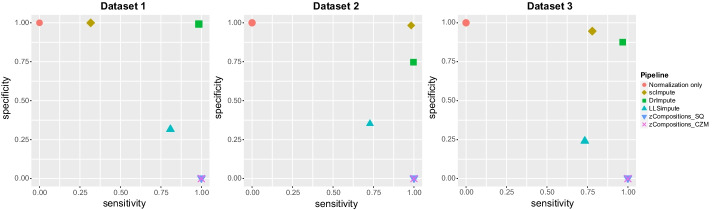
Specificity and sensitivity achieved by the different pipelines for the three simulated datasets. (The higher the better). Results are aggregated according to the imputation method used in the pipelines and are reported as mean values calculated among pipelines using the s zero-imputation tool. “Normalization only” identifies pipelines where no zero-imputation step was performed, i.e. normalization-only pipelines

The ability of the different pipelines to distinguish biological and technical zeros is illustrated in terms of specificity and sensitivity in Fig. 3 and Additional file 1: Tables S4-S6, which reports the mean sensitivity and specificity levels achieved by each imputation method, calculated across the different pipelines using it. Results for each preprocessing pipeline are shown in Additional file 1: Table S7. For a given imputation method, sensitivity and specificity values showed very low variability across pipelines (standard deviations are reported in Additional file 1: Tables S4-S6), thus confirming the almost negligible role of normalization choice prior to imputation.

scImpute always achieved the highest specificity among all the imputation pipelines, being also very close to the specificity of normalization-only/raw data, indicating that the tool is doing a particularly good job in not imputing true biological zeros. In terms of imputing technical zeros (sensitivity), scImpute achieved an extremely high sensitivity in dataset 2 and 3, while its performance on dataset 1 are modest. Similarly to scImpute, DrImpute always achieved a very high sensitivity on all the datasets, while maintaining a variable but high specificity (always > 75%) in all datasets. LLSimpute achieved a good sensitivity but a poor specificity on all the three datasets, in line with the heavy imputation detected in the previous section. zCompositions pipelines achieved a very high sensitivity and a very low specificity due to the imputation of the totality of zero values, thus not differentiating among biological and technical zeros. As for total sparsity metrics, pipelines based only on normalization did not recover any information about undetected species, and so they achieved the same sensitivity and specificity values of using raw data.

### Relative abundance profile

The distributions of SMAPE and Aitchison distance values across different samples are shown in Additional file 1: Figs. S1-S3 and Figs. S4-S6. In addition, Additional file 1: Tables S8-S9 show the p-values of one-sided paired Mann–Whitney U-test (Benjamini–Hochberg correction, significant threshold 0.05) and the corresponding effect sizes when comparing SMAPE and Aitchison distance values of preprocessed vs. raw data.

With few exceptions, the most evident differences in terms of SMAPE and Aitchison distance values are driven by the choice of the imputation method. Additional file 1: Tables S10-S12 show the median SMAPE and Aitchison distance across samples in the dataset achieved by each pipeline. Median SMAPE and Aitchison distance values are further summarized in Table 5, reporting their mean and standard deviation across pipelines using the same imputation method. As for the total sparsity metric, normalization-only preprocessing pipelines inherently did not affect the results, resulting in the same SMAPE and Aitchison distances achieved by raw data.

**Table 5:** SMAPE and Aitchison distance between the ground truth and different pipelines for the three simulated scenarios

Dataset 1				Dataset 2				Dataset 3			
SMAPE (Raw: 12.109)		Aitchison distance (Raw: 19.039)		SMAPE (Raw: 15.493)		Aitchison distance (Raw: 23.754)		SMAPE (Raw: 2.717)		Aitchison distance (Raw: 4.972)	
Pipeline	Mean (SD)	Pipeline	Mean (SD)	Pipeline	Mean (SD)	Pipeline	Mean (SD)	Pipeline	Mean (SD)	Pipeline	Mean (SD)
DrImpute**	7.648 (0.263)	DrImpute*	18.683 (0.170)	scImpute**	12.059 (0.007)	zCompositions_SQ*	23.354 (0.192)	None	2.717 (0)	None	4.972 (0)
scImpute**	11.193 (0.001)	None	19.039 (0)	None	15.493 (0)	None	23.754 (0)	scImpute	6.188 (0.034)	zCompositions_CZM	7.919 (2.384)
None	12.109 (0)	zCompositions_SQ*	19.141 (0.041)	DrImpute	28.437 (2.566)	DrImpute*	24.044 (2.384)	DrImpute	15.187 (1.555)	zCompositions_SQ	12.498 (1.880)
zCompositions_CZM	73.427 (0.674)	zCompositions_CZM*	20.614 (1.549)	zCompositions_CZM	70.274 (1.563)	scImpute	25.112 (0.001)	LLSimpute	78.645 (1.215)	DrImpute	19.280 (3.263)
zCompositions_SQ	77.280 (0.045)	scImpute	26.408 (0.001)	zCompositions_SQ	71.647 (0.594)	zCompositions_CZM*	28.250 (4.941)	zCompositions_CZM	95.605 (0.135)	scImpute	25.240 (0.001)
LLSimpute	77.717 (3.246)	LLSimpute	105.394 (8.463)	LLSimpute	80.013 (2.001)	LLSimpute	81.979 (0.702)	zCompositions_SQ	95.614 (0.061)	LLSimpute	56.107 (3.932)

For each metric and dataset, results are ordered according to decreasing performance (the lower the better). Raw data results are reported in the second row of the table header. The table shows the mean and standard deviation calculated across the pipelines using a specific zero-imputation tool of the median SMAPE and Aitchison distance calculated across the samples in each dataset. “None” identifies pipelines where no zero-imputation step was performed, i.e. normalization-only pipelines. Imputation pipelines that always achieve a statistically significant improvement (i.e. a lower metric compared to raw data) are indicated with “**”. Imputation pipelines that achieve a statistically significant improvement only in some of the associated pipelines (i.e. only when combined with some normalization methods) are indicated with “*”

This analysis allowed us to highlight how in dataset 1 DrImpute performed very well not only in recognizing which features got unobserved in the sequencing process (see the previous section), but also in correctly retrieving the information on relative count distribution within each feature. DrImpute pipelines perform better than normalization-only pipelines and raw data, achieving a SMAPE between 7.4% and 8% (Additional file 1: Table S10) across the normalization pairing methods and an Aitchison distance in the range 18.4–18.7 (Additional file 1: Table S10). As for the sparsity metric, DrImpute pipelines show a drop in performance when applied to the other two simulated datasets, performing generally worse than raw and normalized-only data, however still maintaining a good performance compared to other imputation pipelines.

Also scImpute achieved very good performance, showing SMAPE among the lowest on all the test datasets and performing better than raw and normalized-only data in simulated dataset 1 and 2 (Additional file 1: Figs. S1-S2, Table S8). In contrast, if we consider Aitchison distance, scImpute pipelines do not bring any improvement compared to raw and normalized-only data.

Pipelines containing LLSimpute performed always poorly in terms of SMAPE and Aitchison distance in all the three simulation scenarios and independently of the chosen normalization step. Interestingly, zCompositions, in both the considered variants, achieved poor performance in terms of SMAPE but good performance in terms of Aitchison distances, which are among the lowest across datasets and pipelines, so reflecting the ability of the tool in considering the compositional properties of the data. Indeed, DrImpute and zCompositions, in combination with some normalization approaches, are the only methods able to achieve a (statistically significant) lower Aitchison distance compared with normalized-only or raw data in datasets 1 and 2.

Normalization-only pipelines always performed better than LLSimpute, zCompositions_SQ and zCompositions_CZM in terms of SMAPE. They also achieved a lower Aitchison distance compared to LLSimpute. A special case was observed for simulated dataset 3, where all the pipelines involving zero imputation resulted in some bias introduction when dealing with null values. In this scenario, raw and normalized-only data resulted to be the most adherent in terms of relative proportions to the real one, with a SMAPE of 2.72% and an Aitchison distance of 4.97 (Table 5 and Additional file 1: Table S12).

The possible discrepancy between SMAPE and Aitchison distance results can be explained by highlighting that the two evaluation metrics are differently sensitive to errors at low abundance levels. Indeed, SMAPE is a relative measure of error calculated for each matrix row (ASV/OUT), while Aitchison values reflect a distance between (bacterial community) vectors. This inherently means that SMAPE is more sensitive to a high number of small absolute errors in count reconstruction than Aitchison distance and, consequently, that a low total discrepancy (Aitchison distance) in reconstructing ground truth counts could be reflected in a high median relative error (SMAPE) if the errors are mainly linked to low abundance features. Conversely, a tool with an excellent ability in recovering true relative abundances in the majority of features that makes some sporadic errors of remarkable entity (and maybe in one/some highly abundant feature/s) will result in a low mean SMAPE per sample (and, consequently, a median overall SMAPE per dataset) and a high Aitchison distance value.

### Impact on bacterial diversity

#### Alpha diversity

As previously introduced, alpha diversity indices permit to have an overview of sample composition, in terms of the number of detected species (Richness indices) and the distribution of counts within them (Evenness indices). To identify the ability of pipelines in achieving alpha diversity values close to the ones observed in ground truth data, we measured the relative error between alpha diversity values computed on ground truth data and the ones obtained from preprocessed data.

**Fig. 4:**
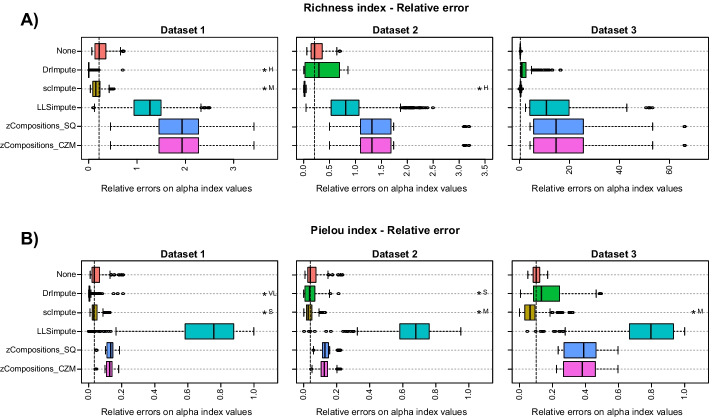
Relative error on Richness (**A**) and Pielou (**B**) alpha diversity indices for the three simulated datasets. (The lower the better). Relative errors are computed between alpha indices value of ground truth data and preprocessed data are shown. Results are aggregated according to the imputation method used in the pipelines. “None” identifies pipelines where no imputation is performed (i.e. raw and normalized-only data). Distributions of relative error values that result statistically lower (one-sided paired Mann–Whitney paired U-test, Benjamini–Hochberg correction, significant threshold 0.05) than relative errors calculated on raw/normalized-only data are indicated with the symbol “*”, followed by the interpretation of Cohen’s d effect size (N: negligible, VS: very small, S: small, M: medium, L: large, VL: very large, H: huge). The vertical dashed line indicates the median relative error on raw/normalized-only data

Figure 4A, Additional file 1: Figs. S7-S9 and Table S13 show that scImpute pipelines always achieved Richness indices very close to the real ones (low relative error), performing comparable or better than normalized-only and raw data in dataset 1 and 2. DrImpute pipelines performed significantly better than normalized-only and raw data in simulated dataset 1, while they showed a drop in performance on dataset 2 and 3. None of the other imputation pipelines were able to provide better Richness values.

Looking at Pielou index (Fig. 4B, Additional file 1: Figs. S10-S12 and Table S14), scImpute pipelines always achieved Pielou indices closer to the real ones compared with raw data and normalization-only pipelines. DrImpute pipelines overperformed raw and normalized-only data in simulated dataset 1 and 2, whereas they show a drop in performance compared to normalization-only strategies on dataset 3. As for Richness indices, none of the other imputation pipelines were able to provide better Pielou values compared to using raw data or normalized-only data.

**Fig. 5:**
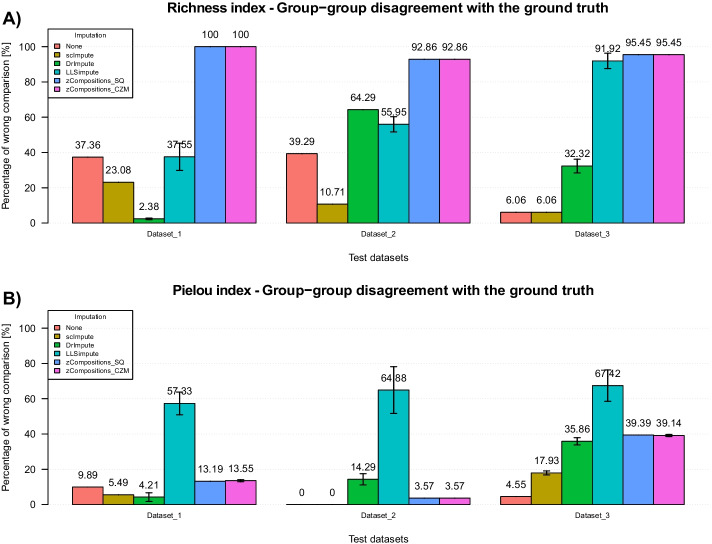
Results on Richness (**A**) and Pielou (**B**) alpha diversity indices for the three simulated datasets in terms of percentage of group–group comparisons disagreeing with the ground truth. (The lower the better). Results are aggregated according to the imputation method used in the pipelines. “None” identifies pipelines where no imputation is performed (i.e. raw and normalized-only data)

Alpha diversity values are often used to detect differences in composition between groups within an experiment by means of statistical tests comparing alpha values of samples in different groups. Results obtained from Mann–Whitney tests (reported in Fig. 5A and Additional file 1: Tables S15-S17) show how high performance in rare species recovery are reflected in a low number of incorrect (in relation to the ground truth) group–group comparisons. Indeed, it is evident the effect of scImpute pipelines application in improving richness comparisons results in all the three test scenarios compared with raw or normalized-only data. Also DrImpute pipelines and some LLSimpute pipelines achieved good performance in dataset 1, but they had a drop in performance in the other two datasets. Interestingly, the bad results in terms of single Richness index values achieved by LLSimpute on dataset 1 (Fig. 4A and Additional file 1: Fig. S7) had a small impact on group–group comparisons (Fig. 5A and Additional file 1: Table S15-S17). Indeed, group–group comparison tests differences in alpha indices values distributions, and so it is less sensitive to errors on single index values.

Regarding group–group comparison through alpha diversity measured as evenness (Fig. 5B and Additional file 1: Tables S18-S20), scImpute pipelines performed better than raw and normalization-only data on dataset 1 and it achieved comparable performance in dataset 2. DrImpute pipelines overperformed raw data and normalization-only pipelines in dataset 1, whereas zCompositions pipelines achieved slightly worse performance compared with raw and normalized-only data on dataset 1 and 2. Using the Pielou index, the worst performance on group–group comparison was obtained in all tested dataset by LLSimpute, both applied singularly and preceded by a normalization step. None of the imputation pipelines improved raw data and normalization-only results in dataset 3.

#### Beta diversity

As previously introduced, beta diversity indices are used to measure dissimilarity between samples. Bray–Curtis dissimilarity and Whittaker index were calculated to show different aspects of the considered matrix. Bray–Curtis dissimilarity was used to build a distance matrix on which Non-metric Multidimensional Scaling (NMDS) dimensionality reduction was performed to assess spatial distribution of samples, whereas Whittaker dissimilarity values were graphically represented using heatmaps. Due to their size, the graphical outputs resuming these results are available in Supplementary Materials (Additional file 1: Figs. S13-S18).

Additional file 1: Figs. S13-S15 show the results obtained using NMDS dimensionality reduction on Bray–Curtis distance performed on the three simulated datasets. In simulation 1 (Additional file 1: Fig. S13) and simulation 2 (Additional file 1: Fig. S14), real dataset samples belonging to the same group (biological replicates) tended to be very close to each other in the two dimensional NMDS subspace. This characteristic got lost after the sequencing process, i.e. in raw data, where samples of the same group tended to move away from each other and to form greater spatial clusters with members of other groups. DrImpute and scImpute pipelines including the normalization step were the best performing strategies to recover the original structure of the data, being able to get samples belonging to the same experimental condition closer to each other and divide different groups. Normalization-only pipelines were unable to recover the original structure of the data, showing also very little difference in the samples spatial disposition compared with raw data.

In simulation 3 (Additional file 1: Fig. S15), very little information got lost during the sequencing process. This reflected on NMDS results on Bray–Curtis distance, from which we can see that real, raw and normalized data are very similar to each other. This dataset was included in this study with the principal aim of assessing the possible bias introduction of zero-imputation pipelines in experiments where no or little sequencing zeros are present. NMDS plots showed that imputation pipelines performed comparable (scImpute and DrImpute) or worse (LLSimpute and zCompositions) than normalization-only pipelines. DrImpute pipelines caused an artificial over-separation of groups, while scImpute tended to reduce intra-group samples variability. Pipelines involving LLSimpute and zCompositions generally brought additional noise, favouring the scattering of observations on the plain and consequently leading to group information loss on all the test datasets.

Beta indexes were used to calculate distances based on the number of shared and exclusive features between samples. As the Whittaker information is less rich than the abundance-based beta metric, the obtained distance matrices were represented as heatmaps in which a colour scale links the shade to a distance value (Additional file 1: Figs. S16-S18). The results based on this metric resemble the ones obtained considering abundance data, suggesting that sample grouping is mainly based on richness information, i.e. on the presence/absence of features.

scImpute pipelines were found to be the most effective in recreating real sample distances in terms of Whittaker index, being able to achieve a very good information recovery in terms of intra-group and inter-group distances (intra-group distances were slightly underestimated only in dataset 3). Using DrImpute pipelines, sometimes samples within a group tended to have all the same distance with respect to the samples of other groups. This behaviour is clearly visible in the second, third, fourth and fifth groups in dataset 2 (Additional file 1: Fig. S17), with the consequent creation of a unique, big group including them all. A similar behaviour was also observed for dataset 3 (Additional file 1: Fig. S18). As observed for Bray–Curtis dissimilarity, results on Whittaker index showed that normalization-only pipelines tended to overestimate intra-group diversity in all datasets, while pipelines involving LLSimpute and zCompositions were not able to recreate accurate samples distances.

### Differential abundance analysis

A test for differential abundance analysis results was performed as a measure of the ability of each pipeline to reconstruct the original data characteristics. The result of this investigation is summarized in Additional file 1: Figs. S19-S21 as box plots of Jaccard indices computed between species identified as differentially abundant (DA) in preprocessed and ground truth data. The median Jaccard index obtained using raw and ground truth data is indicated by a dashed vertical line. The labels present on the right of each box plot are related to the significance of the Mann Whitney u test performed to test for improvement with respect to raw data results and, in case of significant difference (*p* < 0.05, Benjamini–Hochberg FDR correction), to the magnitude of the size effect. Corrected p-values and effect size values are reported in Additional file 1: Table S21.

scImpute pipelines significantly improved DA analysis compared with using raw data, also achieving larger Jaccard indices and effect sizes than normalization only-pipelines on all the test datasets. In particular, scImpute are the best performing pipelines on dataset 2 and 3 (effect size “large” and “huge”), and the second best in dataset 1 (effect size “medium” and “large”). DrImpute pipelines achieved larger Jaccard indices compared with raw and normalized-only data in dataset 1 (best performing pipelines, effect size “very large” and “huge”) and dataset 3 (effect size “huge”), whereas they performed worse than raw/normalized-only data in dataset 2. None of the other imputation pipelines performed better than raw data, except TSS + zComposition_SQ in dataset 1 and 2 (Additional file 1: Figs. S19-S20, Table S21) and TSS + LLSimpute in dataset 3 (Additional file 1: Fig. S21, Table S21). Normalization-only pipelines led to a general improvement in retrieving differential abundant features compared to the performance on raw data in dataset 1 and 2 (Additional file 1: Figs. S19-S20, Table S21), but the corresponding effect sizes were all "Very Small" or "Small". Unlike other metrics, the choice of the normalization method, both alone and prior to zero-imputation, had a larger impact on pipeline performance, still remaining less influential than the choice of zero-imputation methods.

### Final considerations

Summarizing, scImpute pipelines turned out to be the overall best choice for 16S rDNA-Seq data zero imputation among the tested approaches. Indeed, despite some slight oscillation in performance between the adopted datasets, it achieved optimal results in terms of recovering rare species and detecting differential abundances and very satisfying performance in alpha and beta diversity reconstruction. It is noteworthy that, in some cases (e.g. differential abundance analysis, Additional file 1: Fig. S19 and Table S21), normalizing data before imputing zero values slightly improves performance, even though raw data are required in input by the tool specification. DrImpute showed optimal results in simulation 1 for most metrics, but it seemed more sensitive than scImpute to data characteristics, obtaining generally good but variable performance on the other simulated datasets. Regarding zCompositions, some differences in performance were obtained when considering its two modalities (SQ and CZM). Indeed, even though they both left no zero values after imputation, thus giving SMAPE and richness poor performance, the more refined SQ zero-imputation gave better results in terms of overall abundance profile reconstruction and differential analysis, compared with CZM mode. Despite these differences, results on the great majority of metrics and simulated datasets were not good for zCompositions. The overall worst performance was obtained by LLSimpute, which obtained poor results in almost all the considered metrics and simulations.

Results regarding the pipelines involving only the normalization step showed how this state-of-the-art preprocessing can slightly ameliorate downstream analyses (e.g. differential abundance analysis) but cannot inherently correct the excess of sparsity or influence those analyses that are constitutionally sensitive to presence/absence information (e.g., richness or beta diversity based on binary data). As a consequence, different normalization tools achieved similar performance in the same scenario, being often comparable with results obtained using raw data. However, looking at the differential abundance analysis we can see how the performance of normalization methods varies when passing from a simulated dataset to another. This observation is in concordance with the performance variations previously observed by Weiss et al. [6], that concluded that normalization effects depend upon data characteristics.

Finally, it was evident from all the evaluation metrics how zero-imputation had a preeminent effect on data analysis and, consequently, how influential is the choice of the tool to perform this step.

**Fig. 6:**
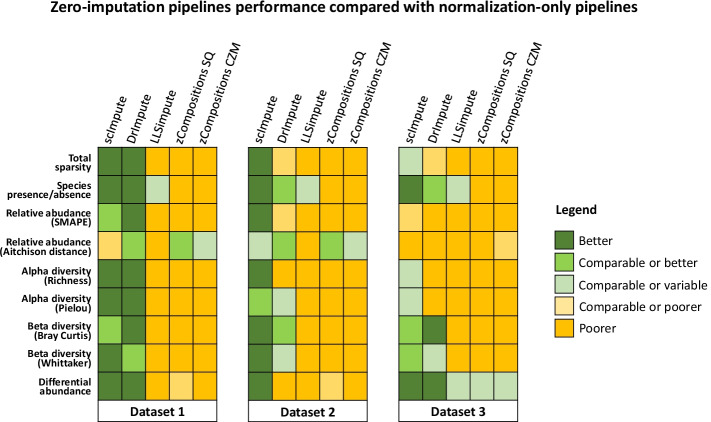
Qualitative summary of overall imputation pipelines performance compared with raw/normalized-only pipelines. Colours were assigned by qualitatively balancing the goodness of each pipeline among all the evaluation metrics. Results are aggregated according to tested imputation methods. Performance classified as “Comparable or variable” describe pipelines achieving performance that are comparable to the ones obtained by normalization-only pipelines (or raw data) or for which there is not a clear trend in the performance, i.e. sometime they perform better than normalization-only, other times they perform worse

An overall qualitative summary of performance for the tested pipelines and the adopted metrics is reported in Fig. 6.

## Discussion

We have presented a comparative assessment of 35 different 16S preprocessing pipelines, including current approaches to 16S data preprocessing based on normalization methods, testing a novel step of data recovery through zero-imputation, and combining normalization and zero-imputation strategies.

Normalization methods were selected among tools specifically designed for microbial data analysis (CSS and GMPR) and approaches developed for the analysis of other classes of sequencing data (TSS, edgeR and DESeq2). The included normalization methods represented the most widely used tools for 16S data normalization and so they provided a good picture of the state-of-the-art 16S data preprocessing.

Since no 16S rDNA-Seq zero-imputation method was available in literature and there was no evidence about which class of zero-imputation methods may be suitable for metataxomic data, we selected methods from different fields. The similarities between 16S rDNA-Seq data and scRNA-seq data led us to include two of the most used scRNA-seq zero-imputation tools, such as DrImpute and scImpute. Similarly, we selected zero-imputation tools for microarray RNA expression data such as LLSimpute, being aware that microarray data had different technical characteristics from sequencing data. Last, we included zero-imputation tools specifically designed for compositional data such as zCompositions, leveraging on the knowledge that 16S rDNA-Seq had a compositional nature. zCompositions was tested with two alternative configurations, briefly named "CZM" and "SQ". Despite being very interesting and well-structured imputation strategies, the solutions proposed in the GBM configuration of zCompositions tool had to be excluded from the benchmark because not easily extendable to rDNA-seq data analysis (see Methods). Similarly, another tool currently adopted for imputation of compositional data, namely robCompositions [58], was not considered in the comparison. robComposition implements the methods introduced by Hron, Templ and Filzmoser in their work [59], where they propose two different imputation algorithms for estimating missing values in compositional data: a k-nearest neighbour (knn) imputation and an iterative model-based imputation. Since the knn imputation implementation showed not to manage situations in which too many features with null values are present in the analysed dataset, another very common situation in 16S rDNA-Seq data analysis, we excluded the use of knn imputed values to initialize the iterative algorithm, that was consequently also excluded. We also tried an alternative solution based on the "roundedZero" option, which consists in performing a zero-substitution with 0.001 to initialize the iterative procedure. This led to no solution, because the high percentage of null values still represented a problem for regression methods that did not converge to any solutions after hours of running time.

All the pipelines included in this study were tested on three scenarios characterized by a different number of samples, number of groups, sequencing depths and sparsity. In this working framework, it was of central importance to identify a ground truth reference with which the output of the tested tools could be compared in order to evaluate their performance according to a set of predefined metrics or characteristics. In this investigation, the theoretical ground truth for performance comparison would be the real, unobservable abundance table describing the community composition before data production. In silico data have the characteristic of providing access to (simulated) community composition, being produced in a controlled standardized procedure with known characteristics, thus being a suitable object on which to perform tools assessment. On the contrary, experimental 16S count data could not serve this scope as they are just an approximated measure of the underlying but not accessible true bacterial abundances (i.e. the ground truth). Please note that this is true for any kind of sequencing count data, as proved by the large use of simulated count data in the assessment of bioinformatics preprocessing and analysis methods [6, 13, 20, 60–67]. Moreover, although we initially considered the use of mock communities as touchstones to verify the loss information recovery, the limited number of bacterial components characterising them would have been a dramatic simplification of the real analysis frameworks, in which hundreds or thousands of features are present within a bacterial community. As a further alternative, we also considered to use data in which both 16S and shotgun sequencing were available. However, also this option was discarded for this benchmark as relative abundances produced by the two techniques suffer from a discrepancy inherited by the different principles underlying the two experimental data production protocols.

On the other hand, using simulated data poses the question of how much the synthetic datasets match the characteristics of real datasets. We chose the metaSPARSim simulator since it has been proved to create synthetic datasets that resemble the intensity, variability and sparsity observed in real OTU/ASV table [21]. In particular, metaSPARSim data were demonstrated to accurately resemble OTU sparsity, sample sparsity and OTU intensity-sparsity relation, and so provide robust simulated data for the assessment of zero-imputation methods.

Among the characteristics not modeled in metaSPARSim synthetic data there is the taxonomic classification of the simulated OTUs. Indeed, no phylogenetic information is included in the tool while simulating data, so obtained OTUs do not carry phylogenetic links information. Similarly, metaSPARSim simulated abundances were simulated by not considering the possible bacterial interaction structure. However, the lack of these two characteristics did not affect the applicability of metaSPARSim data to the context of zero-imputation methods assessment, since none of the tested imputation methods involves nor taxonomic information or biological interaction networks that could characterize the OTU/ASV table.

For the benchmarking procedure, a scenario-driven approach was chosen and three different synthetic datasets were used in order to mimic three different realistic data contexts: an animal gut microbiota survey characterised by average sparsity level and low variability among replicates; a human microbiota study, with higher variability among replicates, but limited count matrix sparsity; a food microbiota experiment characterised by high sequencing depth, high sparsity and average variability.

The pipelines including zCompositions resulted to be good in preserving overall proportional distribution in terms of Aitchison distance from ground truth, reflecting the ability of the tool in considering the compositional properties of the data. On the other hand, zCompositions performed poorly in sparsity recovery metrics (i.e. total sparsity, species presence/absence and alpha-diversity with richness index) since it was designed for compositional data assuming that all or most of the zeros are missing values and not true zeros.

LLSimpute was found to be not adequate for 16S rDNA-Seq data preprocessing in all the tested datasets and according to all the selected metrics, thus demonstrating the non-transferability of these techniques into 16S microbiome studies framework.

The most performing, reliable, and robust pipelines were the ones that included scImpute and DrImpute tools, combined with any of the normalization methods. These pipelines showed very good performance in retrieving truly present species, reconstructing proportional abundance levels and improving the accuracy of downstream analyses. They both had a drop in performances in some cases, especially in the challenging scenario presented in simulated dataset 3 where no or little sequencing zeros are present. However, these two tools performed comparable or better than normalization-only pipelines in the majority of metrics and test datasets, thus representing valid options for 16S rDNA-Seq count data preprocessing.

Overall, the introduction of zero-imputation step using methods designed for sparse sequencing data allowed recovering the lost information very well, while controlling the introduction of possible unwanted biases. In our tests, the normalization step showed often a small improvement of zero-imputation performances compared to zero-imputation-only pipelines, improvement observed also for tools that explicitly ask for non-normalized counts, such as scImpute. However, the improvement was of similar entity when applying different normalization tools, thus suggesting that zero-imputation tools are not very sensitive to different choices of the normalization method.

Consistently with results from scRNA-seq field, the number of samples in each experimental condition may affect the performance of the different preprocessing tools, but preliminary tests suggested that best/worst performing methods and relative ranking of tools performance are preserved (data not shown).

Overall, the present study has been designed with the idea of testing for the meaningfulness of a possible future scientific effort in a new context, i.e. zero-imputation applied to 16S rDNA-Seq data, that had saw no attention till now. As a consequence, some limiting aspects may be seen in this seminal work.

First, a possible limitation of this work is that, despite the large number of available zero-imputation approaches proposed in different research fields, especially for scRNA-seq data analysis [13, 14], we included a limited number of imputation methods. Indeed, the main goal of this study was the assessment of whether zero-imputation improves 16S rDNA-Seq data preprocessing, and the identification, if any, of pipelines that can improve typical 16S data preprocessing approaches. In particular, we excluded the use of deep learning based methods since the number of samples typically available in 16S studies is quite limited. Second, we used the different normalization and imputation methods following the tools guidelines when a minimal parameter tuning was required, i.e. mimicking the average user approach, with no extensive parameter tuning performed for the tool performance optimization. On the other hand, the use of three different scenarios for the benchmarking datasets allow testing different methods in quite different situations, thus indirectly assessing whether the default/suggested parameters offer or not a sufficiently robust approach under different experimental conditions. Third, although some tools are available in literature to perform differential abundance statistical analysis, in our study we chose to perform it using a non-parametric Mann–Whitney U test. The existence of specific methods for this testing procedure suggests that Mann–Whitney test may not be considered as the optimal one. However, this choice was done because each differential abundance approach presents its own underlying model and assumptions, and this is crucial for the potential biases introduced by different DA tools. Consequently, we decided for the adoption of a possible non-optimum, but less biased approach.

## Conclusion

Population-wide microbial surveys became possible in the last decades thanks to the advent of NGS platforms, i.e. technologies that allow for deep, high-throughput, in-parallel DNA sequencing. Due to its high amount of information at ever lowering time and cost expense, 16S rDNA-Seq allows large, longitudinal, culture-free microbiome studies that permit a deep characterization of the investigated niches. This advantageous benefit/cost ratio guaranteed this approach an advantage over other methodologies (such as shotgun metagenomics), making it the most frequently adopted approach to microbiota studies. However, the appropriate treatment of the produced data is still a very challenging issue, because of data peculiar characteristics, such as extreme sparsity.

In this study, a first assessment on the influence of introducing the zero-imputation step in 16S rDNA sequencing count data preprocessing has been performed. The idea to include a zero-imputation step derived from the wide application of such methodologies on other types of sparse sequencing count data such as single cell RNA-Seq, and from the proved benefit of including such preprocessing step in the accuracy of several bioinformatics analyses.

Here we performed a benchmarking of 16S rDNA-Seq data preprocessing tools, including both normalization-only pipelines—that reflected currently adopted 16S data treatment workflow—and pipelines with a zero-imputation step, for a total of 35 preprocessing pipelines.

This work was performed following the downstream analyses typically present in microbiome studies based on 16S rDNA-Seq data, studying the impact of the chosen preprocessing pipeline in terms of consequent changes in conclusions from the ones obtained using the ground truth data. More precisely, differences in sparsity, species presence/absence, sample proportional abundance distributions, alpha and beta diversity indices and differential analyses were assessed.

The results suggest that introducing a properly-performed zero-imputation step in the preprocessing of 16S rDNA-Seq data improves data sparsity and the relative sample abundance estimation, with positive effects on downstream analyses, such as the computation of alpha and beta diversity indices and differential abundance analysis. For many analyses and datasets, the choice of the zero-imputation method showed a more important role compared to the normalization step. Moreover, it has been possible to identify the best performing normalization and imputation tools and their optimal combination in order to help researchers to maximize the robustness and accuracy of the results and conclusions when performing their microbiota studies. The findings of the present study have already been successfully used in a recently-published paper [68], where the GMPR + scImpute pipeline was chosen among others to preprocess 16S rDNA-Seq data count data, obtaining meaningful results in highlighting a consistent biological information carried by the data.

Although the results of our study clearly showed a promising way for further improving the reliability and quality of metataxonomic analyses, we think that our work just laid the first foundations for a future, deepest research, and that far more effort should be done by the scientific community along this way to better identify tools and pipelines to efficiently include the zero-imputation step within the 16S rDNA-Seq framework. This would mean not only further benchmarking on tools that could be transferred from others contexts to the metataxonomic one, but also the implementation and testing of specific tools for 16S rDNA-Seq count data. 16S rRNA gene protocols are likely to change in time as new technologies develop. Therefore, identifying the best preprocessing methods is an open problem. This paper is a first step towards the development of an open source and collaborative framework for the assessment of zero imputation methods in the context of 16S rDNA-seq data able to continuously evaluate current and new imputation strategies, as well as to identify robust preprocessing pipelines to be used in the analysis of metataxonomic data.

### Supplementary Information

**Additional file 1**. Supplementary material, including Supplementary Figures and Tables.

## Data Availability

Simulated data (ground truth and raw data), preprocessed data and scripts to reproduce the findings of this work are included in a R package available on a public git repository at https://gitlab.com/sysbiobig/beware-to-ignore-the-rare-imputation-16s. A Docker container image containing the data, the developed R package, the normalization and zero-imputation tools used in this study, and all the required dependencies is available at the same link.

## Abbreviations

ASV: Amplicon sequence variant
CPM: Count Per Million
CSS: Cumulative Sum Scaling
DESeq: Differential expression analysis for sequence count data
FDR: False discovery rate
FN: False negative
FP: False positive
GMPR: Geometric mean of pairwise ratios
HMP: Human microbiome project
HTS: High-throughput sequencing
NGS: Next generation sequencing
NMDS: Non-metric multidimensional scaling
OTU: Operational taxonomic unit
rDNA-Seq: Ribosomial DNA sequencing
rRNA: Ribosomial RNA
SMAPE: Symmetric Mean Absolute Percentage Error
TMM: Trimmed mean of M-values
TN: True negative
TP: True positive
TSS: Total sum scaling
