# Supplementary material for: Beware to ignore the rare: how imputing zero-values can improve the quality of 16S rRNA gene studies results
Source: BMC Bioinformatics. 2022 Feb 7;22(Suppl 15):618. doi: 10.1186/s12859-022-04587-0 (PMC8822630; doi:10.1186/s12859-022-04587-0)
Supplement: Supplementary file 1 — Additional file 1. Supplementary material, including Supplementary Figures and Tables. [file 12859_2022_4587_MOESM1_ESM.pdf]

Beware to ignore the rare: how imputing zero-values can improve the quality of 16S rRNA gene studies results

Giacomo Baruzzo, Ilaria Patuzzi, Barbara Di Camillo

Supplementary materials

Detailed results obtained for each analysed pre-processing pipeline.

Total sparsity.

Table S1. Simulated Dataset 1, count matrix sparsity. Real, raw and pre-processed datasets are ordered for decreasing similarity with true sparsity (i.e. sparsity of real data). In the "Behaviour" column, datasets with overestimated sparsity are labelled with "O", while underestimation is labelled with "U".

Pipeline	Sparsity	Behaviour
Real	63.03 %	-
None_DrImpute	62.74 %	U
TSS_DrImpute	62.65 %	U
CSS_DrImpute	62.65 %	U
edgeR_DrImpute	62.65 %	U
DESeq2_DrImpute	62.65 %	U
GMPR_DrImpute	62.65 %	U
TSS_scImpute	69.5 %	O
edgeR_scImpute	69.5 %	O
DESeq2_scImpute	69.5 %	O
GMPR_scImpute	69.5 %	O
CSS_scImpute	69.51 %	O
None_scImpute	69.51 %	O
Raw	72.56 %	O
TSS	72.56 %	O
CSS	72.56 %	O
edgeR	72.56 %	O
DESeq2	72.56 %	O
GMPR	72.56 %	O
TSS_LLSimpute	27 %	U
None_LLSimpute	25.09 %	U
edgeR_LLSimpute	20.46 %	U
CSS_LLSimpute	19.93 %	U
DESeq2_LLSimpute	19.62 %	U
GMPR_LLSimpute	19.56 %	U
None_zCompositions_SQ	0 %	U
None_zCompositions_CZM	0 %	U
TSS_zCompositions_SQ	0 %	U
TSS_zCompositions_CZM	0 %	U
CSS_zCompositions_SQ	0 %	U
CSS_zCompositions_CZM	0 %	U
edgeR_zCompositions_SQ	0 %	U
edgeR_zCompositions_CZM	0 %	U
DESeq2_zCompositions_SQ	0 %	U
DESeq2_zCompositions_CZM	0 %	U
GMPR_zCompositions_SQ	0 %	U
GMPR_zCompositions_CZM	0 %	U

Table S2. Simulated Dataset 2, count matrix sparsity. Real, raw and pre-processed datasets are ordered for decreasing similarity with true sparsity (i.e. sparsity of real data). In the "Behaviour" column, datasets with overestimated sparsity are labelled with "O", while underestimation is labelled with "U".

Pipeline	Sparsity	Behaviour
Real	56.61 %	-
None_scImpute	55.85 %	U
GMPR_scImpute	55.84 %	U
DESeq2_scImpute	55.84 %	U
TSS_scImpute	55.84 %	U
CSS_scImpute	55.84 %	U
edgeR_scImpute	55.84 %	U
Raw	67.91 %	O
TSS	67.91 %	O
CSS	67.91 %	O
edgeR	67.91 %	O
DESeq2	67.91 %	O
GMPR	67.91 %	O
None_DrImpute	42.32 %	U
TSS_DrImpute	42.32 %	U
CSS_DrImpute	42.32 %	U
edgeR_DrImpute	42.32 %	U
DESeq2_DrImpute	42.32 %	U
GMPR_DrImpute	42.32 %	U
edgeR_LLSimpute	24.99 %	U
DESeq2_LLSimpute	24.19 %	U
CSS_LLSimpute	24.05 %	U
None_LLSimpute	23.42 %	U
TSS_LLSimpute	22.42 %	U
GMPR_LLSimpute	20.08 %	U
None_zCompositions_SQ	0 %	U
None_zCompositions_CZM	0 %	U
TSS_zCompositions_SQ	0 %	U
TSS_zCompositions_CZM	0 %	U
CSS_zCompositions_SQ	0 %	U
CSS_zCompositions_CZM	0 %	U
edgeR_zCompositions_SQ	0 %	U
edgeR_zCompositions_CZM	0 %	U
DESeq2_zCompositions_SQ	0 %	U
DESeq2_zCompositions_CZM	0 %	U
GMPR_zCompositions_SQ	0 %	U
GMPR_zCompositions_CZM	0 %	U

Table S3. Simulated Dataset 3, count matrix sparsity. Real, raw and pre-processed datasets are ordered for decreasing similarity with true sparsity (i.e. sparsity of real data). In the "Behaviour" column, datasets with overestimated sparsity are labelled with "O", while underestimation is labelled with "U".

Pipeline	Sparsity	Behaviour
Real	91.26 %	-
Raw	94.34 %	O
TSS	94.34 %	O
CSS	94.34 %	O
edgeR	94.34 %	O
DESeq2	94.34 %	O
GMPR	94.34 %	O
GMPR_scImpute	87.08 %	U
DESeq2_scImpute	87.08 %	U
None_scImpute	87.07 %	U
CSS_scImpute	87.06 %	U
TSS_scImpute	87.06 %	U
edgeR_scImpute	87.05 %	U
TSS_DrImpute	81.86 %	U
edgeR_DrImpute	81.85 %	U
CSS_DrImpute	79.28 %	U
DESeq2_DrImpute	79.07 %	U
GMPR_DrImpute	79.07 %	U
None_DrImpute	79.03 %	U
TSS_LLSimpute	25.57 %	U
None_LLSimpute	23.73 %	U
DESeq2_LLSimpute	23.56 %	U
CSS_LLSimpute	22.72 %	U
GMPR_LLSimpute	22.71 %	U
edgeR_LLSimpute	20.33 %	U
None_zCompositions_SQ	0 %	U
None_zCompositions_CZM	0 %	U
TSS_zCompositions_SQ	0 %	U
TSS_zCompositions_CZM	0 %	U
CSS_zCompositions_SQ	0 %	U
CSS_zCompositions_CZM	0 %	U
edgeR_zCompositions_SQ	0 %	U
edgeR_zCompositions_CZM	0 %	U
DESeq2_zCompositions_SQ	0 %	U
DESeq2_zCompositions_CZM	0 %	U
GMPR_zCompositions_SQ	0 %	U
GMPR_zCompositions_CZM	0 %	U

Species presence/absence

Table S4. Dataset 1 - Sensitivity and specificity on species presence/absence aggregated by imputation method. Sensitivity and specificity values in classifying species presence/absence are aggregated according to the imputation method used in the pipelines and are reported as mean and standard deviation (SD) values calculated among normalization methods.

	Sensitivity		Specificity	
**Imputation pipeline**	**mean**	**SD**	**mean**	**SD**
None (Normalization only)	0.000	0.000	1.000	0.000
DrImpute	0.984	0.006	0.992	0.000
scImpute	0.316	0.000	0.999	0.000
LLSimpute	0.809	0.047	0.319	0.045
zCompositions_SQ	1.000	0.000	0.000	0.000
zCompositions_CZM	1.000	0.000	0.000	0.000

Table S5. Dataset 2 - Sensitivity and specificity on species presence/absence aggregated by imputation method. Sensitivity and specificity values in classifying species presence/absence are aggregated according to the imputation method used in the pipelines and are reported as mean and standard deviation (SD) values calculated among normalization methods.

	Sensitivity		Specificity	
**Imputation pipeline**	**mean**	**SD**	**mean**	**SD**
None (Normalization only)	0.000	0.000	1.000	0.000
DrImpute	0.997	0.000	0.747	0.000
scImpute	0.983	0.001	0.983	0.000
LLSimpute	0.728	0.024	0.355	0.027
zCompositions_SQ	1.000	0.000	0.000	0.000
zCompositions_CZM	1.000	0.000	0.000	0.000

Table S6. Dataset 3 - Sensitivity and specificity on species presence/absence aggregated by imputation method. Sensitivity and specificity values in classifying species presence/absence are aggregated according to the imputation method used in the pipelines and are reported as mean and standard deviation (SD) values calculated among normalization methods.

	Sensitivity		Specificity	
**Imputation pipeline**	**mean**	**SD**	**mean**	**SD**
None (Normalization only)	0.000	0.000	1.000	0.000
DrImpute	0.968	0.001	0.876	0.016
scImpute	0.780	0.002	0.946	0.000
LLSimpute	0.734	0.018	0.244	0.018
zCompositions_SQ	1.000	0.000	0.000	0.000
zCompositions_CZM	1.000	0.000	0.000	0.000

Table S7. Specificity and sensitivity on species presence/absence. For each dataset and each pipeline, the values of sensitivity and specificity in classifying species presence/absence are reported.

	Dataset 1		Dataset 2		Dataset 3	
**Pipeline**	**sensitivity**	**specificity**	**sensitivity**	**specificity**	**sensitivity**	**specificity**
TSS	0.000	1.000	0.000	1.000	0.000	1.000
CSS	0.000	1.000	0.000	1.000	0.000	1.000
edgeR	0.000	1.000	0.000	1.000	0.000	1.000
DESeq2	0.000	1.000	0.000	1.000	0.000	1.000
GMPR	0.000	1.000	0.000	1.000	0.000	1.000
None_DrImpute	0.971	0.991	0.997	0.747	0.970	0.865
None_scImpute	0.316	0.999	0.982	0.983	0.780	0.946
None_LLSimpute	0.758	0.361	0.708	0.356	0.708	0.250
None_zCompositions_SQ	1.000	0.000	1.000	0.000	1.000	0.000
None_zCompositions_CZM	1.000	0.000	1.000	0.000	1.000	0.000
TSS_DrImpute	0.986	0.992	0.997	0.747	0.967	0.896
TSS_scImpute	0.316	0.999	0.983	0.983	0.781	0.946
TSS_LLSimpute	0.740	0.389	0.759	0.348	0.727	0.271
TSS_zCompositions_SQ	1.000	0.000	1.000	0.000	1.000	0.000
TSS_zCompositions_CZM	1.000	0.000	1.000	0.000	1.000	0.000
CSS_DrImpute	0.986	0.992	0.997	0.747	0.967	0.868
CSS_scImpute	0.316	0.999	0.983	0.983	0.781	0.946
CSS_LLSimpute	0.837	0.292	0.716	0.368	0.729	0.240
CSS_zCompositions_SQ	1.000	0.000	1.000	0.000	1.000	0.000
CSS_zCompositions_CZM	1.000	0.000	1.000	0.000	1.000	0.000
edgeR_DrImpute	0.986	0.992	0.997	0.747	0.967	0.896
edgeR_scImpute	0.316	0.999	0.983	0.983	0.782	0.946
edgeR_LLSimpute	0.839	0.300	0.711	0.384	0.762	0.215
edgeR_zCompositions_SQ	1.000	0.000	1.000	0.000	1.000	0.000
edgeR_zCompositions_CZM	1.000	0.000	1.000	0.000	1.000	0.000
DESeq2_DrImpute	0.986	0.992	0.997	0.747	0.968	0.865
DESeq2_scImpute	0.316	0.999	0.983	0.983	0.778	0.946
DESeq2_LLSimpute	0.839	0.287	0.716	0.371	0.732	0.249
DESeq2_zCompositions_SQ	1.000	0.000	1.000	0.000	1.000	0.000
DESeq2_zCompositions_CZM	1.000	0.000	1.000	0.000	1.000	0.000
GMPR_DrImpute	0.986	0.992	0.997	0.747	0.968	0.865
GMPR_scImpute	0.316	0.999	0.983	0.983	0.779	0.946
GMPR_LLSimpute	0.840	0.286	0.758	0.306	0.743	0.240
GMPR_zCompositions_SQ	1.000	0.000	1.000	0.000	1.000	0.000
GMPR_zCompositions_CZM	1.000	0.000	1.000	0.000	1.000	0.000

Relative abundance profile

Figure S1. Simulated Dataset 1 – SMAPE on relative abundance profile. For each pipeline and sample, SMAPE was computed between the relative abundance in ground truth data and the relative abundance in pre-processed data. For each pipeline, the boxplots show the distribution of SMAPE values. Distributions of SMAPE values that resulted statistically lower (one-sided Mann-Whitney paired U-test, Benjamini-Hochberg correction, significant threshold 0.05) than SMAPE values on raw data are indicated with the symbol "*", followed by the interpretation of Cohen's d effect size (N: negligible, VS: very small, S: small, M: medium, L: large, VL: very large, H: huge). The vertical dashed line indicates the median SMAPE value of raw data.

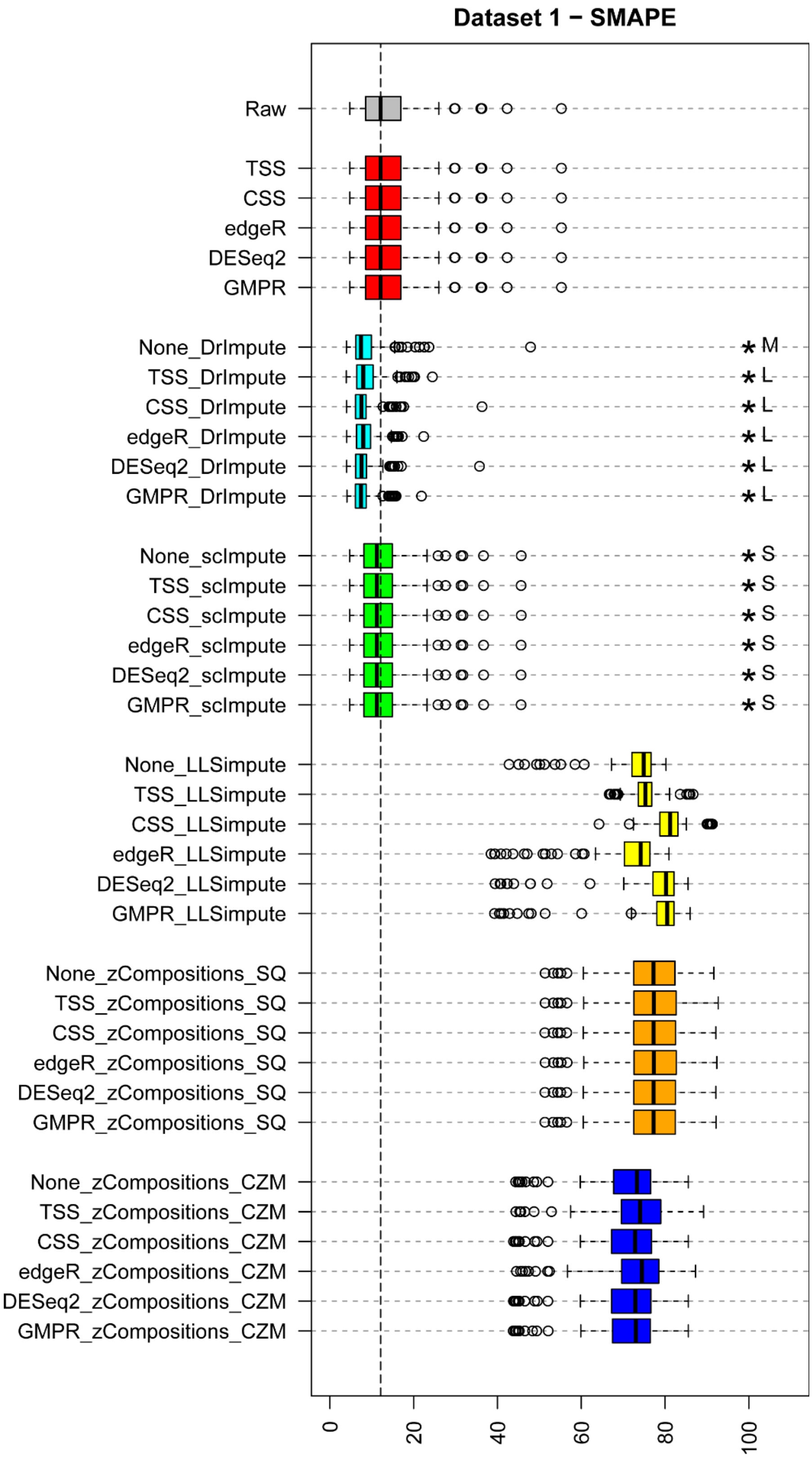

Figure S2. Simulated Dataset 2 – SMAPE on relative abundance profile. For each pipeline and sample, SMAPE was computed between the relative abundance in ground truth data and the relative abundance in pre-processed data. For each pipeline, the boxplots show the distribution of SMAPE values. Distributions of SMAPE values that resulted statistically lower (one-sided Mann-Whitney paired U-test, Benjamini-Hochberg correction, significant threshold 0.05) than SMAPE values on raw data are indicated with the symbol "*", followed by the interpretation of Cohen's d effect size (N: negligible, VS: very small, S: small, M: medium, L: large, VL: very large, H: huge). The vertical dashed line indicates the median SMAPE value of raw data.

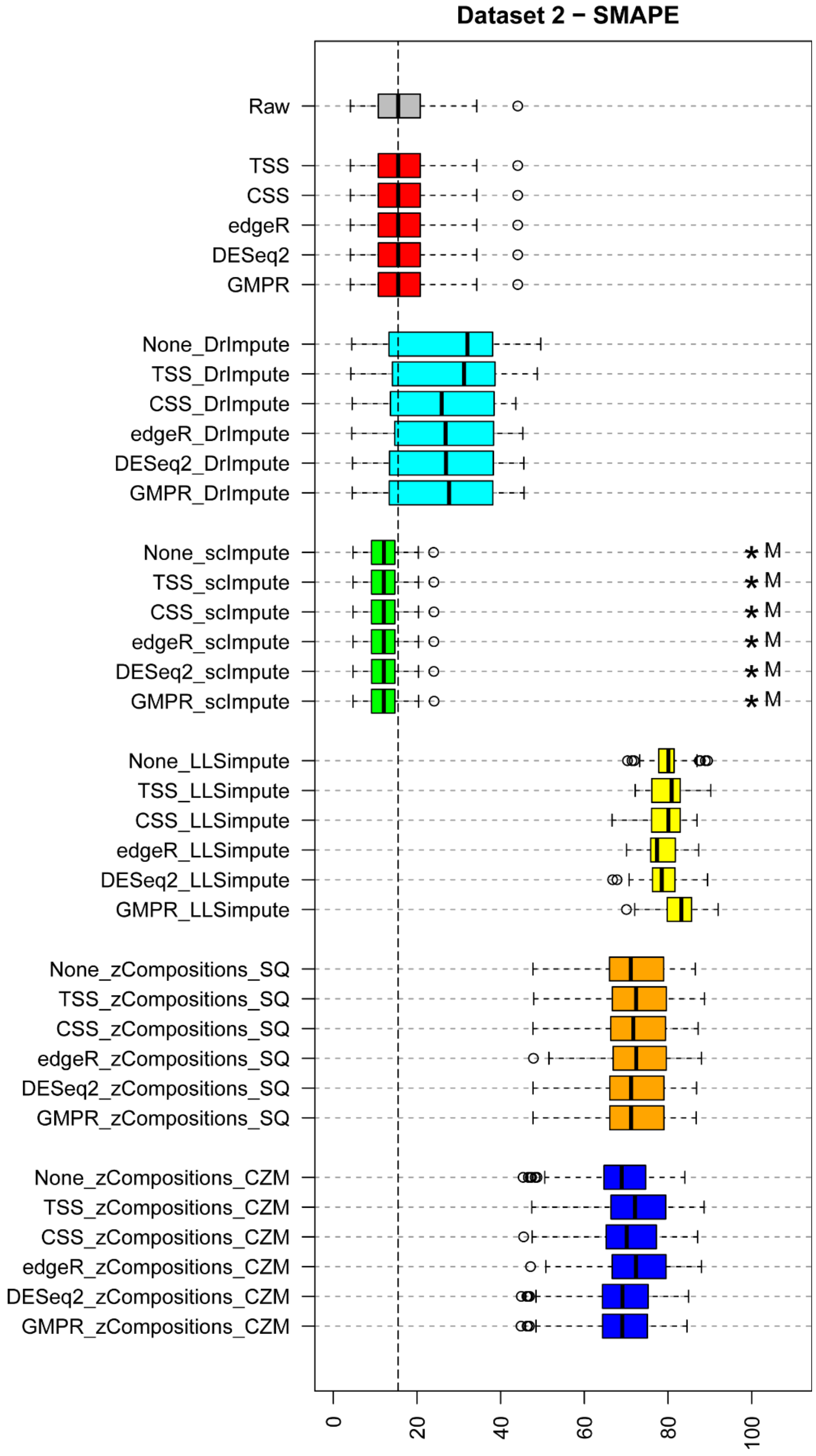

Figure S3. Simulated Dataset 3 – SMAPE on relative abundance profile. For each pipeline and sample, SMAPE was computed between the relative abundance in ground truth data and the relative abundance in pre-processed data. For each pipeline, the boxplots show the distribution of SMAPE values. Distributions of SMAPE values that resulted statistically lower (one-sided Mann-Whitney paired U-test, Benjamini-Hochberg correction, significant threshold 0.05) than SMAPE values on raw data are indicated with the symbol "*", followed by the interpretation of Cohen's d effect size (N: negligible, VS: very small, S: small, M: medium, L: large, VL: very large, H: huge). The vertical dashed line indicates the median SMAPE value of raw data.

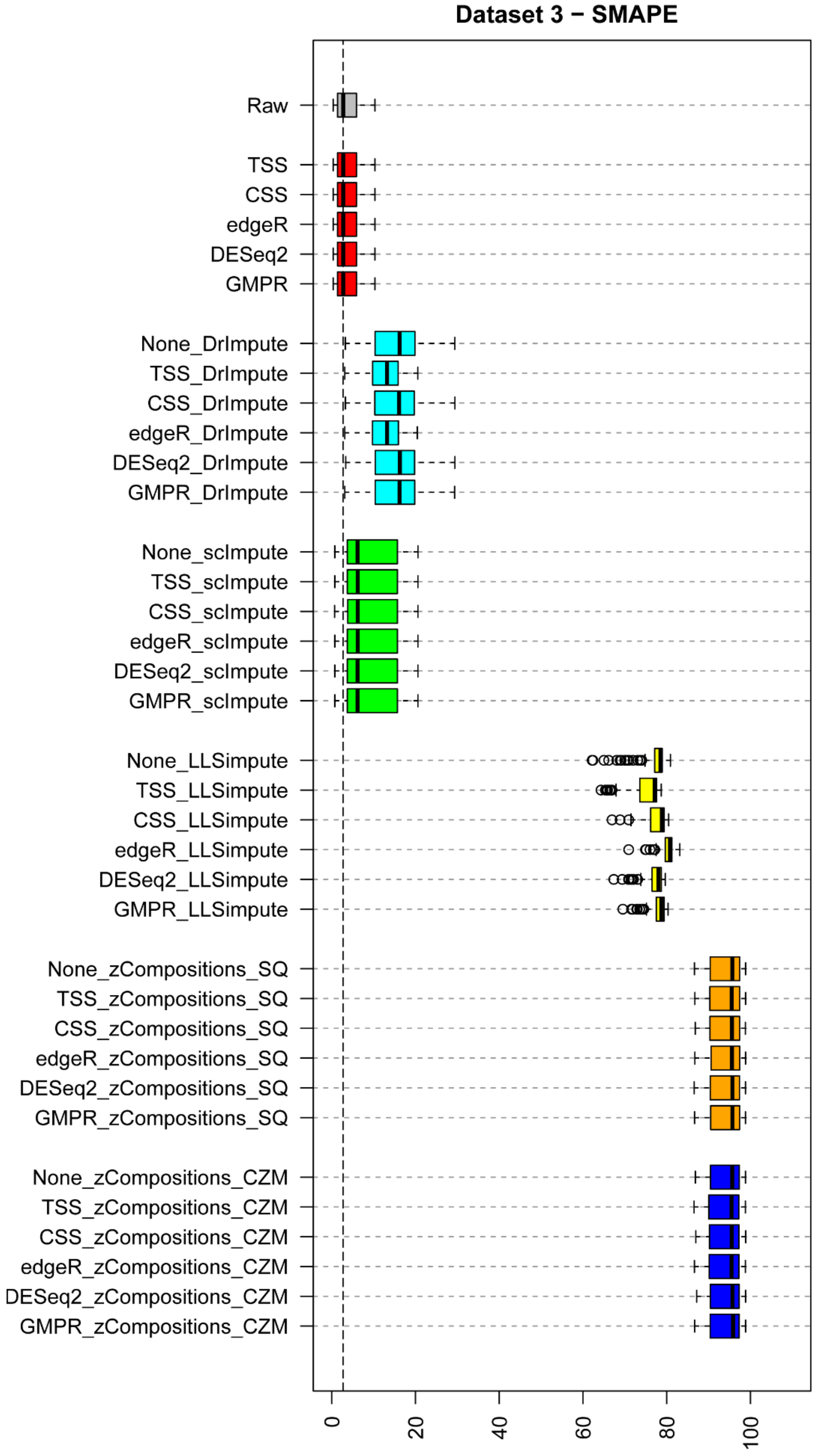

Table S8. Results of statistical analysis on SMAPE values on relative abundance profile. For each dataset, pipeline and sample, a SMAPE value was computed between relative abundance in ground truth data and relative abundance in pre-processed data. For each of the three test datasets and for each pipeline, the corrected p-values (one-sided Mann-Whitney paired U-test on raw data SMAPE vs pre-processed data SMAPE, Benjamini-Hochberg correction, significant threshold 0.05) and the effect sizes are reported. p-values associated to statistically significant comparisons are indicated with the symbol "*" close to the p-value value. The interpretation of Cohen's d effect size is reported in brackets close to the related value: (N) negligible, (VS) very small, (S) small, (M) medium, (L) large, (VL) very large, (H) huge.

	Dataset 1		Dataset 2		Dataset 3	
Pipeline	Corrected p-value	Effect size	Corrected p-value	Effect size	Corrected p-value	Effect size
TSS	1.000	0 (N)	1.000	0 (N)	1.000	0 (N)
CSS	1.000	0 (N)	1.000	0 (N)	1.000	0 (N)
edgeR	1.000	0 (N)	1.000	0 (N)	1.000	0 (N)
DESeq2	1.000	0 (N)	1.000	0 (N)	1.000	0 (N)
GMPR	1.000	0 (N)	1.000	0 (N)	1.000	0 (N)
None_DrImpute	3.81E-22*	0.764 (M)	1.000	-0.818 (L)	1.000	-2.476 (H)
TSS_DrImpute	1.23E-22*	0.853 (L)	1.000	-0.877 (L)	1.000	-2.529 (H)
CSS_DrImpute	7.60E-23*	0.968 (L)	1.000	-0.766 (M)	1.000	-2.375 (H)
edgeR_DrImpute	1.23E-22*	0.947 (L)	1.000	-0.846 (L)	1.000	-2.534 (H)
DESeq2_DrImpute	7.60E-23*	0.981 (L)	1.000	-0.761 (M)	1.000	-2.488 (H)
GMPR_DrImpute	7.60E-23*	1.050 (L)	1.000	-0.762 (M)	1.000	-2.484 (H)
None_scImpute	2.52E-23*	0.212 (S)	1.46E-12*	0.690 (M)	1.000	-1.100 (L)
TSS_scImpute	2.52E-23*	0.212 (S)	1.46E-12*	0.690 (M)	1.000	-1.102 (L)
CSS_scImpute	2.52E-23*	0.212 (S)	1.46E-12*	0.690 (M)	1.000	-1.101 (L)
edgeR_scImpute	2.52E-23*	0.212 (S)	1.46E-12*	0.690 (M)	1.000	-1.103 (L)
DESeq2_scImpute	2.52E-23*	0.212 (S)	1.46E-12*	0.690 (M)	1.000	-1.098 (L)
GMPR_scImpute	2.52E-23*	0.212 (S)	1.46E-12*	0.690 (M)	1.000	-1.098 (L)
None_LLSimpute	1.000	-8.203 (H)	1.000	-10.026 (H)	1.000	-23.310 (H)
TSS_LLSimpute	1.000	-10.286 (H)	1.000	-9.665 (H)	1.000	-22.522 (H)
CSS_LLSimpute	1.000	-10.883 (H)	1.000	-9.228 (H)	1.000	-28.022 (H)
edgeR_LLSimpute	1.000	-6.829 (H)	1.000	-9.655 (H)	1.000	-33.595 (H)
DESeq2_LLSimpute	1.000	-7.353 (H)	1.000	-9.289 (H)	1.000	-29.507 (H)
GMPR_LLSimpute	1.000	-7.400 (H)	1.000	-10.015 (H)	1.000	-30.353 (H)
None_zCompositions_SQ	1.000	-8.211 (H)	1.000	-6.280 (H)	1.000	-27.245 (H)
TSS_zCompositions_SQ	1.000	-8.153 (H)	1.000	-6.257 (H)	1.000	-27.338 (H)
CSS_zCompositions_SQ	1.000	-8.173 (H)	1.000	-6.243 (H)	1.000	-27.391 (H)
edgeR_zCompositions_SQ	1.000	-8.162 (H)	1.000	-6.280 (H)	1.000	-27.424 (H)
DESeq2_zCompositions_SQ	1.000	-8.173 (H)	1.000	-6.236 (H)	1.000	-27.279 (H)
GMPR_zCompositions_SQ	1.000	-8.166 (H)	1.000	-6.238 (H)	1.000	-27.285 (H)
None_zCompositions_CZM	1.000	-7.217 (H)	1.000	-5.833 (H)	1.000	-27.161 (H)
TSS_zCompositions_CZM	1.000	-7.325 (H)	1.000	-6.176 (H)	1.000	-26.572 (H)
CSS_zCompositions_CZM	1.000	-7.114 (H)	1.000	-5.933 (H)	1.000	-27.027 (H)
edgeR_zCompositions_CZM	1.000	-7.400 (H)	1.000	-6.225 (H)	1.000	-26.690 (H)
DESeq2_zCompositions_CZM	1.000	-7.112 (H)	1.000	-5.794 (H)	1.000	-27.473 (H)
GMPR_zCompositions_CZM	1.000	-7.092 (H)	1.000	-5.799 (H)	1.000	-26.824 (H)

Figure S4. Simulated Dataset 1 – Aitchison's distance on relative abundance profile. For each pipeline and sample, Aitchison's distance was computed between the relative abundance in ground truth data and the relative abundance in pre-processed data. For each pipeline, the boxplots show the distribution of Aitchison's distance values. Distributions of Aitchison's distance values that result statistically lower (one-sided Mann-Whitney paired U-test, Benjamini-Hochberg correction, significant threshold 0.05) than Aitchison's distance values on raw data are indicated with the symbol "*", followed by the interpretation of Cohen's d effect size (N: negligible, VS: very small, S: small, M: medium, L: large, VL: very large, H: huge). The vertical dashed line indicates the median Aitchison's distance of raw data.

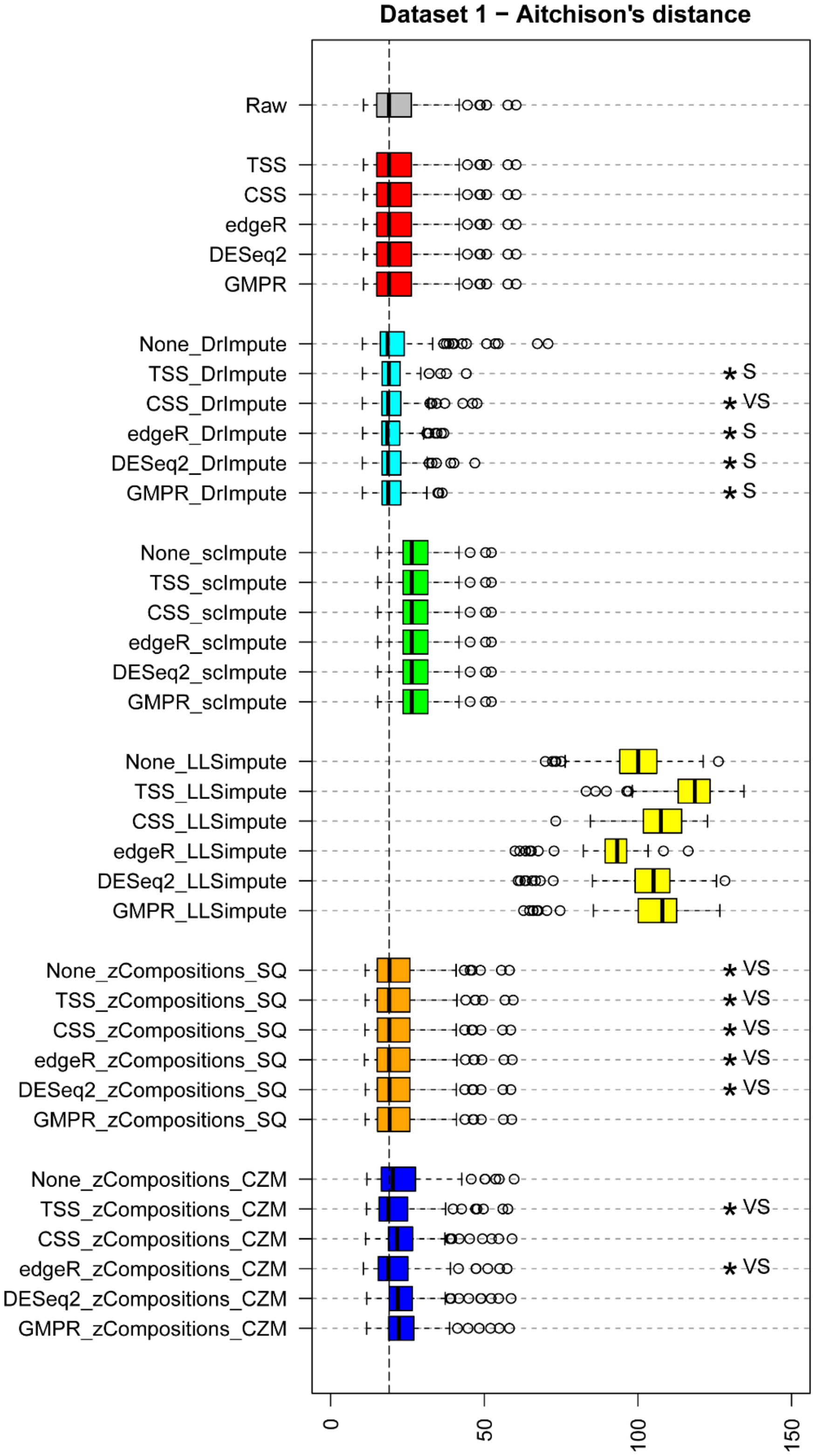

Figure S5. Simulated Dataset 2 – Aitchison's distance on relative abundance profile. For each pipeline and sample, Aitchison's distance was computed between the relative abundance in ground truth data and the relative abundance in pre-processed data. For each pipeline, the boxplots show the distribution of Aitchison's distance values. Distributions of Aitchison's distance values that result statistically lower (one-sided Mann-Whitney paired U-test, Benjamini-Hochberg correction, significant threshold 0.05) than Aitchison's distance values on raw data are indicated with the symbol "*", followed by the interpretation of Cohen's d effect size (N: negligible, VS: very small, S: small, M: medium, L: large, VL: very large, H: huge). The vertical dashed line indicates the median Aitchison's distance of raw data.

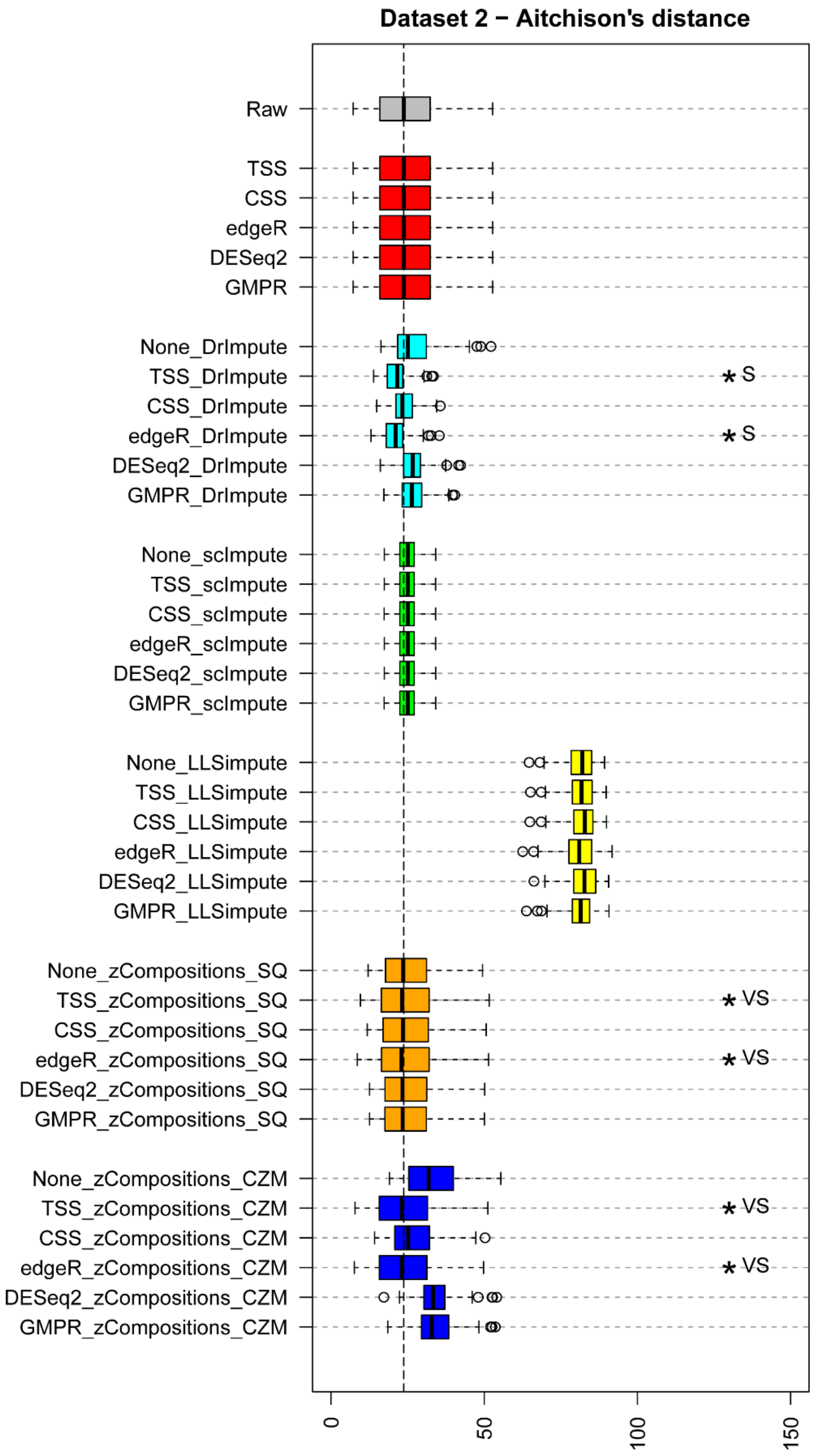

Figure S6. Simulated Dataset 3 – Aitchison's distance on relative abundance profile. For each pipeline and sample, Aitchison's distance was computed between the relative abundance in ground truth data and the relative abundance in pre-processed data. For each pipeline, the boxplots show the distribution of Aitchison's distance values. Distributions of Aitchison's distance values that result statistically lower (one-sided Mann-Whitney paired U-test, Benjamini-Hochberg correction, significant threshold 0.05) than Aitchison's distance values on raw data are indicated with the symbol "*", followed by the interpretation of Cohen's d effect size (N: negligible, VS: very small, S: small, M: medium, L: large, VL: very large, H: huge). The vertical dashed line indicates the median Aitchison's distance of raw data.

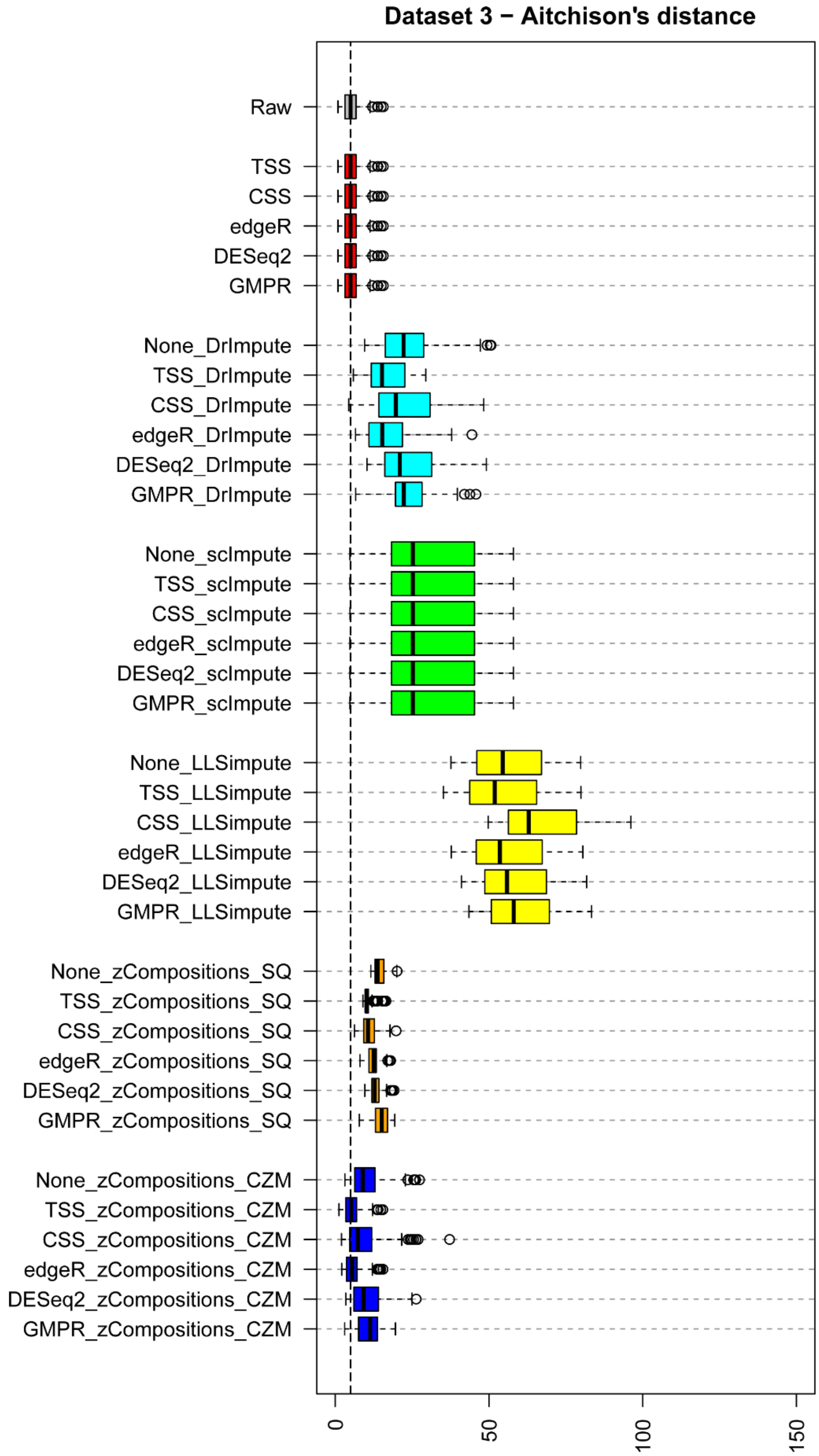

Table S9. Results of statistical analysis on Aitchison's distance on relative abundance profile. For each dataset, pipeline and sample, Aitchison distance was computed between relative abundance in ground truth data and relative abundance in pre-processed data. For each of the three test datasets and for each pipeline, the corrected p-values (one-sided Mann-Whitney paired U-test on raw data Aitchison distance vs pre-processed data Aitchison distance, Benjamini-Hochberg correction, significant threshold 0.05) and the effect sizes are reported. p-values associated to statistically significant comparisons are indicated with the symbol "*" close to the p-value value. The interpretation of Cohen's d effect size is reported in brackets close to the related value: (N) negligible, (VS) very small, (S) small, (M) medium, (L) large, (VL) very large, (H) huge.

	Dataset 1		Dataset 2		Dataset 3	
Pipeline	Corrected p-value	Effect size	Corrected p-value	Effect size	Corrected p-value	Effect size
TSS	1.000	0.000 (N)	1.000	0.000 (N)	1.000	0.000 (N)
CSS	1.000	0.000 (N)	1.000	0.000 (N)	1.000	0.000 (N)
edgeR	1.000	0.000 (N)	1.000	0.000 (N)	1.000	0.000 (N)
DESeq2	1.000	0.000 (N)	1.000	0.000 (N)	1.000	0.000 (N)
GMPR	1.000	0.000 (N)	1.000	0.000 (N)	1.000	0.000 (N)
None_DrImpute	0.802	-0.001 (N)	1.000	-0.274 (S)	1.000	-2.544 (H)
TSS_DrImpute	0.009*	0.230 (S)	0.011*	0.379 (S)	1.000	-2.057 (H)
CSS_DrImpute	0.008*	0.189 (VS)	1.000	0.107 (VS)	1.000	-2.267 (H)
edgeR_DrImpute	0.003*	0.264 (S)	0.003*	0.413 (S)	1.000	-1.910 (VL)
DESeq2_DrImpute	0.008*	0.206 (S)	1.000	-0.214 (S)	1.000	-2.617 (H)
GMPR_DrImpute	0.008*	0.244 (S)	1.000	-0.225 (S)	1.000	-3.263 (H)
None_scImpute	1.000	-0.748 (M)	1.000	-0.038 (VS)	1.000	-2.222 (H)
TSS_scImpute	1.000	-0.748 (M)	1.000	-0.038 (VS)	1.000	-2.222 (H)
CSS_scImpute	1.000	-0.748 (M)	1.000	-0.038 (VS)	1.000	-2.222 (H)
edgeR_scImpute	1.000	-0.748 (M)	1.000	-0.038 (VS)	1.000	-2.222 (H)
DESeq2_scImpute	1.000	-0.748 (M)	1.000	-0.038 (VS)	1.000	-2.222 (H)
GMPR_scImpute	1.000	-0.748 (M)	1.000	-0.038 (VS)	1.000	-2.222 (H)
None_LLSimpute	1.000	-7.528 (H)	1.000	-6.433 (H)	1.000	-6.021 (H)
TSS_LLSimpute	1.000	-10.077 (H)	1.000	-6.486 (H)	1.000	-5.553 (H)
CSS_LLSimpute	1.000	-9.177 (H)	1.000	-6.557 (H)	1.000	-7.050 (H)
edgeR_LLSimpute	1.000	-7.618 (H)	1.000	-6.199 (H)	1.000	-5.943 (H)
DESeq2_LLSimpute	1.000	-7.137 (H)	1.000	-6.533 (H)	1.000	-6.407 (H)
GMPR_LLSimpute	1.000	-7.319 (H)	1.000	-6.414 (H)	1.000	-6.948 (H)
None_zCompositions_SQ	0.031*	0.019 (VS)	1.000	-0.005 (N)	1.000	-3.398 (H)
TSS_zCompositions_SQ	0.008*	0.013 (VS)	0.002*	0.033 (VS)	1.000	-2.123 (H)
CSS_zCompositions_SQ	0.008*	0.020 (VS)	0.395	0.022 (VS)	1.000	-1.941 (VL)
edgeR_zCompositions_SQ	0.002*	0.018 (VS)	0.001*	0.041 (VS)	1.000	-2.565 (H)
DESeq2_zCompositions_SQ	0.026*	0.017 (VS)	1.000	-0.008 (N)	1.000	-2.949 (H)
GMPR_zCompositions_SQ	0.069	0.014 (VS)	1.000	-0.004 (N)	1.000	-3.403 (H)
None_zCompositions_CZM	1.000	-0.151 (VS)	1.000	-0.807 (L)	1.000	-1.093 (L)
TSS_zCompositions_CZM	0.016*	0.030 (VS)	0.000*	0.045 (VS)	1.000	-0.072 (VS)
CSS_zCompositions_CZM	1.000	-0.210 (S)	1.000	-0.207 (S)	1.000	-0.778 (M)
edgeR_zCompositions_CZM	0.001*	0.049 (VS)	0.000*	0.057 (VS)	1.000	-0.165 (VS)
DESeq2_zCompositions_CZM	1.000	-0.226 (S)	1.000	-1.017 (L)	1.000	-1.167 (L)
GMPR_zCompositions_CZM	1.000	-0.250 (S)	1.000	-0.981 (L)	1.000	-1.563 (VL)

Table S10. Simulated Dataset 1 - Pipelines median SMAPE and Aitchison's distance to the ground truth and their ranking. Pipelines are sorted by increasing rank on SMAPE.

Pipeline	SMAPE	Rank (SMAPE)	Aitchison dist	Rank (Aitchison dist)
GMPR_DrImpute	7.416	1	18.740	5
None_DrImpute	7.421	2	18.581	2
CSS_DrImpute	7.538	3	18.729	4
DESeq2_DrImpute	7.554	4	18.664	3
edgeR_DrImpute	7.976	5	18.437	1
TSS_DrImpute	7.981	6	18.944	8
DESeq2_scImpute	11.191	7	26.410	30
CSS_scImpute	11.192	8	26.407	25
TSS_scImpute	11.192	9	26.408	27
edgeR_scImpute	11.193	10	26.409	29
GMPR_scImpute	11.193	11	26.409	28
None_scImpute	11.194	12	26.407	26
CSS	12.109	13	19.039	9
DESeq2	12.109	13	19.039	9
edgeR	12.109	13	19.039	9
GMPR	12.109	13	19.039	9
Raw	12.109	13	19.039	9
TSS	12.109	13	19.039	9
CSS_zCompositions_CZM	72.843	19	21.733	22
DESeq2_zCompositions_CZM	72.904	20	21.819	23
GMPR_zCompositions_CZM	73.031	21	22.253	24
None_zCompositions_CZM	73.270	22	20.250	21
TSS_zCompositions_CZM	74.034	23	18.816	7
edgeR_LLSimpute	74.167	24	93.237	31
edgeR_zCompositions_CZM	74.478	25	18.815	6
None_LLSimpute	74.894	26	100.103	32
TSS_LLSimpute	75.301	27	118.484	36
None_zCompositions_SQ	77.230	28	19.171	19
GMPR_zCompositions_SQ	77.257	29	19.186	20
DESeq2_zCompositions_SQ	77.260	30	19.165	18
CSS_zCompositions_SQ	77.263	31	19.137	17
TSS_zCompositions_SQ	77.335	32	19.107	16
edgeR_zCompositions_SQ	77.337	33	19.081	15
DESeq2_LLSimpute	80.231	34	105.101	33
GMPR_LLSimpute	80.508	35	107.936	35
CSS_LLSimpute	81.203	36	107.501	34

Table S11. Simulated Dataset 2 - Pipelines median SMAPE and Aitchison's distance to the ground truth and their ranking. Pipelines are sorted by increasing rank on SMAPE.

Pipeline	SMAPE	Rank (SMAPE)	Aitchison dist	Rank (Aitchison dist)
DESeq2_scImpute	12.050	1	25.112	19
GMPR_scImpute	12.055	2	25.111	18
CSS_scImpute	12.058	3	25.112	21
edgeR_scImpute	12.061	4	25.113	23
TSS_scImpute	12.061	5	25.113	22
None_scImpute	12.071	6	25.112	20
CSS	15.493	7	23.754	12
DESeq2	15.493	7	23.754	12
edgeR	15.493	7	23.754	12
GMPR	15.493	7	23.754	12
Raw	15.493	7	23.754	12
TSS	15.493	7	23.754	12
CSS_DrImpute	25.896	13	23.332	7
edgeR_DrImpute	26.834	14	21.106	1
DESeq2_DrImpute	26.923	15	26.652	27
GMPR_DrImpute	27.666	16	26.408	26
TSS_DrImpute	31.247	17	21.650	2
None_DrImpute	32.057	18	25.115	24
None_zCompositions_CZM	68.935	19	31.894	28
GMPR_zCompositions_CZM	69.064	20	32.851	29
DESeq2_zCompositions_CZM	69.092	21	33.317	30
CSS_zCompositions_CZM	70.130	22	25.202	25
None_zCompositions_SQ	71.112	23	23.583	11
DESeq2_zCompositions_SQ	71.173	24	23.362	8
GMPR_zCompositions_SQ	71.179	25	23.388	9
CSS_zCompositions_SQ	71.686	26	23.509	10
TSS_zCompositions_CZM	72.091	27	23.103	4
edgeR_zCompositions_CZM	72.332	28	23.133	5
TSS_zCompositions_SQ	72.340	29	23.232	6
edgeR_zCompositions_SQ	72.391	30	23.051	3
edgeR_LLSimpute	77.375	31	81.045	31
DESeq2_LLSimpute	78.496	32	82.723	35
CSS_LLSimpute	80.079	33	82.841	36
None_LLSimpute	80.079	33	82.002	34
TSS_LLSimpute	80.871	35	81.793	33
GMPR_LLSimpute	83.179	36	81.469	32

Table S12. Simulated Dataset 3 - Pipelines median SMAPE and Aitchison's distance to the ground truth and their ranking. Pipelines are sorted by increasing rank on SMAPE.

Pipeline	SMAPE	Rank (SMAPE)	Aitchison dist	Rank (Aitchison dist)
CSS	2.717	1	4.972	1
DESeq2	2.717	1	4.972	1
edgeR	2.717	1	4.972	1
GMPR	2.717	1	4.972	1
Raw	2.717	1	4.972	1
TSS	2.717	1	4.972	1
GMPR_scImpute	6.155	7	25.241	30
None_scImpute	6.157	8	25.238	25
DESeq2_scImpute	6.158	9	25.240	28
edgeR_scImpute	6.216	10	25.240	26
TSS_scImpute	6.218	11	25.240	27
CSS_scImpute	6.221	12	25.240	29
TSS_DrImpute	13.165	13	15.219	19
edgeR_DrImpute	13.197	14	15.275	20
CSS_DrImpute	16.065	15	19.696	21
GMPR_DrImpute	16.198	16	22.287	24
None_DrImpute	16.216	17	22.201	23
DESeq2_DrImpute	16.280	18	21.002	22
TSS_LLSimpute	77.105	19	51.852	31
DESeq2_LLSimpute	77.982	20	55.867	34
None_LLSimpute	78.509	21	54.433	33
GMPR_LLSimpute	78.666	22	58.037	35
CSS_LLSimpute	78.839	23	62.896	36
edgeR_LLSimpute	80.770	24	53.555	32
TSS_zCompositions_CZM	95.467	25	5.185	7
edgeR_zCompositions_CZM	95.487	26	5.475	8
TSS_zCompositions_SQ	95.532	27	10.121	12
CSS_zCompositions_CZM	95.539	28	7.329	9
CSS_zCompositions_SQ	95.568	29	10.636	13
edgeR_zCompositions_SQ	95.584	30	12.489	15
None_zCompositions_CZM	95.621	31	8.941	10
None_zCompositions_SQ	95.649	32	13.788	17
DESeq2_zCompositions_SQ	95.675	33	12.857	16
GMPR_zCompositions_SQ	95.676	34	15.097	18
DESeq2_zCompositions_CZM	95.697	35	9.220	11
GMPR_zCompositions_CZM	95.816	36	11.366	14

Impact on bacterial diversity

Figure S7. Simulated dataset 1 - Relative error on Richness alpha diversity indices. (The lower the better). Relative errors are computed between alpha indices value from ground truth data and the corresponding alpha indices values obtained from pre-processed data. Distributions of relative error values that result statistically lower (one-sided paired Mann-Whitney paired U-test, Benjamini-Hochberg correction, significant threshold 0.05) than relative errors calculated on raw or normalized-only data are indicated with the symbol "*", followed by the interpretation of Cohen's d effect size (N: negligible, VS: very small, S: small, M: medium, L: large, VL: very large, H: huge). The vertical dashed line indicates the median relative error on raw and normalized-only data.

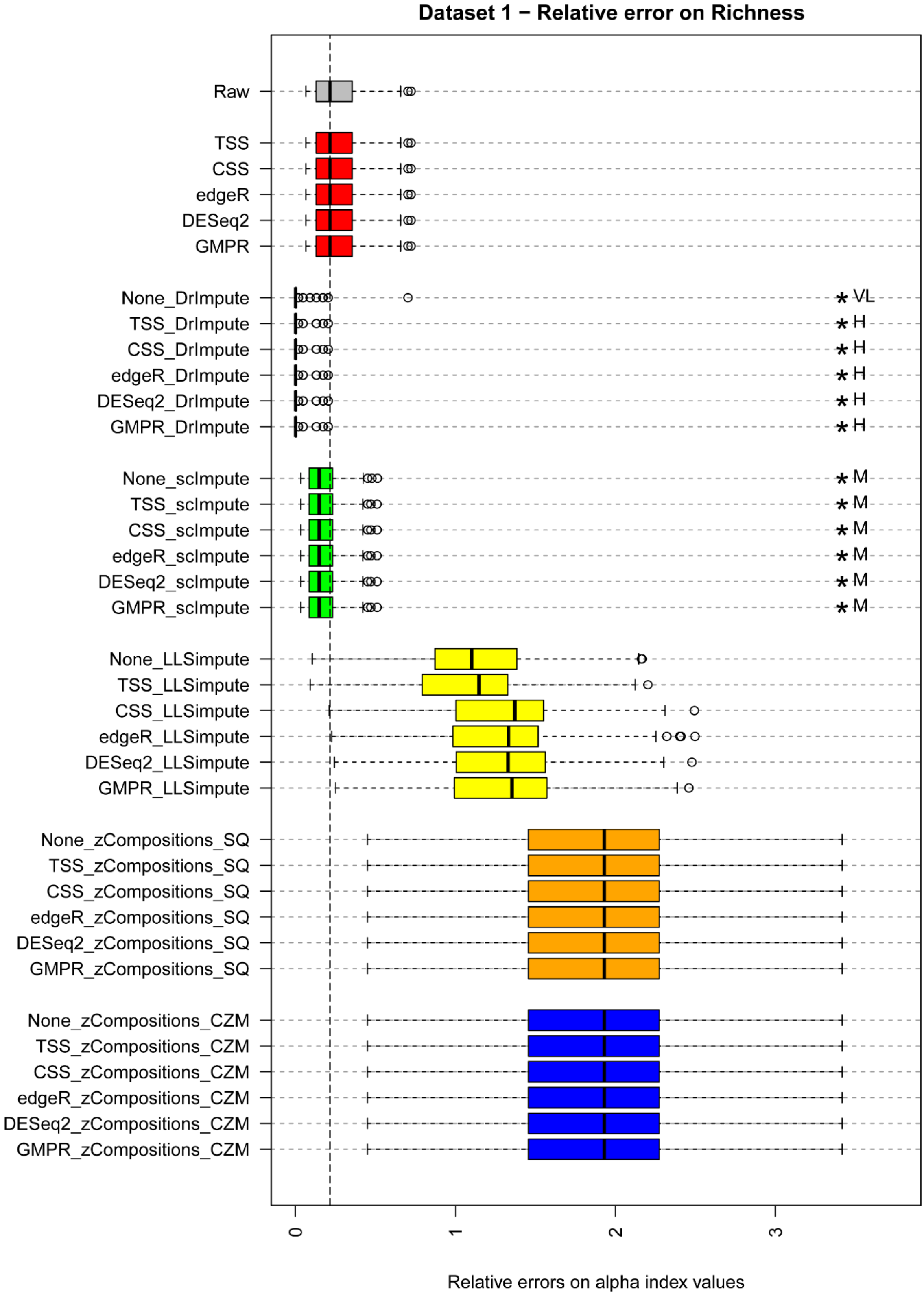

Figure S8. Simulated dataset 2 - Relative error on Richness alpha diversity indices. (The lower the better). Relative errors are computed between alpha indices value from ground truth data and the corresponding alpha indices values obtained from pre-processed data. Distributions of relative error values that result statistically lower (one-sided paired Mann-Whitney paired U-test, Benjamini-Hochberg correction, significant threshold 0.05) than relative errors calculated on raw or normalized-only data are indicated with the symbol "*", followed by the interpretation of Cohen's d effect size (N: negligible, VS: very small, S: small, M: medium, L: large, VL: very large, H: huge). The vertical dashed line indicates the median relative error on raw and normalized-only data.

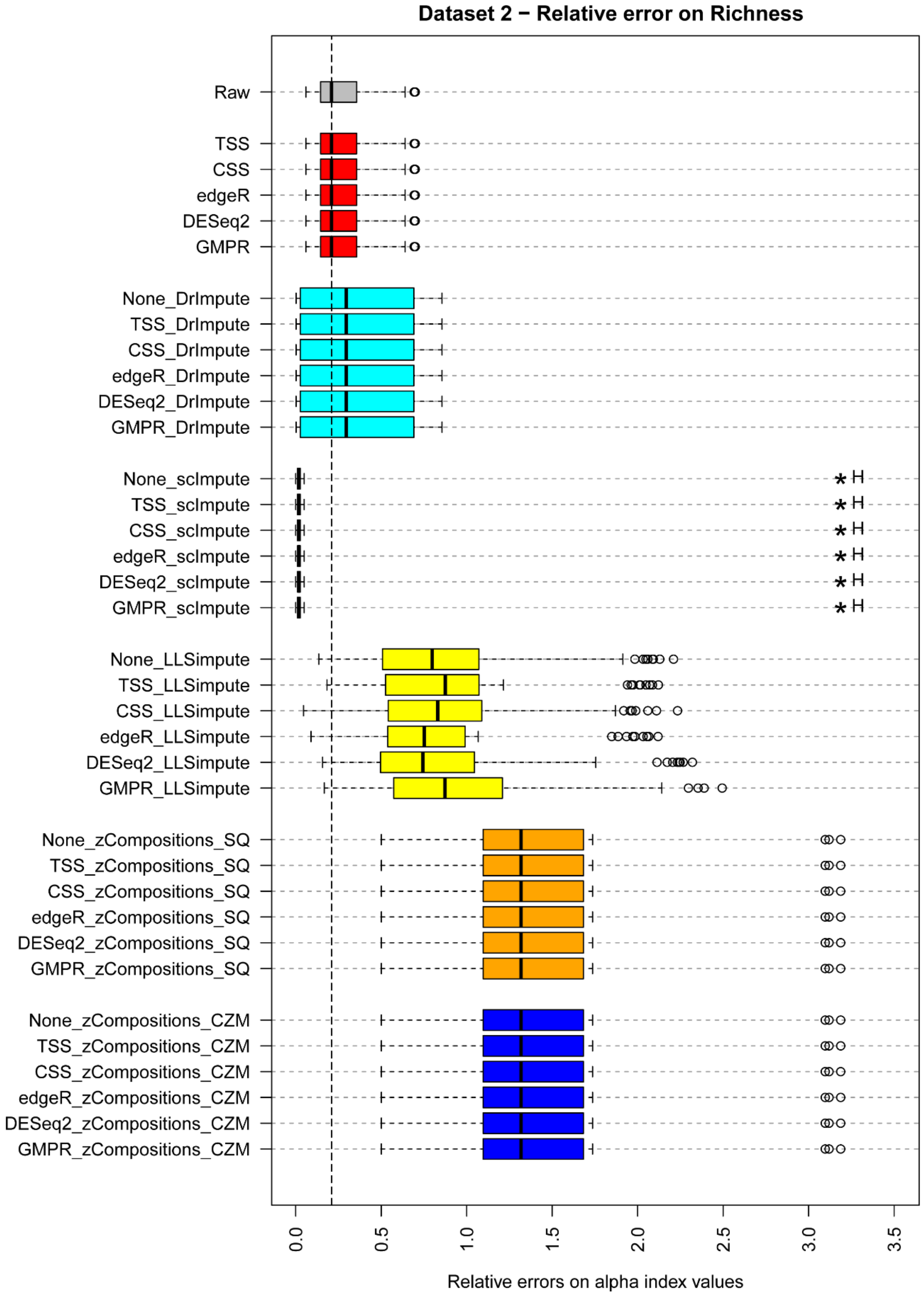

Figure S9. Simulated dataset 3 - Relative error on Richness alpha diversity indices. (The lower the better). Relative errors are computed between alpha indices value from ground truth data and the corresponding alpha indices values obtained from pre-processed data. Distributions of relative error values that result statistically lower (one-sided paired Mann-Whitney paired U-test, Benjamini-Hochberg correction, significant threshold 0.05) than relative errors calculated on raw or normalized-only data are indicated with the symbol "*", followed by the interpretation of Cohen's d effect size (N: negligible, VS: very small, S: small, M: medium, L: large, VL: very large, H: huge). The vertical dashed line indicates the median relative error on raw and normalized-only data.

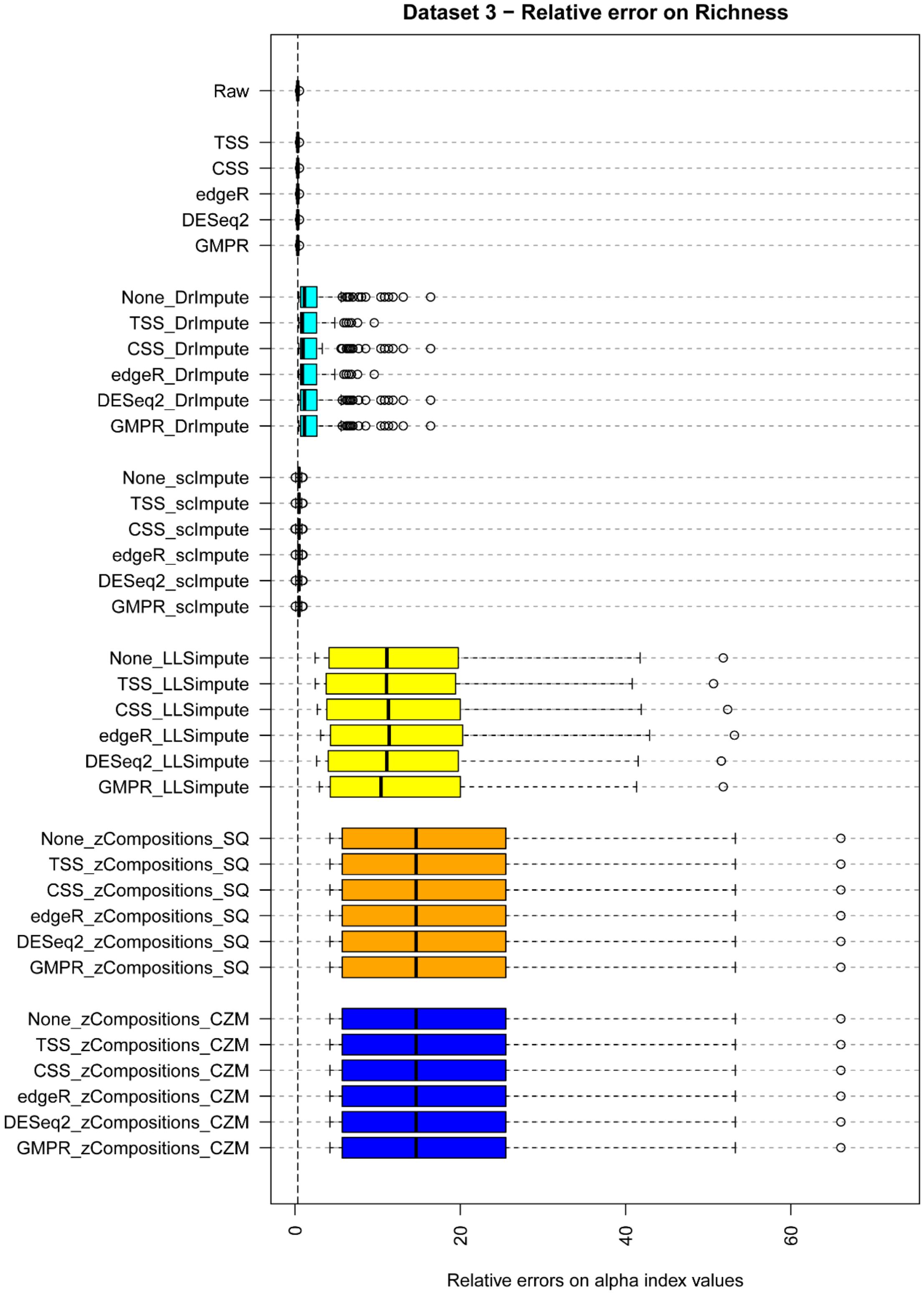

Table S13. Results of statistical analysis on relative error between ground truth and pre-processed data Richness indices. The analysis aims at identifying pipeline achieving relative error values lower than the ones obtained using raw data (or normalized-only pipelines). For each of the three test datasets and for each pipeline, the corrected p-values (one-sided paird Mann-Whitney paired U-test, Benjamini-Hochberg correction, significant threshold 0.05) and the effect sizes are reported. p-values associated to statistically significant comparisons are indicated with the symbol "*" close to the p-value value. The interpretation of Cohen's d effect size is reported in brackets close to the related value: (N) negligible, (VS) very small, (S) small, (M) medium, (L) large, (VL) very large, (H) huge.

	Dataset 1		Dataset 2		Dataset 3	
Pipeline	Corrected p-value	Effect size	Corrected p-value	Effect size	Corrected p-value	Effect size
TSS	1.000	0 (N)	1.000	0 (N)	1.000	0 (N)
CSS	1.000	0 (N)	1.000	0 (N)	1.000	0 (N)
edgeR	1.000	0 (N)	1.000	0 (N)	1.000	0 (N)
DESeq2	1.000	0 (N)	1.000	0 (N)	1.000	0 (N)
GMPR	1.000	0 (N)	1.000	0 (N)	1.000	0 (N)
None_DrImpute	4.00E-24*	1.923 (VL)	1.000	-0.380 (S)	1.000	-0.991 (L)
None_scImpute	2.68E-24*	0.644 (M)	2.40E-14*	2.155 (H)	1.000	-1.097 (L)
None_LLSimpute	1.000	-2.732 (H)	1.000	-1.647 (VL)	1.000	-1.786 (VL)
None_zCompositions_SQ	1.000	-3.335 (H)	1.000	-2.335 (H)	1.000	-1.874 (VL)
None_zCompositions_CZM	1.000	-3.335 (H)	1.000	-2.335 (H)	1.000	-1.874 (VL)
TSS_DrImpute	2.68E-24*	2.113 (H)	1.000	-0.38 (S)	1.000	-1.152 (L)
TSS_scImpute	2.68E-24*	0.645 (M)	2.40E-14*	2.152 (H)	1.000	-1.109 (L)
TSS_LLSimpute	1.000	-2.559 (H)	1.000	-1.736 (VL)	1.000	-1.781 (VL)
TSS_zCompositions_SQ	1.000	-3.335 (H)	1.000	-2.335 (H)	1.000	-1.874 (VL)
TSS_zCompositions_CZM	1.000	-3.335 (H)	1.000	-2.335 (H)	1.000	-1.874 (VL)
CSS_DrImpute	2.68E-24*	2.113 (H)	1.000	-0.38 (S)	1.000	-0.971 (L)
CSS_scImpute	2.68E-24*	0.645 (M)	2.40E-14*	2.152 (H)	1.000	-1.08 (L)
CSS_LLSimpute	1.000	-3.041 (H)	1.000	-1.621 (VL)	1.000	-1.8 (VL)
CSS_zCompositions_SQ	1.000	-3.335 (H)	1.000	-2.335 (H)	1.000	-1.874 (VL)
CSS_zCompositions_CZM	1.000	-3.335 (H)	1.000	-2.335 (H)	1.000	-1.874 (VL)
edgeR_DrImpute	2.68E-24*	2.113 (H)	1.000	-0.38 (S)	1.000	-1.152 (L)
edgeR_scImpute	2.68E-24*	0.645 (M)	2.40E-14*	2.152 (H)	1.000	-1.111 (L)
edgeR_LLSimpute	1.000	-2.978 (H)	1.000	-1.585 (VL)	1.000	-1.816 (VL)
edgeR_zCompositions_SQ	1.000	-3.335 (H)	1.000	-2.335 (H)	1.000	-1.874 (VL)
edgeR_zCompositions_CZM	1.000	-3.335 (H)	1.000	-2.335 (H)	1.000	-1.874 (VL)
DESeq2_DrImpute	2.68E-24*	2.113 (H)	1.000	-0.38 (S)	1.000	-0.991 (L)
DESeq2_scImpute	2.68E-24*	0.645 (M)	2.40E-14*	2.153 (H)	1.000	-1.083 (L)
DESeq2_LLSimpute	1.000	-3.034 (H)	1.000	-1.488 (VL)	1.000	-1.796 (VL)
DESeq2_zCompositions_SQ	1.000	-3.335 (H)	1.000	-2.335 (H)	1.000	-1.874 (VL)
DESeq2_zCompositions_CZM	1.000	-3.335 (H)	1.000	-2.335 (H)	1.000	-1.874 (VL)
GMPR_DrImpute	2.68E-24*	2.113 (H)	1.000	-0.38 (S)	1.000	-0.991 (L)
GMPR_scImpute	2.68E-24*	0.645 (M)	2.40E-14*	2.153 (H)	1.000	-1.076 (L)
GMPR_LLSimpute	1.000	-2.959 (H)	1.000	-1.738 (VL)	1.000	-1.803 (VL)
GMPR_zCompositions_SQ	1.000	-3.335 (H)	1.000	-2.335 (H)	1.000	-1.874 (VL)
GMPR_zCompositions_CZM	1.000	-3.335 (H)	1.000	-2.335 (H)	1.000	-1.874 (VL)

Figure S10. Simulated dataset 1 - Relative error on Pielou alpha diversity indices. (The lower the better). Relative errors are computed between alpha indices value from ground truth data and the corresponding alpha indices values obtained from pre-processed data. Distributions of relative error values that result statistically lower (one-sided paired Mann-Whitney paired U-test, Benjamini-Hochberg correction, significant threshold 0.05) than relative errors calculated on raw or normalized-only data are indicated with the symbol "*", followed by the interpretation of Cohen's d effect size (N: negligible, VS: very small, S: small, M: medium, L: large, VL: very large, H: huge). The vertical dashed line indicates the median relative error on raw or normalized-only data.

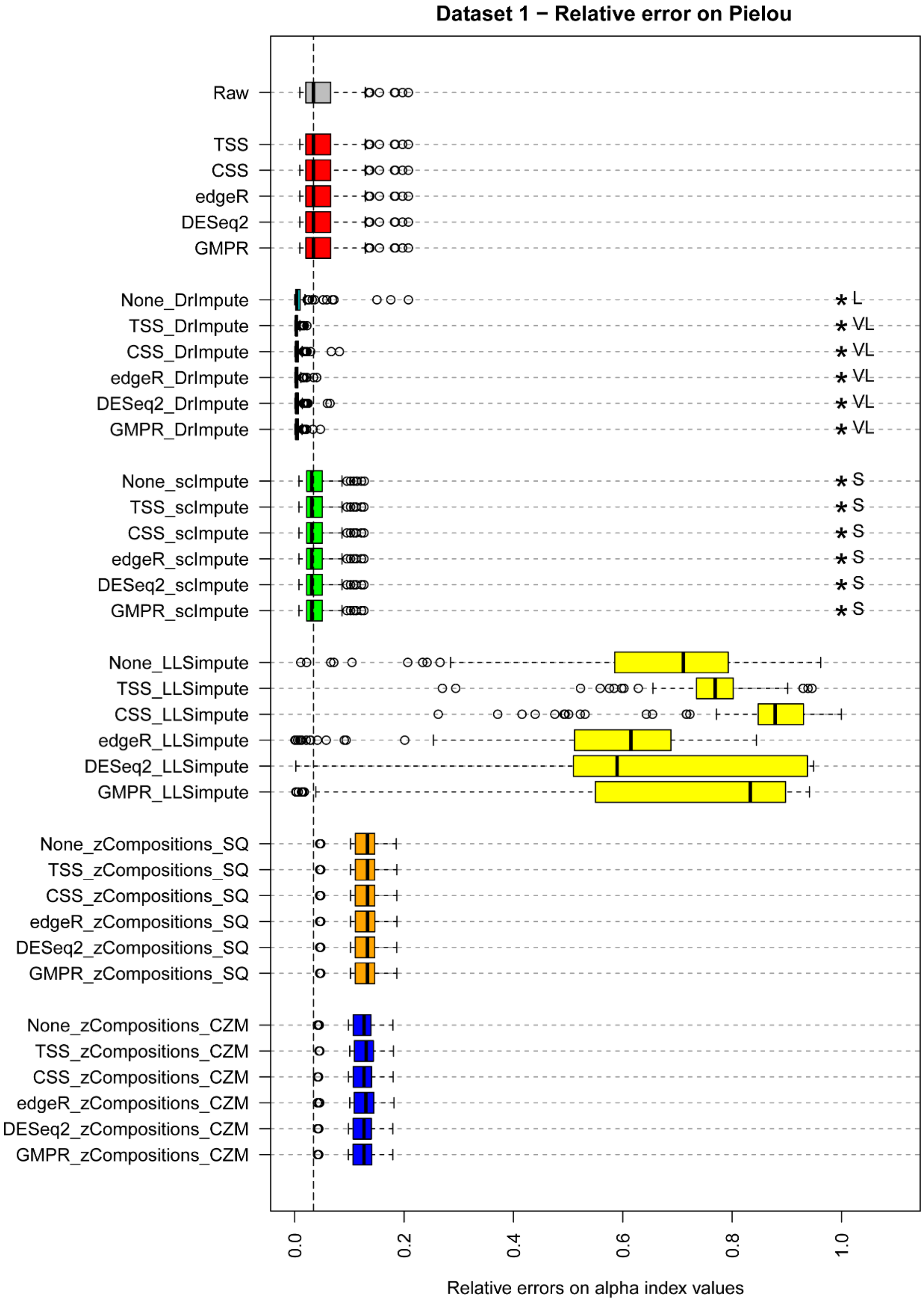

Figure S11. Simulated dataset 2 - Relative error on Pielou alpha diversity indices. (The lower the better). Relative errors are computed between alpha indices value from ground truth data and the corresponding alpha indices values obtained from pre-processed data. Distributions of relative error values that result statistically lower (one-sided paired Mann-Whitney paired U-test, Benjamini-Hochberg correction, significant threshold 0.05) than relative errors calculated on raw or normalized-only data are indicated with the symbol "*", followed by the interpretation of Cohen's d effect size (N: negligible, VS: very small, S: small, M: medium, L: large, VL: very large, H: huge). The vertical dashed line indicates the median relative error on raw or normalized-only data.

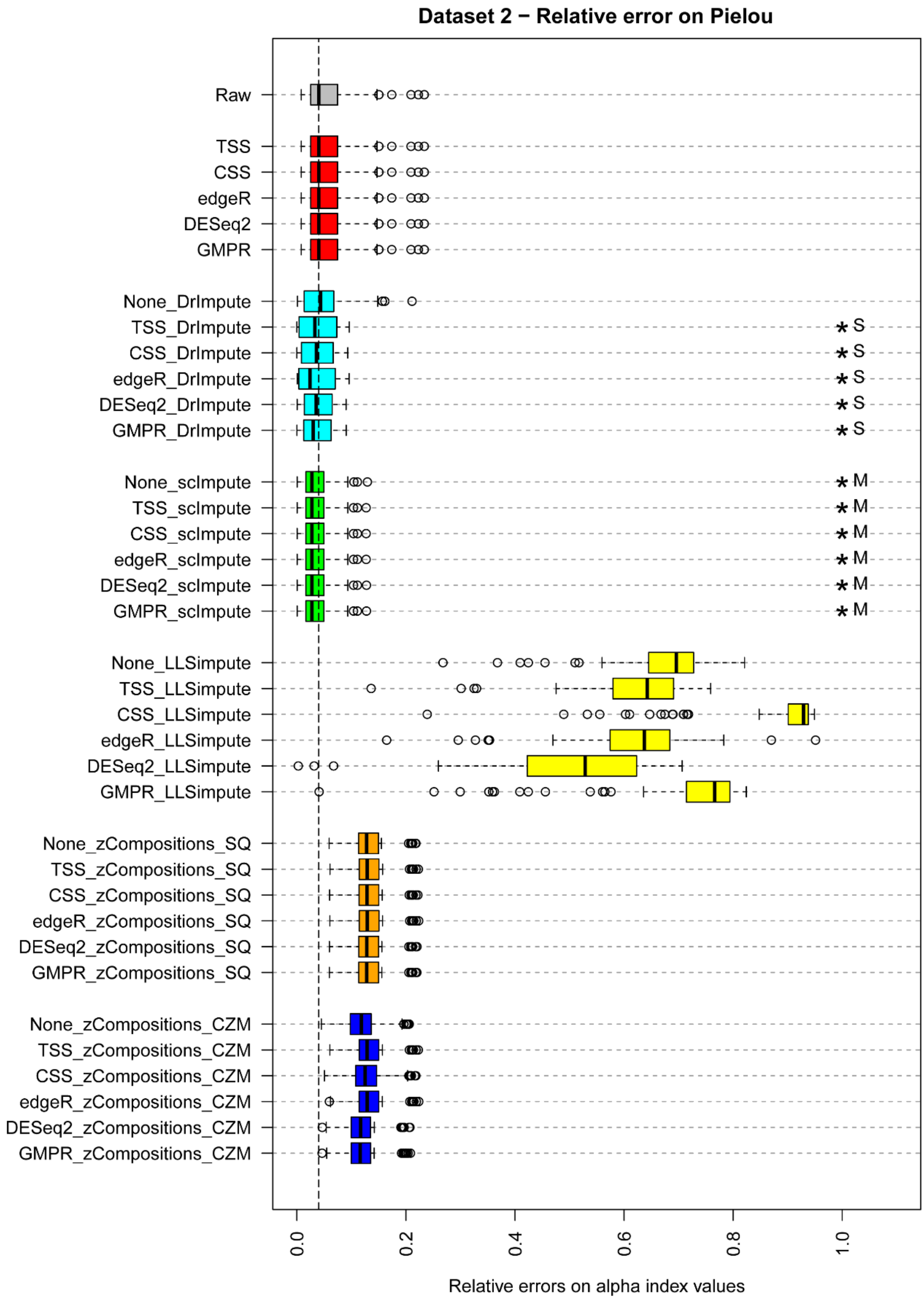

Figure S12. Simulated dataset 3 - Relative error on Pielou alpha diversity indices. (The lower the better). Relative errors are computed between alpha indices value from ground truth data and the corresponding alpha indices values obtained from pre-processed data. Distributions of relative error values that result statistically lower (one-sided paired Mann-Whitney paired U-test, Benjamini-Hochberg correction, significant threshold 0.05) than relative errors calculated on raw or normalized-only data are indicated with the symbol "*", followed by the interpretation of Cohen's d effect size (N: negligible, VS: very small, S: small, M: medium, L: large, VL: very large, H: huge). The vertical dashed line indicates the median relative error on raw or normalized-only data.

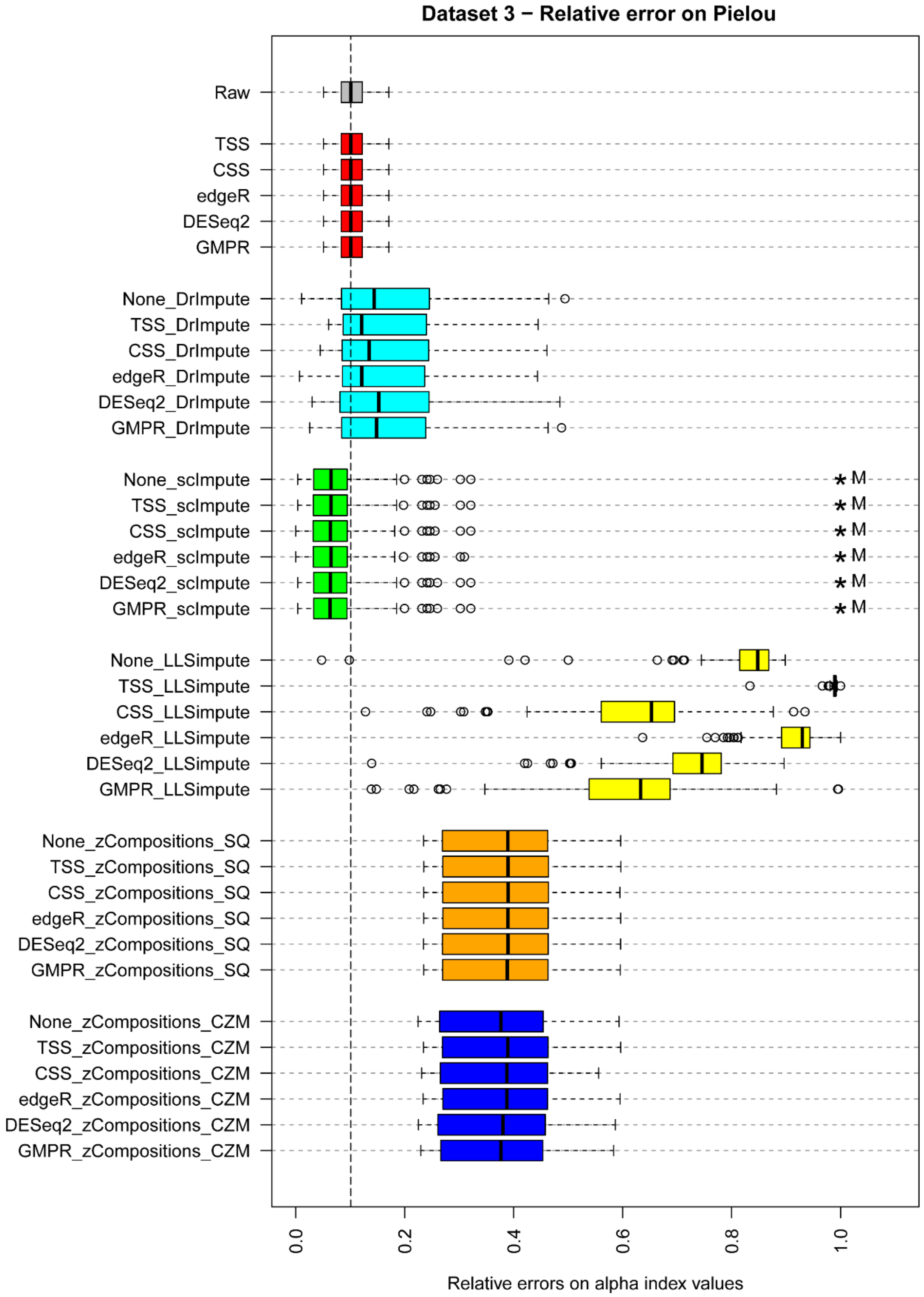

Table S14. Results of statistical analysis on relative error between ground truth and pre-processed data Pielou indices. The analysis aims at identifying pipeline achieving relative error values lower than the ones obtained using raw data (or normalized-only pipelines). For each of the three test datasets and for each pipeline, the corrected p-values (one-sided paired Mann-Whitney paired U-test, Benjamini-Hochberg correction, significant threshold 0.05) and the effect sizes are reported. p-values associated to statistically significant comparisons are indicated with the symbol "*" close to the p-value value. The interpretation of Cohen's d effect size is reported in brackets close to the related value: (N) negligible, (VS) very small, (S) small, (M) medium, (L) large, (VL) very large, (H) huge.

	Dataset 1		Dataset 2		Dataset 3	
Pipeline	Corrected p-value	Effect size	Corrected p-value	Effect size	Corrected p-value	Effect size
TSS	1.000	0 (N)	1.000	0 (N)	1.000	0 (N)
CSS	1.000	0 (N)	1.000	0 (N)	1.000	0 (N)
edgeR	1.000	0 (N)	1.000	0 (N)	1.000	0 (N)
DESeq2	1.000	0 (N)	1.000	0 (N)	1.000	0 (N)
GMPR	1.000	0 (N)	1.000	0 (N)	1.000	0 (N)
None_DrImpute	5.81E-24*	1.065 (L)	0.073	0.222 (S)	1.000	-0.838 (L)
None_scImpute	7.34E-10*	0.267 (S)	2.88E-06*	0.57 (M)	1.20E-06*	0.571 (M)
None_LLSimpute	1.000	-4.447 (H)	1.000	-8.034 (H)	1.000	-7.887 (H)
None_zCompositions_SQ	1.000	-2.221 (H)	1.000	-1.661 (VL)	1.000	-3.706 (H)
None_zCompositions_CZM	1.000	-2.118 (H)	1.000	-1.392 (VL)	1.000	-3.598 (H)
TSS_DrImpute	4.28E-24*	1.546 (VL)	0.029*	0.399 (S)	1.000	-0.842 (L)
TSS_scImpute	7.34E-10*	0.268 (S)	2.88E-06*	0.572 (M)	1.20E-06*	0.57 (M)
TSS_LLSimpute	1.000	-10.443 (H)	1.000	-6.844 (H)	1.000	-39.563 (H)
TSS_zCompositions_SQ	1.000	-2.222 (H)	1.000	-1.682 (VL)	1.000	-3.714 (H)
TSS_zCompositions_CZM	1.000	-2.173 (H)	1.000	-1.676 (VL)	1.000	-3.706 (H)
CSS_DrImpute	4.28E-24*	1.441 (VL)	0.018*	0.46 (S)	1.000	-0.869 (L)
CSS_scImpute	7.34E-10*	0.268 (S)	2.88E-06*	0.572 (M)	1.20E-06*	0.578 (M)
CSS_LLSimpute	1.000	-8.109 (H)	1.000	-7.995 (H)	1.000	-5.474 (H)
CSS_zCompositions_SQ	1.000	-2.221 (H)	1.000	-1.673 (VL)	1.000	-3.714 (H)
CSS_zCompositions_CZM	1.000	-2.102 (H)	1.000	-1.565 (VL)	1.000	-3.705 (H)
edgeR_DrImpute	4.28E-24*	1.52 (VL)	0.019*	0.447 (S)	1.000	-0.819 (L)
edgeR_scImpute	7.34E-10*	0.268 (S)	2.88E-06*	0.572 (M)	1.20E-06*	0.584 (M)
edgeR_LLSimpute	1.000	-3.177 (H)	1.000	-6.333 (H)	1.000	-17.734 (H)
edgeR_zCompositions_SQ	1.000	-2.222 (H)	1.000	-1.681 (VL)	1.000	-3.714 (H)
edgeR_zCompositions_CZM	1.000	-2.164 (H)	1.000	-1.672 (VL)	1.000	-3.706 (H)
DESeq2_DrImpute	4.28E-24*	1.462 (VL)	0.015*	0.449 (S)	1.000	-0.858 (L)
DESeq2_scImpute	7.34E-10*	0.268 (S)	2.88E-06*	0.572 (M)	1.20E-06*	0.578 (M)
DESeq2_LLSimpute	1.000	-2.97 (H)	1.000	-3.994 (H)	1.000	-8.191 (H)
DESeq2_zCompositions_SQ	1.000	-2.221 (H)	1.000	-1.665 (VL)	1.000	-3.707 (H)
DESeq2_zCompositions_CZM	1.000	-2.103 (H)	1.000	-1.391 (VL)	1.000	-3.581 (H)
GMPR_DrImpute	4.28E-24*	1.502 (VL)	0.011*	0.476 (S)	1.000	-0.873 (L)
GMPR_scImpute	7.34E-10*	0.268 (S)	2.88E-06*	0.571 (M)	1.20E-06*	0.58 (M)
GMPR_LLSimpute	1.000	-3.238 (H)	1.000	-5.705 (H)	1.000	-4.446 (H)
GMPR_zCompositions_SQ	1.000	-2.221 (H)	1.000	-1.664 (VL)	1.000	-3.712 (H)
GMPR_zCompositions_CZM	1.000	-2.099 (H)	1.000	-1.387 (VL)	1.000	-3.691 (H)

Table S15. Simulated Dataset 1 - Results of the pipelines on alpha richness index. In the "Wrong comparisons" column is reported, in decreasing order for each index, the number of group-group comparisons not agreeing with the ground truth. The percentage over the total number of comparisons is also reported in the column "%".

Richness		
**Pipeline**	**Wrong comparison**	**%**
TSS_DrImpute	2	2.20
CSS_DrImpute	2	2.20
edgeR_DrImpute	2	2.20
DESeq2_DrImpute	2	2.20
GMPR_DrImpute	2	2.20
None_DrImpute	3	3.30
None_scImpute	21	23.08
TSS_scImpute	21	23.08
CSS_scImpute	21	23.08
edgeR_scImpute	21	23.08
DESeq2_scImpute	21	23.08
GMPR_scImpute	21	23.08
None_LLSimpute	26	28.57
CSS_LLSimpute	29	31.87
edgeR_LLSimpute	31	34.07
Raw	34	37.36
TSS	34	37.36
CSS	34	37.36
edgeR	34	37.36
DESeq2	34	37.36
GMPR	34	37.36
DESeq2_LLSimpute	34	37.36
GMPR_LLSimpute	41	45.05
TSS_LLSimpute	44	48.35
None_zCompositions_SQ	91	100.00
None_zCompositions_CZM	91	100.00
TSS_zCompositions_SQ	91	100.00
TSS_zCompositions_CZM	91	100.00
CSS_zCompositions_SQ	91	100.00
CSS_zCompositions_CZM	91	100.00
edgeR_zCompositions_SQ	91	100.00
edgeR_zCompositions_CZM	91	100.00
DESeq2_zCompositions_SQ	91	100.00
DESeq2_zCompositions_CZM	91	100.00
GMPR_zCompositions_SQ	91	100.00
GMPR_zCompositions_CZM	91	100.00

Table S16. Simulated Dataset 2 - Results of the pipelines on alpha richness index. In the "Wrong comparisons" column is reported, in decreasing order for each index, the number of group-group comparisons not agreeing with the ground truth. The percentage over the total number of comparisons is also reported in the column "%".

Richness		
**Pipeline**	**Wrong comparison**	**%**
None_scImpute	3	10.71
TSS_scImpute	3	10.71
CSS_scImpute	3	10.71
edgeR_scImpute	3	10.71
DESeq2_scImpute	3	10.71
GMPR_scImpute	3	10.71
Raw	11	39.29
TSS	11	39.29
CSS	11	39.29
edgeR	11	39.29
DESeq2	11	39.29
GMPR	11	39.29
TSS_LLSimpute	14	50.00
CSS_LLSimpute	15	53.57
edgeR_LLSimpute	15	53.57
None_LLSimpute	16	57.14
DESeq2_LLSimpute	17	60.71
GMPR_LLSimpute	17	60.71
None_DrImpute	18	64.29
TSS_DrImpute	18	64.29
CSS_DrImpute	18	64.29
edgeR_DrImpute	18	64.29
DESeq2_DrImpute	18	64.29
GMPR_DrImpute	18	64.29
None_zCompositions_SQ	26	92.86
None_zCompositions_CZM	26	92.86
TSS_zCompositions_SQ	26	92.86
TSS_zCompositions_CZM	26	92.86
CSS_zCompositions_SQ	26	92.86
CSS_zCompositions_CZM	26	92.86
edgeR_zCompositions_SQ	26	92.86
edgeR_zCompositions_CZM	26	92.86
DESeq2_zCompositions_SQ	26	92.86
DESeq2_zCompositions_CZM	26	92.86
GMPR_zCompositions_SQ	26	92.86
GMPR_zCompositions_CZM	26	92.86

Table S17. Simulated Dataset 3 - Results of the pipelines on alpha richness index. In the "Wrong comparisons" column is reported, in decreasing order for each index, the number of group-group comparisons not agreeing with the ground truth. The percentage over the total number of comparisons is also reported in the column "%".

Richness		
**Pipeline**	**Wrong comparison**	**%**
Raw	4	6.06
TSS	4	6.06
CSS	4	6.06
edgeR	4	6.06
DESeq2	4	6.06
GMPR	4	6.06
None_scImpute	4	6.06
TSS_scImpute	4	6.06
CSS_scImpute	4	6.06
edgeR_scImpute	4	6.06
DESeq2_scImpute	4	6.06
GMPR_scImpute	4	6.06
TSS_DrImpute	18	27.27
edgeR_DrImpute	18	27.27
None_DrImpute	23	34.85
CSS_DrImpute	23	34.85
DESeq2_DrImpute	23	34.85
GMPR_DrImpute	23	34.85
GMPR_LLSimpute	56	84.85
TSS_LLSimpute	59	89.39
CSS_LLSimpute	61	92.42
edgeR_LLSimpute	61	92.42
None_LLSimpute	63	95.45
None_zCompositions_SQ	63	95.45
None_zCompositions_CZM	63	95.45
TSS_zCompositions_SQ	63	95.45
TSS_zCompositions_CZM	63	95.45
CSS_zCompositions_SQ	63	95.45
CSS_zCompositions_CZM	63	95.45
edgeR_zCompositions_SQ	63	95.45
edgeR_zCompositions_CZM	63	95.45
DESeq2_zCompositions_SQ	63	95.45
DESeq2_zCompositions_CZM	63	95.45
GMPR_zCompositions_SQ	63	95.45
GMPR_zCompositions_CZM	63	95.45
DESeq2_LLSimpute	64	96.97

Table S18. Simulated Dataset 1 - Results of the pipelines on alpha evenness index. In the "Wrong comparisons" column is reported, in decreasing order for each index, the number of group-group comparisons not agreeing with the ground truth. The percentage over the total number of comparisons is also reported in the column "%".

Pielou		
**Pipeline**	**Wrong comparison**	**%**
TSS_DrImpute	2	2.20
GMPR_DrImpute	2	2.20
edgeR_DrImpute	3	3.30
CSS_DrImpute	4	4.40
DESeq2_DrImpute	4	4.40
None_scImpute	5	5.49
TSS_scImpute	5	5.49
CSS_scImpute	5	5.49
edgeR_scImpute	5	5.49
DESeq2_scImpute	5	5.49
GMPR_scImpute	5	5.49
None_DrImpute	8	8.79
Raw	9	9.89
TSS	9	9.89
CSS	9	9.89
edgeR	9	9.89
DESeq2	9	9.89
GMPR	9	9.89
None_zCompositions_SQ	12	13.19
TSS_zCompositions_SQ	12	13.19
TSS_zCompositions_CZM	12	13.19
CSS_zCompositions_SQ	12	13.19
CSS_zCompositions_CZM	12	13.19
edgeR_zCompositions_SQ	12	13.19
DESeq2_zCompositions_SQ	12	13.19
DESeq2_zCompositions_CZM	12	13.19
GMPR_zCompositions_SQ	12	13.19
GMPR_zCompositions_CZM	12	13.19
None_zCompositions_CZM	13	14.29
edgeR_zCompositions_CZM	13	14.29
None_LLSimpute	41	45.05
GMPR_LLSimpute	51	56.04
DESeq2_LLSimpute	53	58.24
CSS_LLSimpute	55	60.44
edgeR_LLSimpute	56	61.54
TSS_LLSimpute	57	62.64

Table S19. Simulated Dataset 2 - Results of the pipelines on alpha evenness index. In the "Wrong comparisons" column is reported, in decreasing order for each index, the number of group-group comparisons not agreeing with the ground truth. The percentage over the total number of comparisons is also reported in the column "%".

Pielou		
**Pipeline**	**Wrong comparison**	**%**
Raw	0	0.00
TSS	0	0.00
CSS	0	0.00
edgeR	0	0.00
DESeq2	0	0.00
GMPR	0	0.00
None_scImpute	0	0.00
TSS_scImpute	0	0.00
CSS_scImpute	0	0.00
edgeR_scImpute	0	0.00
DESeq2_scImpute	0	0.00
GMPR_scImpute	0	0.00
None_zCompositions_SQ	1	3.57
None_zCompositions_CZM	1	3.57
TSS_zCompositions_SQ	1	3.57
TSS_zCompositions_CZM	1	3.57
CSS_zCompositions_SQ	1	3.57
CSS_zCompositions_CZM	1	3.57
edgeR_zCompositions_SQ	1	3.57
edgeR_zCompositions_CZM	1	3.57
DESeq2_zCompositions_SQ	1	3.57
DESeq2_zCompositions_CZM	1	3.57
GMPR_zCompositions_SQ	1	3.57
GMPR_zCompositions_CZM	1	3.57
None_DrImpute	3	10.71
edgeR_DrImpute	3	10.71
TSS_DrImpute	4	14.29
DESeq2_DrImpute	4	14.29
CSS_DrImpute	5	17.86
GMPR_DrImpute	5	17.86
None_LLSimpute	15	53.57
CSS_LLSimpute	15	53.57
edgeR_LLSimpute	15	53.57
DESeq2_LLSimpute	19	67.86
TSS_LLSimpute	22	78.57
GMPR_LLSimpute	23	82.14

Table S20. Simulated Dataset 3 - Results of the pipelines on alpha evenness index. In the "Wrong comparisons" column is reported, in decreasing order for each index, the number of group-group comparisons not agreeing with the ground truth. The percentage over the total number of comparisons is also reported in the column "%".

Pielou		
**Pipeline**	**Wrong comparison**	**%**
Raw	3	4.55
TSS	3	4.55
CSS	3	4.55
edgeR	3	4.55
DESeq2	3	4.55
GMPR	3	4.55
TSS_scImpute	11	16.67
edgeR_scImpute	11	16.67
None_scImpute	12	18.18
DESeq2_scImpute	12	18.18
GMPR_scImpute	12	18.18
CSS_scImpute	13	19.70
TSS_DrImpute	22	33.33
edgeR_DrImpute	22	33.33
CSS_DrImpute	24	36.36
GMPR_DrImpute	24	36.36
None_DrImpute	25	37.88
DESeq2_DrImpute	25	37.88
GMPR_zCompositions_CZM	25	37.88
None_zCompositions_SQ	26	39.39
None_zCompositions_CZM	26	39.39
TSS_zCompositions_SQ	26	39.39
TSS_zCompositions_CZM	26	39.39
CSS_zCompositions_SQ	26	39.39
CSS_zCompositions_CZM	26	39.39
edgeR_zCompositions_SQ	26	39.39
edgeR_zCompositions_CZM	26	39.39
DESeq2_zCompositions_SQ	26	39.39
DESeq2_zCompositions_CZM	26	39.39
GMPR_zCompositions_SQ	26	39.39
edgeR_LLSimpute	40	60.61
TSS_LLSimpute	41	62.12
GMPR_LLSimpute	41	62.12
DESeq2_LLSimpute	42	63.64
CSS_LLSimpute	48	72.73
None_LLSimpute	55	83.33

Figure S13. Simulated Dataset 1 – Beta diversity (Bray-Curtis dissimilarity). For each pipeline, the plots show the Non-metric Multidimensional Scaling (NMDS) dimensionality reduction on Bray-Curtis dissimilarity. The top-left plot shows the true structure of the data in terms of beta diversity. The remaining plots show the beta diversity obtained on the raw data and the 35 pre-processing pipelines outputs. Plots are displayed in a grid: imputation methods on the rows, normalization methods on the columns. The colours encode the different groups (i.e. experimental conditions) within the datasets.

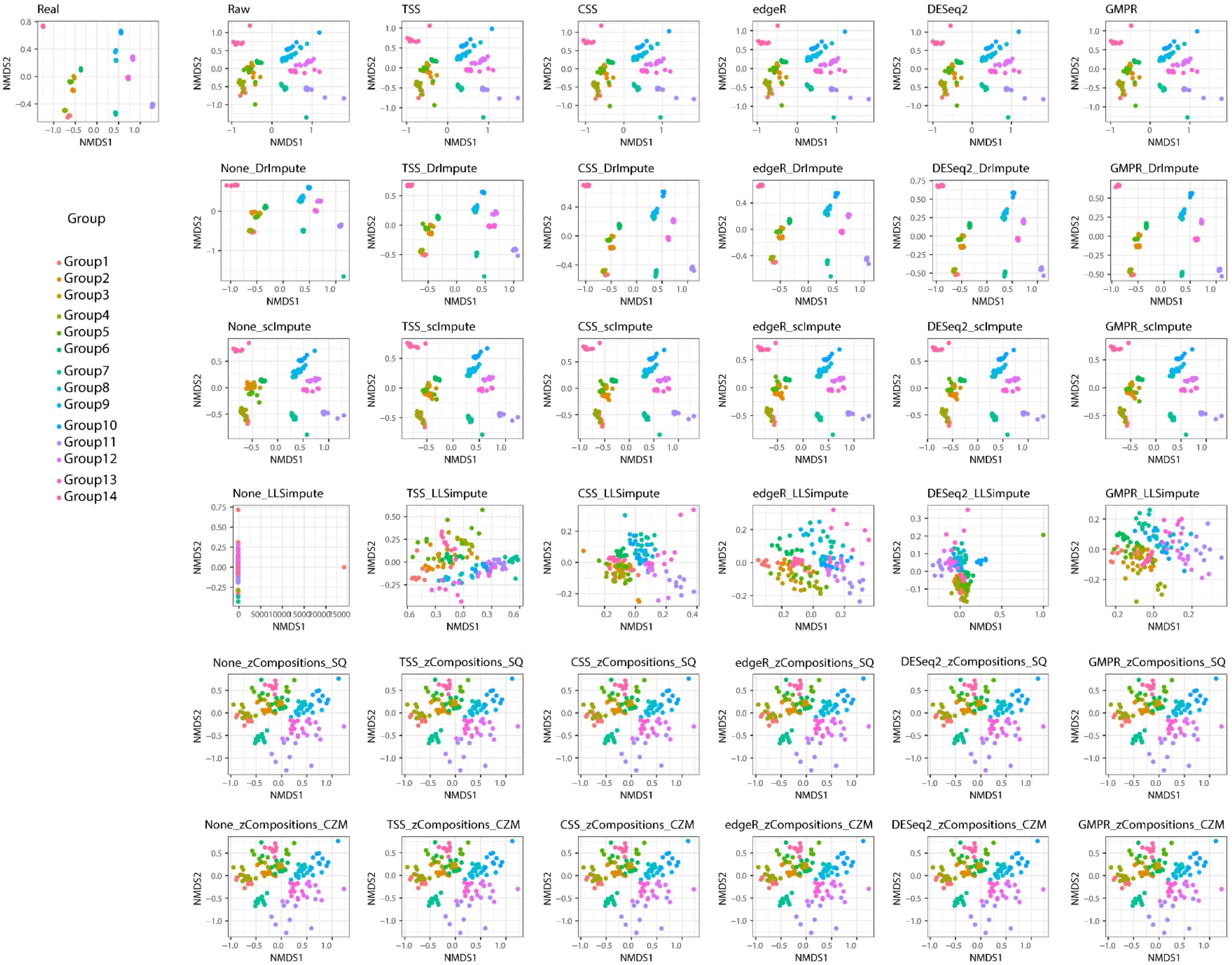

Figure S14. Simulated Dataset 2 – Beta diversity (Bray-Curtis dissimilarity). For each pipeline, the plots show the Non-metric Multidimensional Scaling (NMDS) dimensionality reduction on Bray-Curtis dissimilarity. The top-left plot shows the true structure of the data in terms of beta diversity. The remaining plots show the beta diversity obtained on the raw data and the 35 pre-processing pipelines outputs. Plots are displayed in a grid: imputation methods on the rows, normalization methods on the columns. The colours encode the different groups (i.e. experimental conditions) within the datasets.

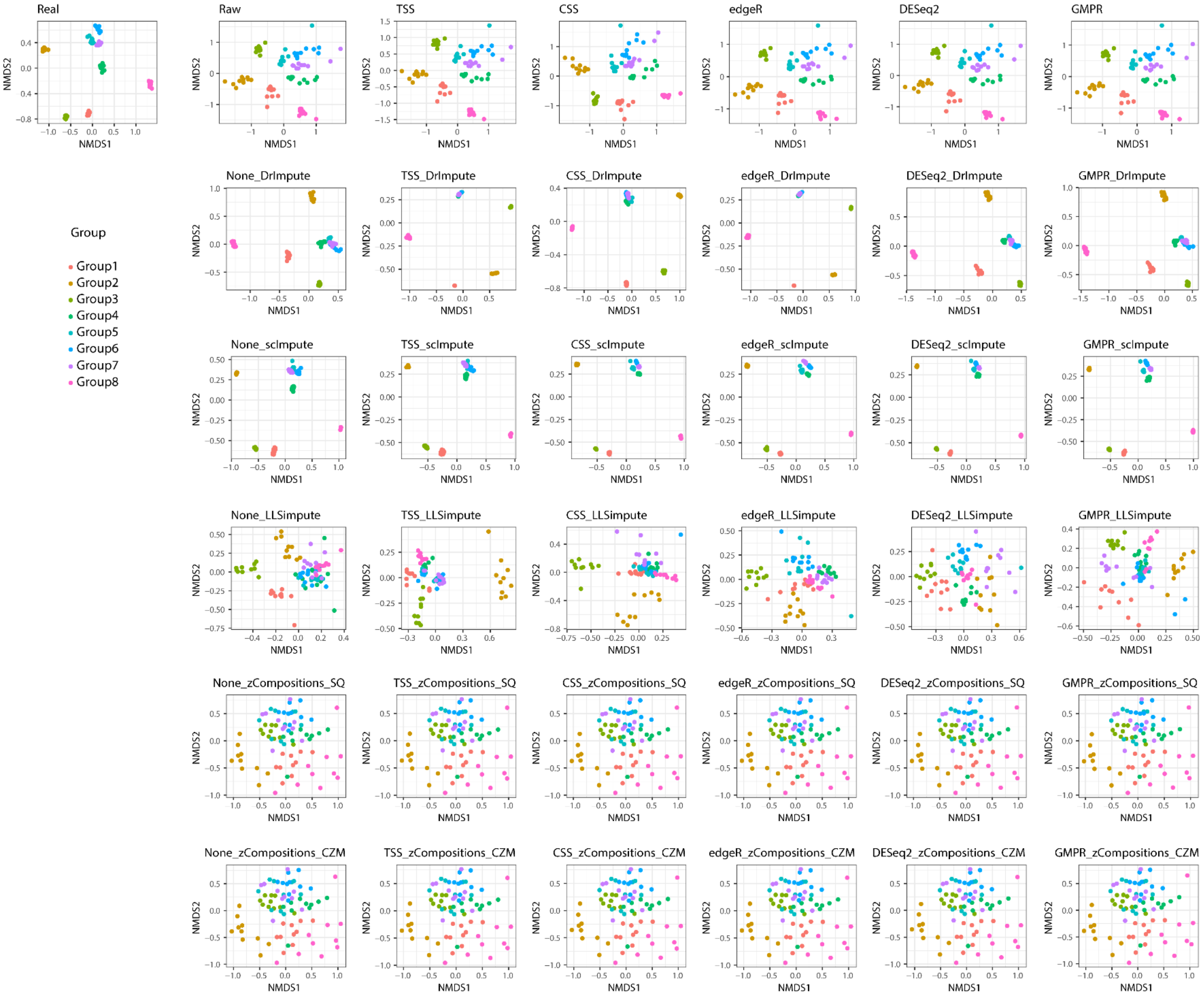

Figure S15. Simulated Dataset 3 – Beta diversity (Bray-Curtis dissimilarity). For each pipeline, the plots show the Non-metric Multidimensional Scaling (NMDS) dimensionality reduction on Bray-Curtis dissimilarity. The top-left plot shows the true structure of the data in terms of beta diversity. The remaining plots show the beta diversity obtained on the raw data and the 35 pre-processing pipelines outputs. Plots are displayed in a grid: imputation methods on the rows, normalization methods on the columns. The colours encode the different groups (i.e. experimental conditions) within the datasets.

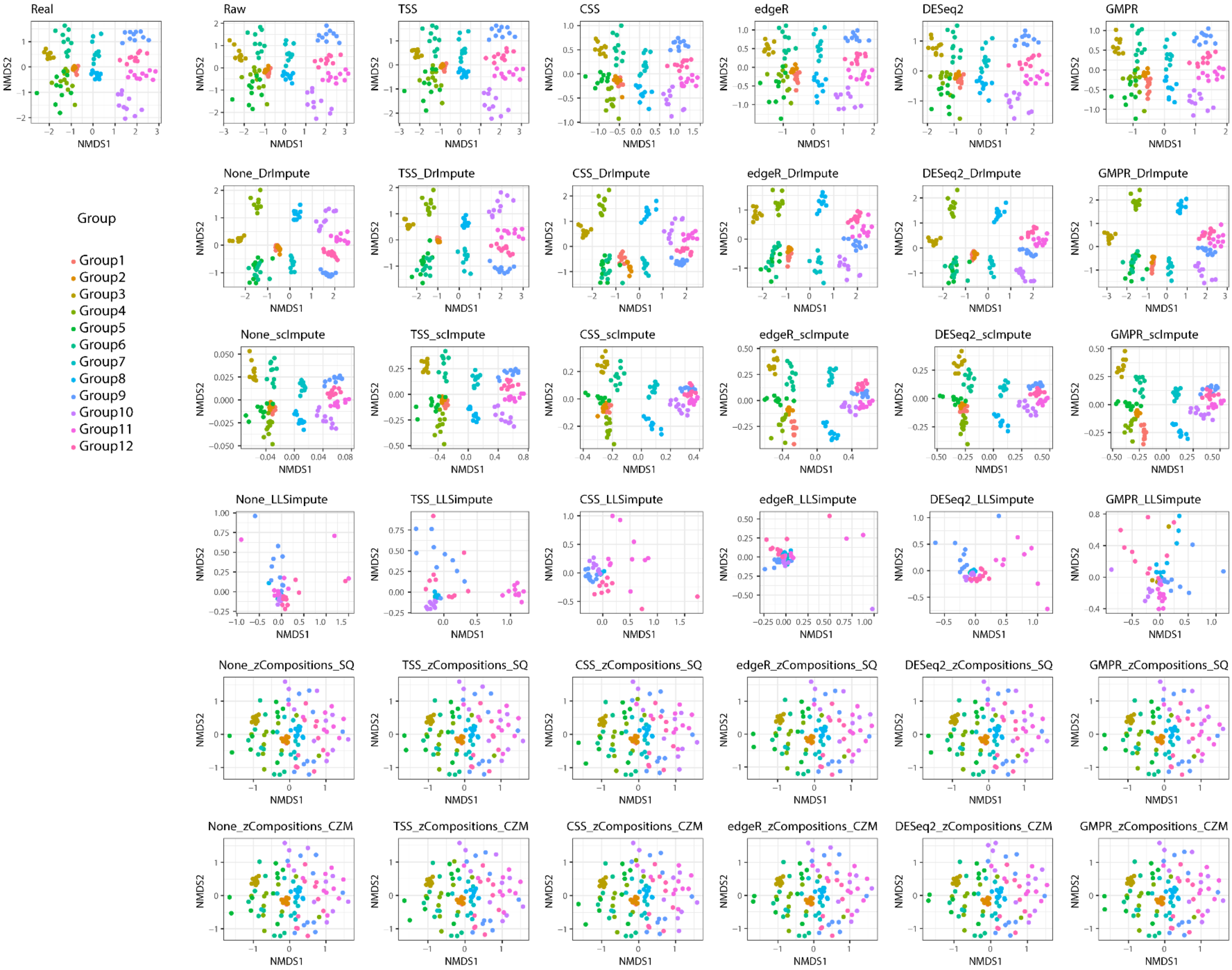

Figure S16. Simulated Dataset 1 – Beta diversity (Whittaker dissimilarity). For each pipeline, the heatmaps show the beta diversity values computed from each pair of samples within the dataset. The top-left heatmap shows the true structure of the data in terms of beta diversity. The remaining heatmaps show the beta diversity obtained by raw data and the 35 pre-processing pipelines outputs. Heatmaps are displayed in a grid: imputation methods on the rows, normalization methods on the columns.

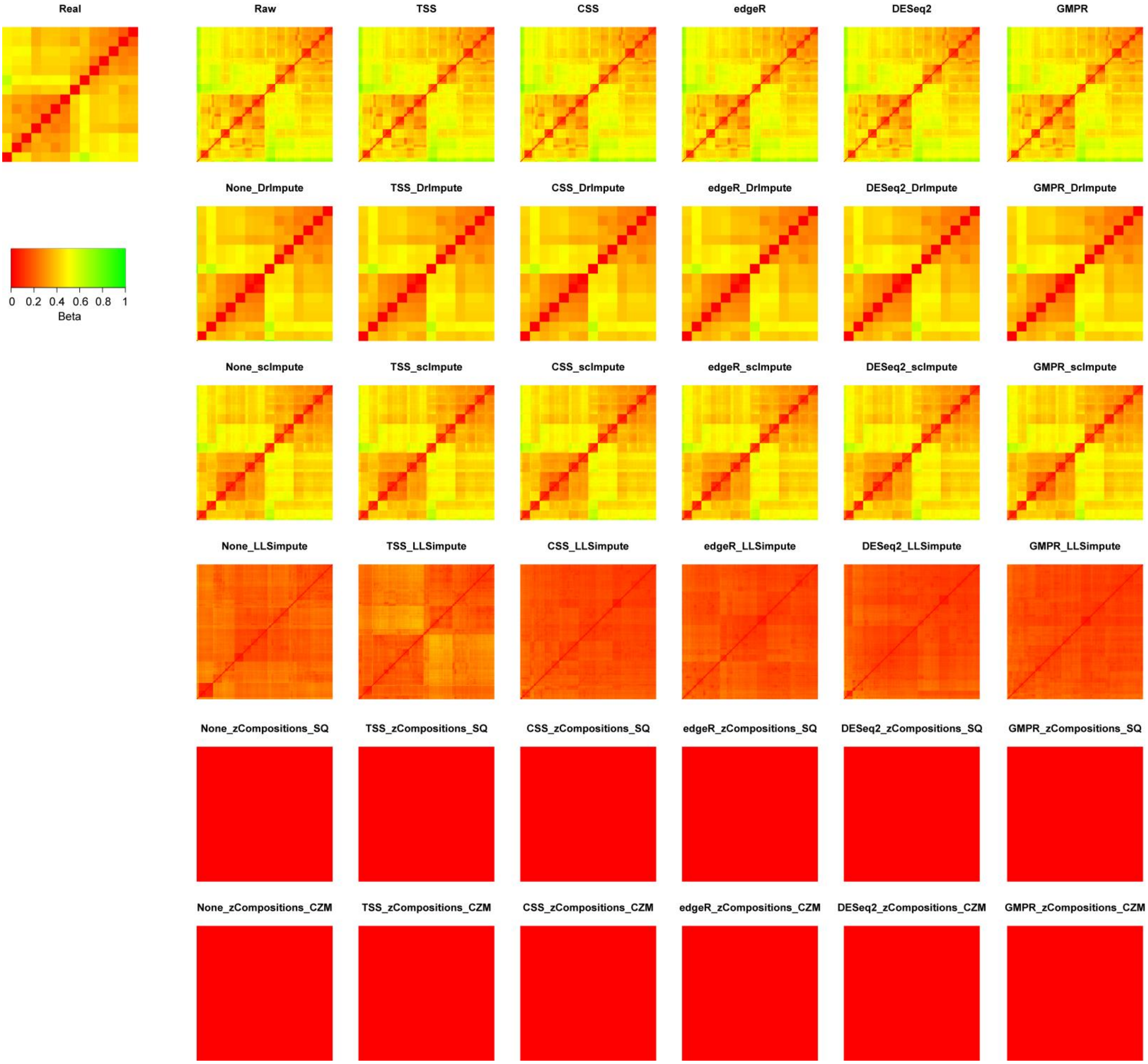

Figure S17. Simulated Dataset 2 – Beta diversity (Whittaker dissimilarity). For each pipeline, the heatmaps show the beta diversity values computed from each pair of samples within the dataset. The top-left heatmap shows the true structure of the data in terms of beta diversity. The remaining heatmaps show the beta diversity obtained by raw data and the 35 pre-processing pipelines outputs. Heatmaps are displayed in a grid: imputation methods on the rows, normalization methods on the columns.

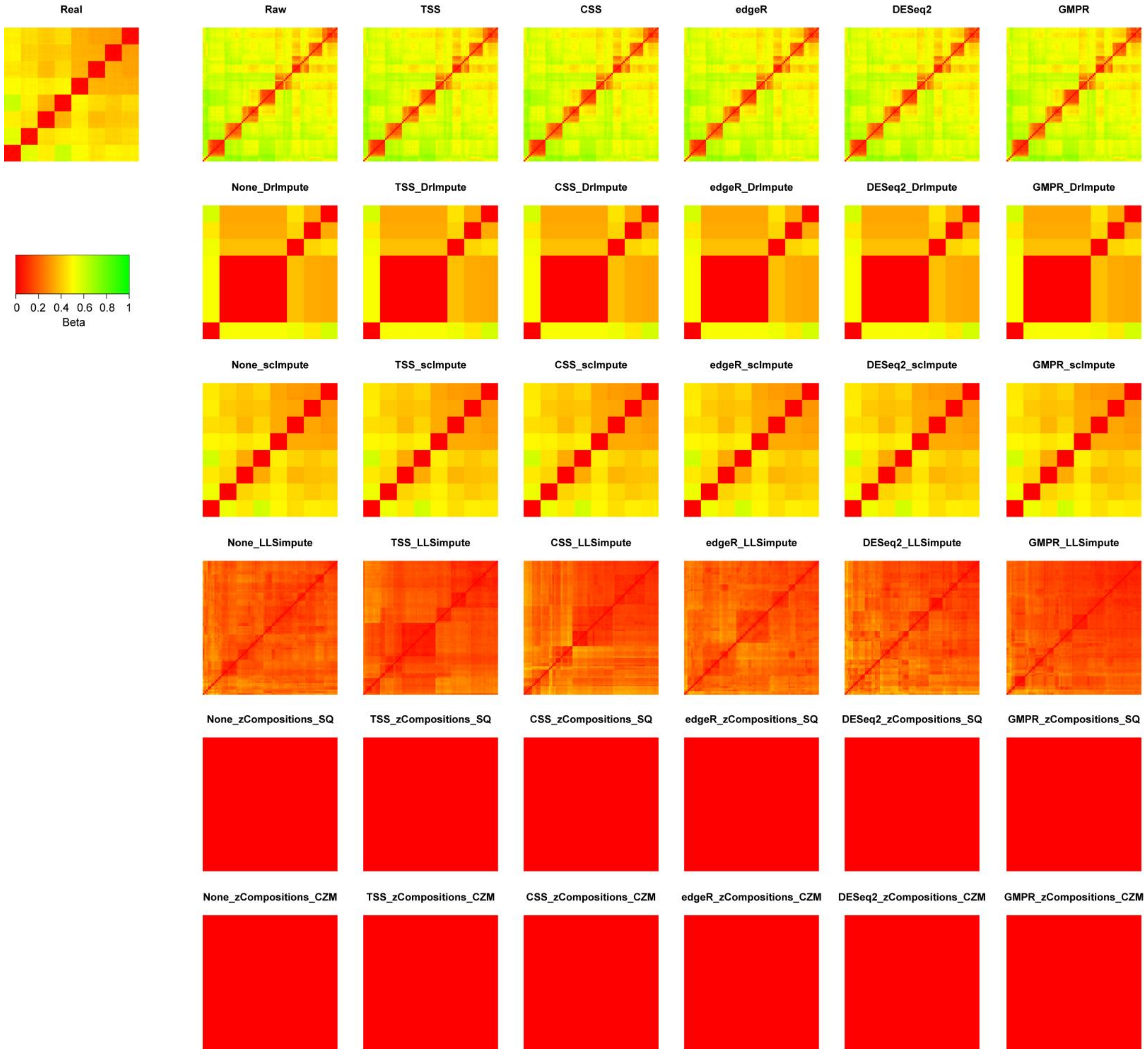

Figure S18. Simulated Dataset 3 – Beta diversity. **Beta diversity (Whittaker dissimilarity)**. For each pipeline, the heatmaps show the beta diversity values computed from each pair of samples within the dataset. The top-left heatmap shows the true structure of the data in terms of beta diversity. The remaining heatmaps show the beta diversity obtained by raw data and the 35 pre-processing pipelines outputs. Heatmaps are displayed in a grid: imputation methods on the rows, normalization methods on the columns.

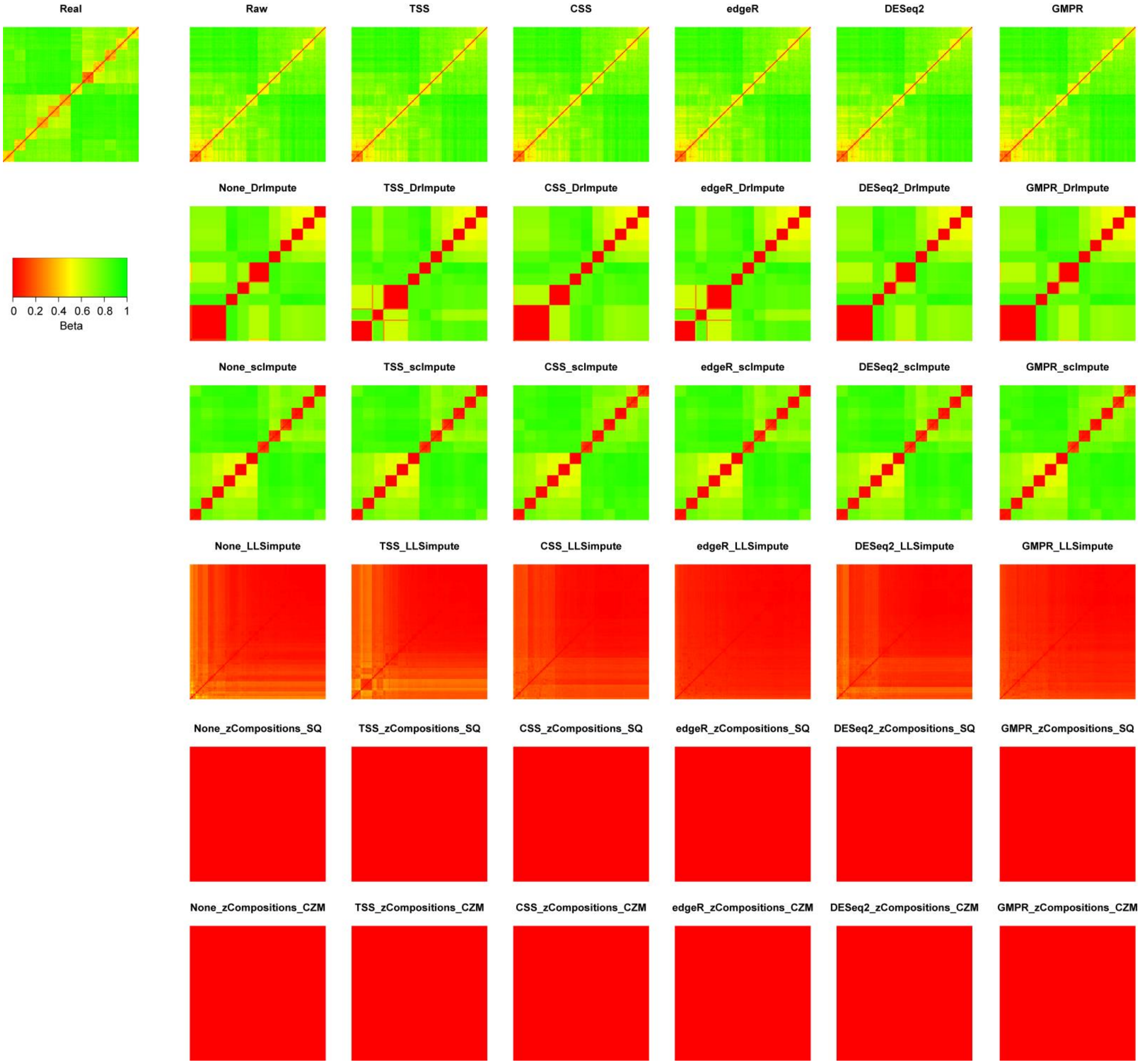

Differential abundance analysis

Figure S19. Simulated Dataset 1 – Jaccard index on differentially abundant species. For each pipeline, Jaccard indices were computed between species identified as differentially abundant (DA) in pre-processed data and DA species in ground truth data. For each pipeline, the boxplots show the distribution of Jaccard indices. Distributions of Jaccard index values that result statistically larger (one-sided Mann-Whitney paired U-test, Benjamini-Hochberg correction, significant threshold 0.05) than Jaccard index values calculated between DA species identified in raw data and DA species in ground truth data are indicated with the symbol "*", followed by the interpretation of Cohen's d effect size (N: negligible, VS: very small, S: small, M: medium, L: large, VL: very large, H: huge). The vertical dashed line indicates the median Jaccard index value of raw data.

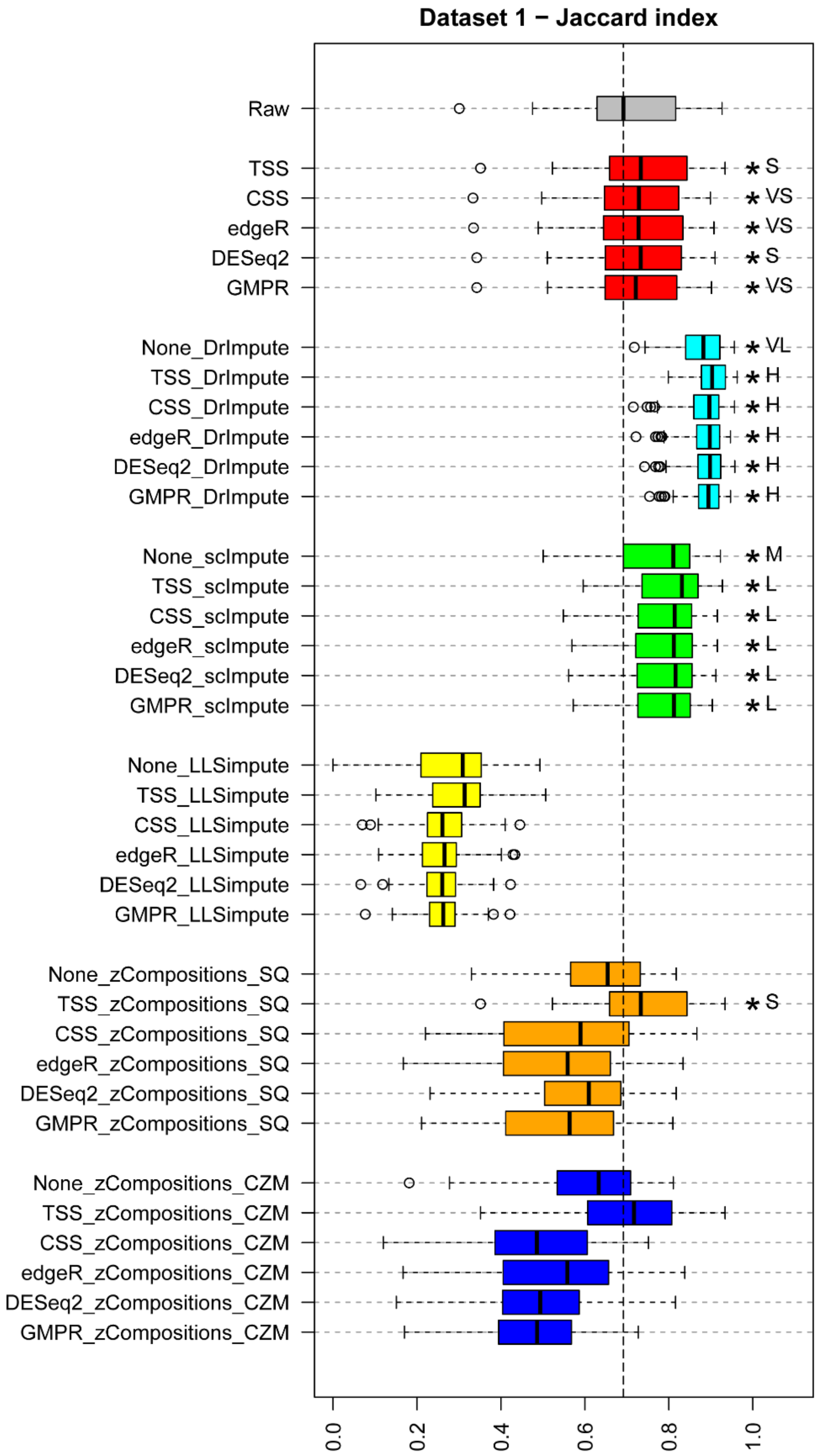

Figure S20. Simulated Dataset 2 – Jaccard index on differentially abundant species. For each pipeline, Jaccard indices were computed between species identified as differentially abundant (DA) in pre-processed data and DA species in ground truth data. For each pipeline, the boxplots show the distribution of Jaccard indices. Distributions of Jaccard index values that result statistically larger (one-sided Mann-Whitney paired U-test, Benjamini-Hochberg correction, significant threshold 0.05) than Jaccard index values calculated between DA species identified in raw data and DA species in ground truth data are indicated with the symbol "*", followed by the interpretation of Cohen's d effect size (N: negligible, VS: very small, S: small, M: medium, L: large, VL: very large, H: huge). The vertical dashed line indicates the median Jaccard index value of raw data.

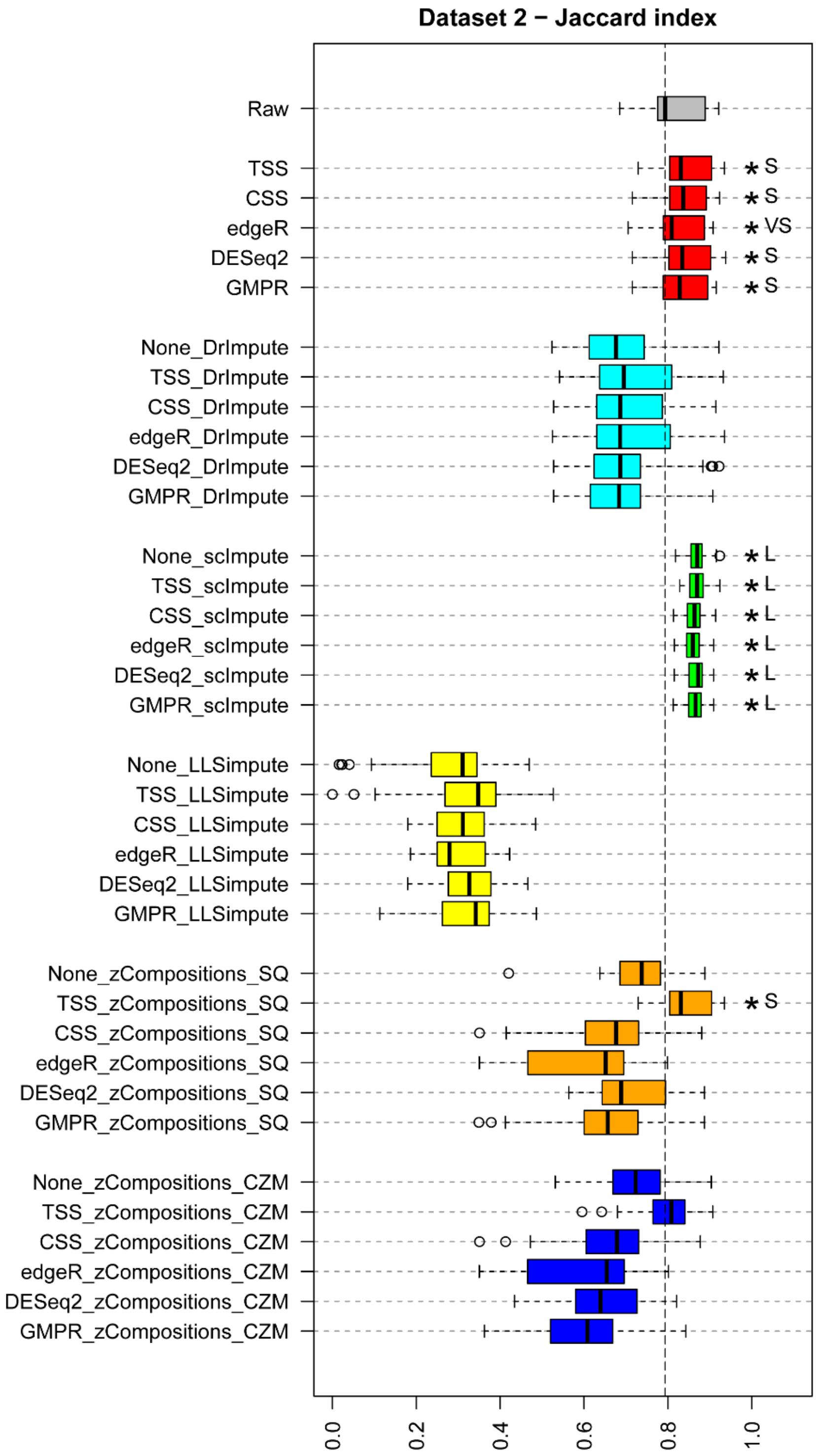

Figure S21. Simulated Dataset 3 – Jaccard index on differentially abundant species. For each pipeline, Jaccard indices were computed between species identified as differentially abundant (DA) in pre-processed data and DA species in ground truth data. For each pipeline, the boxplots show the distribution of Jaccard indices. Distributions of Jaccard index values that result statistically larger (one-sided Mann-Whitney paired U-test, Benjamini-Hochberg correction, significant threshold 0.05) than Jaccard index values calculated between DA species identified in raw data and DA species in ground truth data are indicated with the symbol "*", followed by the interpretation of Cohen's d effect size (N: negligible, VS: very small, S: small, M: medium, L: large, VL: very large, H: huge). The vertical dashed line indicates the median Jaccard index value of raw data.

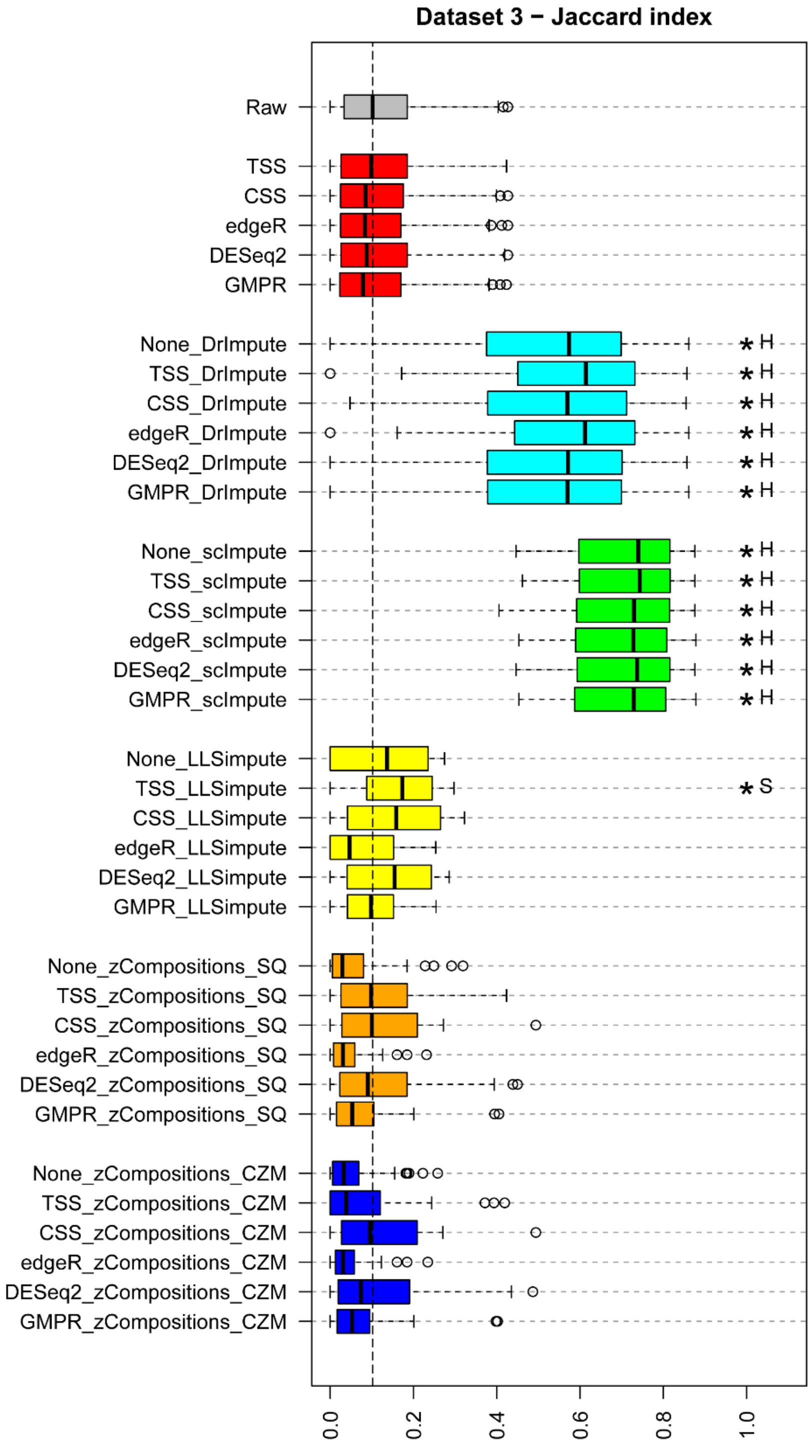

Table S21. Results of statistical analysis on Jaccard index on differentially abundant species. For each pipeline and test dataset, Jaccard indices were computed between species identified as differentially abundant (DA) in pre-processed data and DA species in ground truth data. Similarly, Jaccard indices were computed between species identified as differentially abundant (DA) in raw data and DA species in ground truth data. For each test dataset and for each pipeline, a one-sided Mann-Whitney paired U-test (Benjamini-Hochberg correction, significant threshold 0.05) was performed to test whether Jaccard indices from pre-processed data are larger than Jaccard indices from raw data. Corrected p-values and effect sizes are reported in the table. p-values associated to statistically significant comparisons are indicated with the symbol "*" close to the p-value value. The interpretation of Cohen's d effect size is reported in brackets close to the related value: (N) negligible, (VS) very small, (S) small, (M) medium, (L) large, (VL) very large, (H) huge.

	Dataset 1		Dataset 2		Dataset 3	
Pipeline	Corrected p-value	Effect size	Corrected p-value	Effect size	Corrected p-value	Effect size
TSS	3.49E-16*	-0.336 (S)	6.52E-08*	-0.469 (S)	1.000	0.061 (VS)
CSS	6.10E-10*	-0.197 (VS)	4.79E-04*	-0.370 (S)	1.000	0.076 (VS)
edgeR	1.11E-10*	-0.196 (VS)	0.029*	-0.187 (VS)	1.000	0.127 (VS)
DESeq2	2.03E-13*	-0.224 (S)	9.00E-06*	-0.397 (S)	1.000	0.052 (VS)
GMPR	7.23E-10*	-0.198 (VS)	4.23E-04*	-0.327 (S)	1.000	0.141 (VS)
None_DrImpute	2.93E-16*	-1.850 (VL)	1.000	1.238 (VL)	4.44E-12*	-2.338 (H)
None_scImpute	2.93E-16*	-0.674 (M)	1.07E-04*	-1.004 (L)	4.22E-12*	-4.819 (H)
None_LLSimpute	1.000	3.937 (H)	1.000	5.191 (H)	1.000	0.016 (VS)
None_zCompositions_SQ	1.000	0.561 (M)	1.000	0.986 (L)	1.000	0.745 (M)
None_zCompositions_CZM	1.000	0.748 (M)	1.000	1.212 (VL)	1.000	0.875 (L)
TSS_DrImpute	2.93E-16*	-2.265 (H)	1.000	0.933 (L)	4.22E-12*	-2.706 (H)
TSS_scImpute	2.93E-16*	-1.000 (L)	1.75E-05*	-1.068 (L)	4.22E-12*	-4.826 (H)
TSS_LLSimpute	1.000	3.930 (H)	1.000	4.795 (H)	0.023*	-0.332 (S)
TSS_zCompositions_SQ	3.49E-16*	-0.336 (S)	6.52E-08*	-0.469 (S)	1.000	0.061 (VS)
TSS_zCompositions_CZM	0.669	0.019 (VS)	0.664	0.244 (S)	1.000	0.471 (S)
CSS_DrImpute	2.93E-16*	-2.014 (H)	1.000	1.120 (L)	4.44E-12*	-2.334 (H)
CSS_scImpute	7.41E-16*	-0.843 (L)	2.62E-04*	-0.903 (L)	4.22E-12*	-4.659 (H)
CSS_LLSimpute	1.000	4.520 (H)	1.000	6.380 (H)	0.179	-0.181 (VS)
CSS_zCompositions_SQ	1.000	0.988 (L)	1.000	1.461 (VL)	1.000	0.108 (VS)
CSS_zCompositions_CZM	1.000	1.612 (VL)	1.000	1.444 (VL)	1.000	0.121 (VS)
edgeR_DrImpute	2.93E-16*	-2.044 (H)	1.000	1.007 (L)	4.22E-12*	-2.675 (H)
edgeR_scImpute	3.78E-16*	-0.857 (L)	0.001*	-0.842 (L)	4.22E-12*	-4.737 (H)
edgeR_LLSimpute	1.000	4.697 (H)	1.000	7.416 (H)	1.000	0.481 (S)
edgeR_zCompositions_SQ	1.000	1.259 (VL)	1.000	1.923 (VL)	1.000	0.997 (L)
edgeR_zCompositions_CZM	1.000	1.260 (VL)	1.000	1.906 (VL)	1.000	0.990 (L)
DESeq2_DrImpute	2.93E-16*	-2.092 (H)	1.000	1.210 (VL)	4.44E-12*	-2.337 (H)
DESeq2_scImpute	3.78E-16*	-0.880 (L)	1.07E-04*	-1.046 (L)	4.22E-12*	-4.751 (H)
DESeq2_LLSimpute	1.000	4.794 (H)	1.000	6.972 (H)	0.359	-0.133 (VS)
DESeq2_zCompositions_SQ	1.000	0.930 (L)	1.000	1.274 (VL)	1.000	0.128 (VS)
DESeq2_zCompositions_CZM	1.000	1.651 (VL)	1.000	2.064 (H)	1.000	0.155 (VS)
GMPR_DrImpute	2.93E-16*	-2.079 (H)	1.000	1.258 (VL)	4.44E-12*	-2.313 (H)
GMPR_scImpute	9.83E-16*	-0.873 (L)	2.62E-04*	-0.950 (L)	4.22E-12*	-4.668 (H)
GMPR_LLSimpute	1.000	4.822 (H)	1.000	5.884 (H)	1.000	0.239 (S)
GMPR_zCompositions_SQ	1.000	1.103 (L)	1.000	1.574 (VL)	1.000	0.634 (M)
GMPR_zCompositions_CZM	1.000	1.841 (VL)	1.000	2.267 (H)	1.000	0.650 (M)
